# A DFT Investigation of the Reactivity of Guanidinium Salts in Tandem aza-Michael Addition/Intramolecular Cyclization

**DOI:** 10.3390/molecules28052218

**Published:** 2023-02-27

**Authors:** Zoran Glasovac, Luka Barešić, Davor Margetić

**Affiliations:** Laboratory for Physical-Organic Chemistry, Division of Organic Chemistry and Biochemistry, Ruđer Bošković Institute, Bijenička Cesta 54, HR-10000 Zagreb, Croatia

**Keywords:** guanidine tautomers, DFT calculations, reaction mechanism, aza-Michael addition

## Abstract

A proposed mechanism of the reaction of guanidinium chlorides with dimethyl acetylenedicarboxylate in a tandem aza-Michael addition reaction/intramolecular cyclization was investigated by DFT M06-2X and B3LYP computational approaches. The energies of the products were compared against the G3, M08-HX, M11, and wB97xD data or experimentally obtained product ratios. The structural diversity of the products was interpreted by the concurrent formation of different tautomers formed in situ upon deprotonation with a 2-chlorofumarate anion. A comparison of relative energies of the characteristic stationary points along the examined reaction paths indicated that the initial nucleophilic addition is energetically the most demanding process. The overall reaction is strongly exergonic, as predicted by both methods, which is primarily due to methanol elimination during the intramolecular cyclization step producing cyclic amide structures. Formation of a five-membered ring upon intramolecular cyclization is highly favored for the acyclic guanidine, while optimal product structure for the cyclic guanidines is based on a 1,5,7-triaza [4.3.0]-bicyclononane skeleton. Relative stabilities of the possible products calculated by the employed DFT methods were compared against the experimental product ratio. The best agreement was obtained for the M08-HX approach while the B3LYP approach provided somewhat better results than the M06-2X and M11 methods.

## 1. Introduction

Guanidines are well-known organic superbases [1,2], anion recognition subunits [3], and strong nucleophiles [4] with great potential in a wide variety of applications [5]. The structure of neutral guanidine has two nitrogens of amino type and one of imino type located around the central carbon atom with electron density strongly delocalized across the CN_3_ moiety. Similarly to amines, guanidines can be alkylated with different nucleophiles, including alkyl halides [6,7,8], alcohols [9,10,11], or oxiranes [12]. However, the reaction is generally limited to the alkylation of the polysubstituted derivatives in which only one reactive center is available or chemically equivalent to other ones. Rare examples of well-defined reactions on unsymmetrically trisubstituted derivatives could be found in the literature [7,13]. In these cases, the reactivity of the different nitrogen atoms is tuned by the presence of the acyl groups and activated by strong bases such as NaH or KOH. Even then, the monoalkylation process could be performed selectively while the second alkylation provides a mixture of products due to the concurrent reactivity of the two remaining nitrogen atoms regardless of the different substituents. A similar problem with concurrent reactivity of different nitrogen atoms in guanidines is also expected in other reactions in which guanidine acts as a nucleophile.

Of special interest to us is a tendency of neutral guanidines to react with 1,3–unsaturated carbonyl compounds in aza-Michael addition [14,15,16,17,18] since they are also typical dienophiles in cycloaddition reactions. The reaction has been known for quite a long time but the early attempts to assign the structure turned out to be problematic. Some forty years ago, Acheson and Wallis analyzed structures of the products obtained by the reaction of neutral guanidines and their isothiourea siblings with dimethyl acetylenedicarboxylate (**DMAD**) and methyl propiolate [18]. Based on the detailed NMR analysis, the authors confidently assigned preferential formation of the imidazolidinone ring if acyclic guanidines were used. On the other hand, the reaction of indoline–2–thione or 2–aminothiazoles with **DMAD** provided products having the larger pyrimidinone (six-membered) ring. The concurrent formation of the 2-aminoimidazolidin-5-one and 2-amino-3-methylpyrimidin-4(3H)-one (1,6-dihydro-6-oxopyrimidine) type of products was obtained by Miyamoto and Yamazaki from unsymmetrically substituted guanidohydrazones [19]. In 2007, Shestakov et al. described the reactivity of unsymmetrically substituted guanidine with ethyl α-bromoacetate, **DMAD**, and maleic anhydride and found a preferential formation of the five-membered ring (imidazolidinone) in which the carbonyl group is always oriented toward the least substituted nitrogen atom [20]. The described results imply a mechanism of the reaction similar to that suggested by Erden et al. for the addition of amidines to **DMAD** [21]. The first step includes an attack of the monosubstituted amidine nitrogen atom to the triple bond of **DMAD** while the final step (cyclization to ester carbonyl atom) occurs after the intramolecular H-shift. The mechanism is consistent with the general knowledge of Michael addition of various nucleophiles to **DMAD** and maleic anhydride analogs [6,7,22]. In the context of the suggested mechanism, the results of Shestakov et al. imply the guanidine nitrogen atom bearing a phenyl substituent as the most nucleophilic position regardless of the electrophile. Cyclization, on the other hand, occurs at the sterically least hindered nitrogen atom.

Recently, we have found that guanidinium hydrohalides formed products of aza-Michael addition/cyclization (**AMA/CYC**) much like those obtained by the reaction of neutral guanidines with **DMAD** [23]. Unexpected reactivity was tentatively attributed to the initial deprotonation of guanidinium cation by halomaleic or halofumaric anions formed in situ by the addition of halide anions to **DMAD**. Additionally, unlike the examples found in the literature, we always obtained a mixture of isomers that vary significantly depending on the structure of the guanidinium salt employed. Besides that, intramolecular aza-Michael addition also took place after Diels–Alder reaction of furfurylguanidinium hexafluorophosphates with **DMAD**. In both cases, guanidine nitrogen atoms substituted with the isopropyl groups turned out to be practically unreactive unless a strong base is added. These results undoubtedly indicate the rich chemistry of guanidines defined by their structure. Despite the well-known and rather old chemistry described above, we are not aware of any attempts to tackle the mechanism of the reaction in more detail that would help us to understand the obtained results.

**Figure 1:**
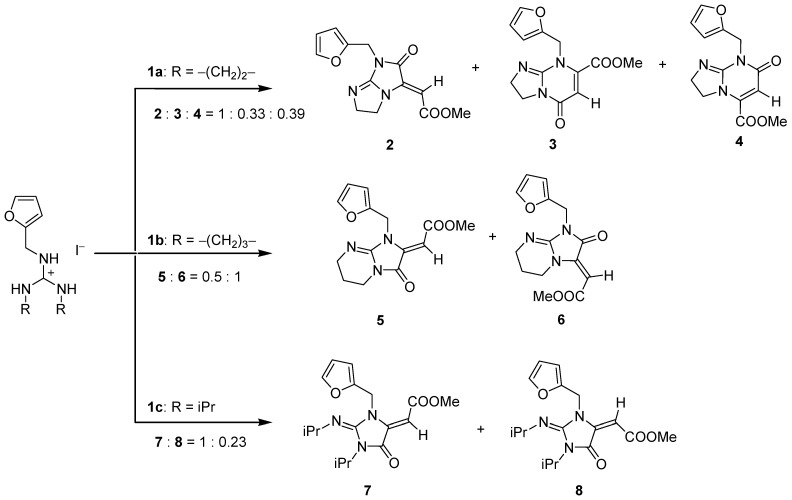
Structures of various products obtained by **AMA/CYC** tandem reaction of guanidinium salts **1a**–**1c** with **DMAD** [23]. Relative ratios are calculated from the ^1^H NMR spectra of the reaction mixtures.

The structures of products obtained from salts **1a** and **1b** (Figure 1) strongly indicate the concurrent reactivity of two nitrogen atoms as nucleophiles in the initial stage of the reaction. This was not observed with diisopropyl derivative **1c**. This poses the question of whether the reaction is governed primarily by the thermodynamic stability of the reactants and products or by the reaction kinetics. Here, we should emphasize that the mentioned products are the most abundant ones in the reaction mixture that we were able to isolate and identify without doubt. Some minor amounts of other products (below 5%) could be present in the mixture but they were neglected.

To rationalize the origin of the observed reactivity, we employed the DFT computational approach. Since the description of the structure of neutral guanidines as an equilibrium of several tautomers is generally accepted [24], the initial stage of the reaction was analyzed by considering the relative stabilities of different guanidine tautomers. The correlation between the stability of the tautomers and their reactivity toward electrophiles is assumed. The proposed reaction mechanism is further investigated by calculating corresponding transition state (TS) structures and the Gibbs energies of the reactions along the reaction paths. Besides rationalization of the origin of the structural diversity of products, the accuracy of the DFT methods to predict the product ratios is also assessed.

## 2. Results

A computational approach to the analysis of the tandem **AMA/CYC** reaction was started by considering the thermodynamics of the hydrogen halide transfer from guanidinium halide to **DMAD**. The assumed mechanism of the reaction is initiated by the halide attack on **DMAD** followed by proton abstraction from the guanidinium cation as we proposed recently [23]. The overall reaction is shown in Figure 1. We should emphasize that maleate derivative could also be formed but we limited our analysis to the formation of the more stable chlorofumarate **9** for the sake of simplicity. Deprotonation of the guanidinium cation by the chloride or iodide anion could be disregarded because we have shown that no aza-Michael addition products were obtained when *N*-phenylmaleimide was used instead of **DMAD** [25]. In this context, we could also note that the gas-phase basicities (GB) of 2-chlorofumarate and 2-chloromaleate anions calculated using B3LYP (1477 and 1469 kJ mol^−1^, respectively) are significantly higher than that of chloride anions (1388 kJ mol^−1^; experimental value is 1374 kJ mol^−1^ [26]). The large difference in the basicities is most likely preserved in the acetonitrile solution, which implies that once the chloride anion is captured by **DMAD**, proton transfer to the formed chlorofumarate (or chloromaleate) anion is highly favored.

**Figure 2:**
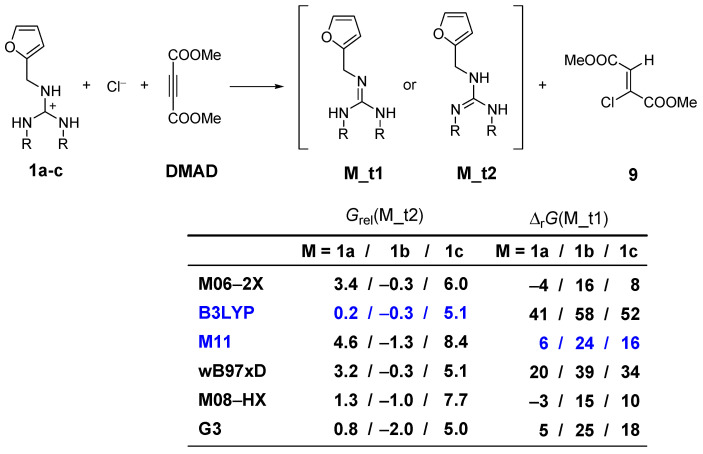
Reaction scheme and energetics for the formation of two possible tautomers by the hydrogen chloride transfer from the guanidinium salt to **DMAD**. Relative energies of the tautomer **M_t2** are given with respect to **M_t1**. All energies are given in kJ mol^−1^. The results closest to the G3 values are given in blue color.

Two non-equivalent tautomeric forms of the neutral guanidines (**M_t1** and **M_t2**, Figure 2) could be formed upon deprotonation of the corresponding guanidinium cations. Their relative energies (*G*_rel_) and the Gibbs energies of the reactions (Δ_r_*G*) were calculated using five selected DFT methods. Two commonly employed density functionals were considered as very good (M06-2X [27]) and poor (B3LYP [28,29,30,31]) performers in the reaction Gibbs energies [32,33], and reaction barriers [34]. Besides them, three other density functionals (M11 [35], wB97xD [36], and M08-HX [37]), recommended for the reaction kinetics and thermodynamics, were also tested [35,38]. The computed relative energies were benchmarked against G3 [39] results. The results are given in Figure 2. Since in our previous paper, B3LYP correctly identified the isolated imidazolidinone derivatives **2–4** (Figure 1) as the most stable isomers of six possible products [23], we wanted to test its performance in spite of its known shortcomings, primarily from the viewpoint of the relative energies of the isomeric structures. The obtained results for the stability of tautomers are generally consistent across the employed methods. When compared to G3 results, the relative stabilities of two tautomers are interpreted slightly better with B3LYP than with other DFT methods although all results are within the margin of the chemical accuracy of the employed methods (within 4 kJ mol^−1^). These results justify our choice to include B3LYP in further calculations. Somewhat surprising are the very similar energies of the tautomers **1a_t1** and **1a_t2**. One could expect that the strain of the five-membered (imidazolidine) ring will favor the tautomer having an exocyclic bond [40]; the intrinsic difference in their stability is apparently reduced by the solvation. In the absence of the ring strain (**1b**), practically no difference in the stabilities of the tautomers was obtained by most of the DFT calculations while subtle preference was given to the **1b_t2** tautomer by the G3 method. The largest difference in energies calculated for tautomers **1c_t1** and **1c_t2** could be ascribed to the electronic effects of the isopropyl group which enhances the basicity of amidines and guanidines offer the simple alkyl derivatives in the gas-phase and acetonitrile [41,42]. This result is qualitatively in agreement with the absence of the products that would be obtained from the **t2** tautomer.

Similarly to the results for the cycloaddition, the reaction Gibbs energies calculated by the employed DFT methods differ strongly. The B3LYP-calculated reaction energies are unrealistically too endothermic, as expected [23,34], while the M06-2X results overestimate the stability of the products by ca 10 kJ mol^−1^. G3 results are positioned between these two functionals, being closer to M06-2X. Of the other three DFT methods, M11 provided an excellent agreement with the G3 results but the relative stabilities of the tautomers showed quite large deviations. On the other hand, mutually similar and slightly better results than those of M11 were obtained by M06-2X and M08-HX. With this in mind, we approached the investigation of the reaction paths for the **AMA/CYC** reaction between each guanidine tautomer and **DMAD** using B3LYP and M06-2X functionals.

### Addition of Neutral Guanidines to **DMAD**

**Scheme 1:**
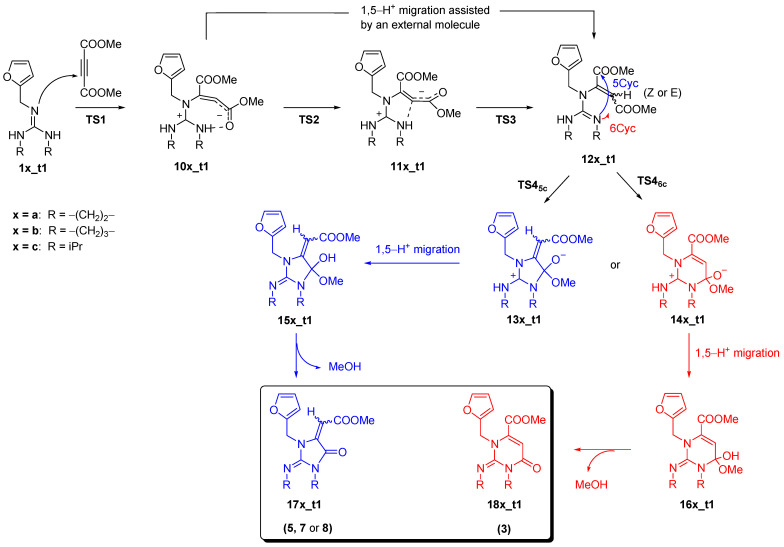
Proposed mechanism for the **AMA/CYC** tandem reaction starting from the neutral guanidines **1a_t1**–**1c_t1**. Analogous mechanism starting from **1a_t2** or **1b_t2** leads to product **2**, **4**, or **6**.

Kinetics and the overall thermodynamics of two possible reaction pathways for each guanidine derivative were investigated by the above-mentioned selected DFT approaches. The adopted reaction mechanism is a variant of that proposed by Erden et al. as noted in the Introduction [21] and it is shown in Scheme 1. The stationary points along the proposed pathways were optimized using the B3LYP approach while energies were calculated employing B3LYP and M06-2X functionals in combination with the selected basis sets as defined in the Computational details.

**Figure 3:**
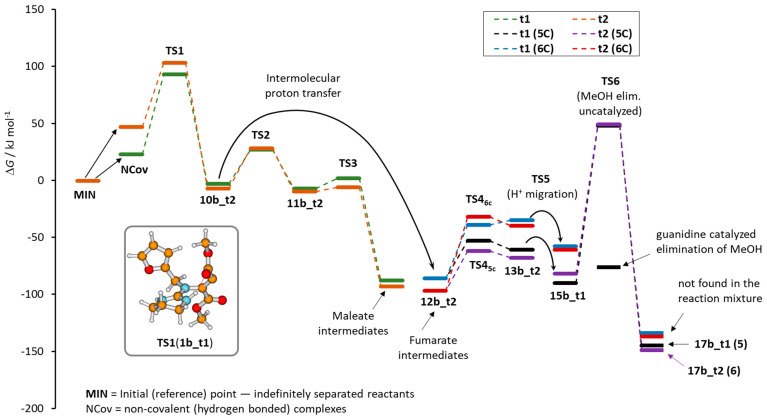
Energy profile for the reaction of the tautomers **1b_t1** and **1b_t2** with **DMAD**. The labels 5C and 6C relate to the ring size formed upon the intramolecular cyclization step. Only the lowest energy structures are labeled.

The Gibbs energies calculated for all minima and the selected transition state structures are given in Table 1, while the data obtained for the **1b_t1**/**1b_t2** series are also presented as the reaction energy profile shown in Figure 3. For the sake of clarity, the reaction profile does not include several minima and TS structures that are due to the conformational changes needed to follow the minimum energy reaction path. All energies were calculated relative to the infinitely separated reacting guanidines (tautomers **1x_t1**) and **DMAD**. The structure labels used throughout the text refer to the *Z* isomers. When appropriate, the prefix *E-* is used to indicate the structures formed after fumarate/maleate interconversion.

First, we shall tackle the thermodynamics of the reaction. The overall reaction is highly exergonic due to the strong stabilization of the system upon the formation of a fumarate intermediate (**12**(**a−c**)) and the elimination of methanol. The calculated Gibbs energies of the reactions are given in Table 1 and the structures of the products are in very good agreement with those of the most abundant products obtained experimentally.

The two employed DFT computational approaches perform similarly. The only exception is the structure **17c_t2** which is preferred over the *E*-isomer of **17c_t1** by B3LYP which is opposite to the M06-2X result but consistent with the experimental finding [23]. Here, we add that both approaches similarly predict comparable or slightly lower energies of *E*-isomers of other **17**(**a−c**) derivatives, however, we should emphasize that the products were optimized as complexes with the molecule of methanol for the sake of atom consistency with other structures, which may affect their relative energies significantly (see below). We note in passing that the *E*-isomer of any of the investigated products is observed only in the case of acyclic guanidine **1c** when the geometry of the guanidine moiety prefers the *C*_3_ structure with NH bonds oriented under the angle of 120° [23]. To gain further insight into the reaction mechanism and similarities and differences in the employed DFT methods, we analyzed all important stationary points along the reaction paths starting from three pairs of guanidine tautomers. Here, we should emphasize that the Gibbs energies of all structures were not corrected for the low-energy vibrations that could be particularly important for the non-covalently bound associates (**NCov** type of the structure in Figure 3). Applying the correction according to the procedure proposed by Truhlar [43] lowered the energy of all stationary points (Section S4 in Suppl. Mat.) but the qualitative picture remained unchanged. Additionally, we would like to point out that the **NCov** structures are not necessarily the lowest energy minima of the non-covalent associates but rather the first ones obtained by following the intrinsic reaction coordinate toward the reactants. These two factors are the reasons for the unexpectedly high relative energy of the **NCov** structures.

The reaction mechanism could be divided into two sections: the aza-Michael addition (**AMA**) and cyclization (**CYC**). The calculation predicts the nucleophilic attack of neutral guanidine on **DMAD** to be energetically the most demanding step of the **AMA** section. In all computed structures, the barrier for the initial nucleophilic attack (**TS1**) from the furfuryl-substituted nitrogen atom is lower in energy and the difference ranges from 3 kJ mol^−1^ (**1b_t1**/**1b_t2**) to 14 kJ mol^−1^ for the **1c_t1**/**1c_t2** pair with M06-2X providing a slightly smaller range but larger energy differences than B3LYP. The large difference in the calculated barriers for the reaction of **1c** tautomers indicates that the **t2** reaction channel will not be populated and the products will predominantly be formed from **1c_t1** tautomers. As noted in the previous section, the relative stabilities of these two tautomers (Figure 2) additionally support such reasoning. Along with that, the relative stabilities of the isomers **10c_t1** and **10c_t2** also support the predominance of the **t1** path. The differences in barrier heights calculated for tautomers of the other two guanidine derivatives are rather small at the B3LYP level of theory albeit a slight preference for the **t1** path is indicated. Nevertheless, concurrent reactivity is expected in both cases. On the other hand, M06-2X predicts comparable reactivities of the **1b_t1**/**1b_t2** pair to those of the **1c_t1**/**1c_t2** pair. That would mean no or low abundance of the products formed initiated by the nucleophilic attack of **1b_t2** tautomer. This result contrasts with the experimentally observed ratio of the products (Figure 1). The stability of the intermediates **10a_t1** and **10a_t2** as well as their **1b** analogs indicates the preference for the **t2** path as consistently predicted by both DFT approaches. Up to this point, the reaction is predicted to be practically thermoneutral (M06-2X) and could be considered reversible.

The stability of so-formed “zwitterionic” intermediates **10**(**a−c**) is further increased upon conversion to maleate or fumarate derivatives (**11**(**a−c**) **and 12**(**a−c**), respectively). The process includes proton transfer from the guanidinium moiety to the newly formed center with a partially anionic character. Modeling the intramolecular proton transfer between the guanidinium and fumarate anion subunits resulted in inversion at the anionic center and structural reorganization to maleates. Inspection of the isolated product structures shown in Figure 1 reveals that maleate intermediate could be responsible for the formation of product **8** only. Besides that, their configuration does not allow cyclization to the remote carboxylic group under a low-energy regime while the cyclization to the proximate carbonyl atom is assumed to be similar to the fumarate isomers. Therefore, maleate isomers were neglected from further TS structure calculations but the structures and energies of the corresponding minima were calculated (Supplementary Materials). The intermediates **10**(**a−c**) are already pre-organized to produce fumarates by direct protonation and they are most likely formed by the proton transfer mediated by some component in the reaction mixture (moisture, methanol as a by-product of the reaction, or another molecule of guanidine that assists the intramolecular 1,5-hydrogen shift). Regardless, this step is not considered to affect the reaction kinetics.

The second section of the reaction path (**CYC** section) encompasses cyclization to either five-membered (5C) or six-membered (6C) cyclic intermediates. The barrier to the elimination of methanol dominates this part of the profile. Here, we should note that the calculated barrier relates to the intramolecular proton transfer that involves the four-membered cyclic TS structure which is known to be highly unfavorable. The elimination of methanol is a process that can be assisted by the external molecule(s) of water or the other proton-donating molecules which can significantly reduce the reaction barriers as shown by Antol et al. [44]. Indeed, assuming the guanidine itself as the catalyst for the conversion of **15b_t1** to **17b_t1**, the calculated barrier for the elimination of methanol was lowered from 88 kJ mol^−1^ to −72 kJ mol^−1^ (see Section S3 in the Supplementary Materials). Therefore, this step cannot be considered the critical one.

The other barriers calculated along the investigated reaction paths are significantly lower than **TS1** and they are not considered to affect the reaction rates. However, we can briefly compare barriers for the ring closure to five- (5C) or six-membered (6C) rings. Formation of the smaller ring will be preferred mostly and the only exception is tautomer **1a_t1**. In this case, the ring closure to the intermediate **13a_t1** is the most energetically demanding with the barrier of 67 (71) kJ mol^−1^ with respect to **12a_t1** as calculated by M06-2X (B3LYP) approaches. For the diisopropyl guanidine series, we were not able to optimize the minimum that would correspond to **14c_t2** so the comparison of the barriers for the smaller and larger ring closure cannot be conducted. The barriers to the formation of the five-membered intermediate **13c** are comparable to those for **13a** and **13b**.

Finally, we employed five selected DFT approaches (see Computational details) to compare the calculated product ratios with those obtained experimentally. In contrast to data given in Table 1, the results presented here were obtained for the methanol-free structures. The best agreement with the experimental ratios was obtained with the M08-HX approach (Table 2, for the complete results, see Table S3 in Supplementary Materials). Excellent agreement was obtained for derivatives obtained from **1b** and **1c** tautomer pairs while the results for **18a_t1** and **18a_t2** overestimate their abundance. The same trend was obtained with other DFT methods but with even more emphasized differences in the ratios. Surprisingly, B3LYP was second-best approach and the only method other than M08-HX that predicted a significant amount of **17b_t2** product. We could note that, irrespective of the employed DFT method, the relative energies of *E*-**17a** and *E*-**17b** are consistent with their absence from the reaction mixture.

## 3. Discussion and Conclusions

The results presented in the previous section provide several important features that explain the reactivity of guanidinium hydrohalides with **DMAD** and define the product ratio. The reactions are initiated by thermoneutral or slightly endergonic addition of the hydrogen chloride to **DMAD** followed by highly exergonic tandem aza-Michael addition/intramolecular cyclization. Within the tandem process, the aza-Michael addition step is calculated as thermoneutral until stabilization by direct or externally mediated proton transfer takes place. This result qualitatively agrees with the known reversibility of the addition of the persubstituted guanidines to the unsaturated systems [45] which allows their use as organocatalysts even in the presence of strong electrophiles [46,47]. The observed product ratios are mainly consistent with their calculated relative Gibbs energies. The main difference arises from the low abundance or the lack of the products coming from the cyclization of the maleate intermediate *E*-**12(a**–**c)**. Literature data on the **DABCO**-catalyzed addition of various nucleophiles to **DMAD** implies strong dependence of the *E*/*Z* ratio of the adducts on the structure of nucleophiles [48]. The reaction of amides, carboxylates, and amino alcohols with **DMAD** provide fumarate derivatives, exclusively, while the alcohols produce a mixture of fumarates and maleates. The strong predominance of the products arising from the fumarate type intermediate structure implies the similarity of the nucleophilic activity of guanidines to amides and carboxylates. As already mentioned, the intramolecular hydrogen bonding occurring in the **10**(**a–c**) intermediates additionally assists the formation of the fumarate intermediates if the external source of a proton is available. Once formed, the preference of the cyclization to the proximate or remote carboxylic group is regulated by the stability of the products.

The initial addition step is characterized as the most energy-demanding process and the barriers (TS1) are expected to follow the nucleophilicities of the guanidine tautomers. The literature data on 1,5,7-triazabicyclo[4.4.0]-dec-1-ene (**TBD**) and 1,4,6-triazabicyclo[3.3.0]oct-4-ene (**TBO**) imply higher nucleophilicity of the larger ring system [4,49]. Further, the nucleophilicity of the exocyclic nitrogen in 2-aminoimidazolidine derivatives is slightly lower than the analogous position in acyclic derivatives [4]. Additionally, isopropyl groups are known to reduce the nucleophilicity of amines with respect to alkyl or benzyl groups [50]. Based on these data, we could expect the following trends in the reactivity of tautomers: **1b_t2** > **1a_t2**, **1c_t1** > **1a_t1**, and **1c_t1** > **1c_t2**. Generally, computed energies of TS1 structures do not follow these trends except for TS1 (**1c_t2**) where we expect the slowest reaction. Apparently, the effects other than the simple nucleophilicity of guanidines play an important role.

As a conclusion, we could emphasize that the product structure and distribution are primarily determined by the thermodynamics of the reaction rather than by the relative stability of the guanidine tautomers. The kinetics of the reaction is probably just tuning the relative ratio. The performance of the employed DFT methods was tested against G3 benchmark results or against the experimental product ratio. The M11 approach performs the best in terms of the reaction energies for the addition of HCl to **DMAD**. However, it fails to predict the correct trend in product distribution. Among the other DFT methods, M08-HX provided relative energies of the products that are in very good agreement with the experimentally observed data. Besides that, it gives good estimates of the relative stabilities of the guanidine tautomers, and slightly more exergonic addition of HCl to DMAD than M11. Often criticized, the B3LYP method interpreted the relative energies of the guanidine tautomers as well as the product distribution better than M11 and wB97xD and it was at least comparable to M06-2X. Additionally, the conclusions that could be drawn from the reaction path calculations are essentially the same as those obtained from M06-2X results. For example, the relative stabilities of intermediates along the reaction paths obtained by B3LYP and M06-2X are comparable except that B3LYP results are systematically shifted higher in energy by more than 20 kJ mol^−1^.

## 4. Materials and Methods

Computational details: All the structures were fully optimized at the B3LYP/6-31G(d,p) level of theory [28,29,30,31] in acetonitrile and the nature of the stationary points was confirmed with vibrational analysis (NImag = 0 or NImag = 1 for the TS structures). The solvent was approximated as the dielectric continuum and treated employing the SMD approach (*ε*_0_(CH_3_CN) = 35.688) [51]. The Gibbs correction to energy was taken from the vibrational analysis and used without any additional corrections or scaling. Electronic energies were refined by the single point calculations at B3LYP/6-311+G(2df,p) or M06-2X/6-311++G(3df,2pd) levels of theory [27]. These approaches are referred to as B3LYP and M06-2X throughout the text. Another three DFT approaches (M11 [35], wB97xD [36], and M08-HX [37]) encompassed re-optimization of the structure with subsequent single point calculations as follows:

**M11**: M11(SMD)//aug-cc-pVTZ//M11(SMD)/6-31+G(d,p)

**wB97xD**: wB97xD(SMD)/aug-cc-pVTZ//wB97xD(SMD)/6-31+G(d,p)

**M08-HX**: M08-HX(SMD)/pcseg-3//M08-HX(SMD)/pcseg-2

In the case of M06-HX, Jensen’s polarization consistent segmented contracted basis sets of double zeta (pcseg-2) and triple zeta (pcseg-3) quality [52] taken from the EMSL/PNNL Basis Set Exchange repository [53] were used. All calculations were performed using Gaussian16 [54]. Visualization of the optimized structures was carried out with MOLDEN 5.9 [55,56].

## Figures and Tables

**Table 1:** The relative Gibbs energies calculated for the selected structures along the reaction paths ^1^.

Guandines	Method	Δ*G^≠^* (TS1)	Δ_r_*G* (10a–10c)	Δ_r_*G* (12a–12c)	Δ*G^≠^* (TS4_5C_)	Δ*G^≠^* (TS4_6C_)	Δ_r_*G* (Prod) ^2^ (17a–17c)	Δ_r_*G* (Prod) (18a–18c)
	B3LYP							
**1a_t1**		117	54	−34	37	32	−87 (−83)	−100
**1a_t2**		122	45	−56	−3	14	−102 (−89)	−107
**1b_t1**		119	53	−40	−1	(27) ^3^	−114 (−114)	−92
**1b_t2**		122	38	−44	−3	36	−120 (−114)	−96
**1c_t1**		132	86	8	50	i.p. ^3^	−80 (−72)	−23
**1c_t2**		146	94	7	42	r.o. ^4^	−75 (−66)	−26
	M06-2X							
**1a_t1**		94	7	−84	−19	−30	−112 (−107)	−137
**1a_t2**		100	5	−99	−53	−43	−126 (−110)	−141
**1b_t1**		92	−3	−93	−53	(−39) ^3^	−145 (−144)	−134
**1b_t2**		103	−7	−94	−59	−32	−149 (−143)	−137
**1c_t1**		99	22	−64	−19	i.p. ^3^	−114 (−112)	−73
**1c_t2**		108	29	−57	−23	r.o. ^4^	−107 (−99)	−78

^1^ All energies are given in kJ mol^−1^; the labels 5C and 6C refer to the ring size formed upon the intramolecular cyclization step. ^2^ All structures of products were calculated as the complexes with methanol; values in parentheses refer to the *E*-isomers of the corresponding structures. ^3^ i.p. = inflection point, the relaxed scan calculations starting from the **13c** structures indicated a flat region of the potential energy surface with NImag = 0 at several examined points within this region. ^4^ r.o. = ring opening (no stationary point located).

**Table 2:** The relative Gibbs energies (**Δ*G*_rel_**) of the possible isomeric reaction products calculated using the M08-HX approach ^1^.

Guan.	Δ*G*_rel_ ^1^	Product	Ratios	Guan.	Δ*G*_rel_ ^1^	Product	Ratios	Guan.	Δ*G*_rel_ ^1^	Product	Ratios
		calc.	exp.			calc.	exp.			calc.	exp.
**17a_t1**	21.7	0.00	-	**17b_t1**	2.1	0.44	0.50	**17b_t1**	0.0	1.00	1.00
*E*-**17a_t1**	33.3	0.00	-	*E*-**17b_t1**	10.2	0.02	-	*E*-**17b_t1**	2.7	0.34	0.23
**17a_t2**	4.3	**0.18**	1.00	**17b_t2**	0.0	1.00	1.0	**17b_t2**	9.0	0.03	-
*E*-**17a_t2**	25.9	0.00	-	*E*-**17b_t2**	8.5	0.03	-	*E*-**17b_t2**	11.6	0.01	-
**18a_t1**	0.9	**0.69**	0.33	**18b_t1**	14.6	0.00	-	**18b_t1**	48.3	0.00	-
**18a_t2**	0.0	**1.00**	0.39	**18b_t2**	18.1	0.00	-	**18b_t2**	43.5	0.00	-

^1^ Relative Gibbs energies are given in kJ mol^−1^; the relative Gibbs energies were calculated against the most stable isomer.

## Data Availability

The data are available in this publication and Supplementary Materials.

## Supplementary Materials

The following supporting information can be downloaded at: https://www.mdpi.com/article/10.3390/molecules28052218/s1, Scheme S1: Reaction scheme for the formation of 2-chlorofumarate (**9H**) by chloride addition to **DMAD** with concomitant deprotonation of the guanidine. Table S1: Calculated electronic energies (*E*_scf_), corrections to the Gibbs energies (*G*_corr_), and Gibbs energies (*G*_tot_) of the relevant structures associated with the reaction given in Scheme S1, and calculated using B3LYP, M06-2X, and G3 approaches; Table S2: Calculated electronic energies (*E*_scf_), corrections to the Gibbs energies (*G*_corr_), and Gibbs energies (*G*_tot_) of the relevant structures associated with the reaction pathway starting from the guanidine tautomers. Figure S1: Energy profile for the AMA/CYC reaction of the tautomers of furfurylguanidine **1a**. Only the lowest energy points are labeled; Figure S2: Energy profile for the AMA/CYC reaction of the tautomers of furfurylguanidine **1c**. Only the lowest energy points are labeled; Figure S3. The first part of the uncorrected (solid lines) and corrected (dashed lines) reaction profiles starting from **1b_t1** (green) and **1b_t2** (yellow) tautomers; Figure S4. The second part of the uncorrected (solid lines) and corrected (dashed lines) reaction profiles starting from **1b_t1** (black) and **1b_t2** (purple) tautomers; Table S3. The product abundances (Y) relative to the most stable one calculated by different DFT approaches using Equation (S1); Figure S5: Structure of the guanidine catalyst **6rMe** representing the simplified **1b_t1** structure; Figure S6: Calculated relative Gibbs energies (Δ*G*_rel_) for the methanol elimination step catalyzed with guanidine. The values are calculated relative to the sum of the Gibbs energies of the guanidine **1b_t1**, **DMAD**, and guanidine catalyst **6rMe**. Cartesian coordinates of all optimized stationary points are also given [34,38,43].

## Sample Availability

Not relevant to this study.
