# Supplementary material for: A DFT Investigation of the Reactivity of Guanidinium Salts in Tandem aza-Michael Addition/Intramolecular Cyclization
Source: Molecules. 2023 Feb 27;28(5):2218. doi: 10.3390/molecules28052218 (PMC10005421; doi:10.3390/molecules28052218)
Supplement: Supplementary file 1 [file molecules-28-02218-s001.zip › molecules-2222504-supplementary.pdf]

Supplementary Material

A DFT Investigation of the Reactivity of Guanidinium Salts in Tandem aza-Michael Addition/Intramolecular Cyclization

Zoran Glasovac *, Luka Barešić and Davor Margetić

S1. Formation of the guanidine tautomers 2

S2. Mechanism of the **AMA/CYC** reaction 3

S2.1. Energies of the investigated structures 3

S3. The reaction energy profiles calculated for **1a** and **1c** 9

S4. The Entropy correction approach 10

S5. Comparison of selected density functionals 12

S6. The methanol elimination catalyzed with guanidine 14

S7. Cartesian coordinates 15

S7.1. G3 Optimized geometries 15

S7.2. B3LYP Optimized geometries (from 1a tautomers) 21

S7.3. B3LYP Optimized geometries (from 1b tautomers) 52

S7.4. B3LYP Optimized geometries (from 1c tautomers) 84

S8. References 122

S1. Formation of the guanidine tautomers

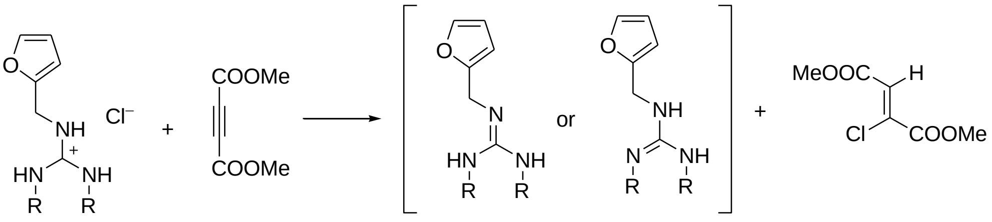

1a-c **DMAD** **M_t1** **M_t2** **9H**

Scheme S1. Reaction Scheme for the formation of 2-chlorofumarate (**9H**) by chloride addition to **DMAD** with concomitant deprotonation of the guanidine.

Table S1. Calculated electronic energies (E_{scf}), corrections to the Gibbs energies (G_{corr}) and Gibbs energies (G_{tot}) of the relevant structures associated with the reaction given in Scheme S1, and calculated using B3LYP, M06-2X and G3 approaches. [1,2]

Structure	E_{scf}(B3LYP)	G_{corr}	G_{tot} (B3LYP)	E_{scf}(M06)	G_{tot} (M06)	G_{tot}(G3)
**1a_t1**	−551.12745	0.15260	−550.97485	−550.91208	−550.75948	−550.58275
**1a_t2**	−551.12689	0.15210	−550.97479	−550.91027	−550.75817	−550.58246
**1a**H⁺	−551.60587	0.16645	−551.43943	−551.38441	−551.21796	−551.04515
**1b_t1**	−590.45549	0.18059	−590.27490	−590.22084	−590.04025	−589.85684
**1b_t2**	−590.45523	0.18021	−590.27501	−590.22058	−590.04037	−589.85762
**1b**H⁺	−590.93913	0.19332	−590.74581	−590.69966	−590.50635	−590.32688
**1c_t1**	−709.64338	0.27518	−709.36820	−709.34168	−709.06650	
**1c_t2**	−709.64199	0.27572	−709.36627	−709.33991	−709.06420	
**1c**H⁺	−710.12810	0.29144	−709.83666	−709.82090	−709.52947	−709.34213
**DMAD**	−533.28148	0.07614	−533.20534	−533.07424	−532.99809	−532.80517
**Cl-**	−460.40868	−0.01502	−460.42370	−460.37370	−460.38872	−460.24342
**9H**	−994.16659	0.08874	−994.07785	−993.93546	−993.84672	−993.50921

[1] B3LYP = B3LYP(SMD,ACN)/6-311+G(2df,p)//B3LYP(SMD,ACN)/6-31G(d,p); M06 = M06-2X(SMD,ACN)/6-311++G(3df,2pd) //B3LYP(SMD,ACN)/6-31G(d,p), G3 = G3(SMD,ACN). [2] Energies, $G_{corr,}$ and G_{tot} were given in a.u. ΔG_{rel} is given in kJ mol^{-1}.

S2. Mechanism of the **AMA/CYC** reaction

S2.1. Energies of the investigated structures

Table S2. Calculated electronic energies (E_{scf}), corrections to the Gibbs energies (G_{corr}) and Gibbs ener-gies (G_{tot}) of the relevant struc-tures associated with the reaction pathway starting from the guanidine tautomers. [1,2.]

Structure	E_{scf}(B3LYP)	G_{corr}	G_{tot} (B3LYP)	E_{scf}(M06)	G_{tot} (M06)	ΔG_{rel}(M06) [3]
**NCov(1a_t1)**	−1084.40848	0.24775	−1084.16073	−1083.99151	−1083.74376	36
**TS1(1a_t1)**	−1084.38631	0.25053	−1084.13579	−1083.97220	−1083.72167	94
**10a_t1**	−1084.41656	0.25665	−1084.15991	−1084.01191	−1083.75526	6
**TS2(1a_t1)**	−1084.40525	0.25479	−1084.15046	−1083.99846	−1083.74368	37
**11a_t1**	−1084.41634	0.25566	−1084.16068	−1084.01218	−1083.75653	3
**TS3(1a_t1)**	−1084.40990	0.25254	−1084.15737	−1084.00555	−1083.75301	12
E-**12a_t1**	−1084.40990	0.25254	−1084.15737	−1084.04325	−1083.78741	−78
Z-**12a_t1**	−1084.44936	0.25627	−1084.19309	−1084.04568	−1083.78941	−84
**TS4$_{5C}$(1a_t1)**	−1084.42578	0.25757	−1084.16821	−1084.02251	−1083.76494	−19
**TS4$_{6C}$(1a_t1)**	−1084.42677	0.25880	−1084.16797	−1084.02770	−1083.76891	−30
**13a_t1**	−1084.42740	0.25809	−1084.16930	−1084.02493	−1083.76684	−24
E-**13a_t1**	**ring open-ing**					
**14a_t1**	−1084.42868	0.25873	−1084.16995	−1084.02966	−1083.77093	−35
**15a_t1**	−1084.43388	0.25938	−1084.17451	−1084.03921	−1083.77984	−58
E-**15a_t1**	−1084.44658	0.25992	−1084.18666	−1084.05125	−1083.79132	−89
**16a_t1**	−1084.43354	0.25887	−1084.17467	−1084.04170	−1083.78283	−66
**17a_t1**	−1084.46626	0.25297	−1084.21329	−1084.05335	−1083.80038	−112
E-**17a_t1**	−1084.46297	0.25118	−1084.21179	−1084.04959	−1083.79841	−107
**18a_t1**	−1084.47207	0.25364	−1084.21843	−1084.06322	−1083.80957	−137

[1] B3LYP = B3LYP(SMD,ACN)/6-311+G(2df,p)//B3LYP(SMD,ACN)/6-31G(d,p); M06 = M06-2X(SMD,ACN)/6-311++G(3df,2pd) //B3LYP(SMD,ACN)/6-31G(d,p). [2] E_{scf}, G_{corr}, and G_{tot} are given in a.u. ΔG_{rel} is given in kJ mol^{-1}.
ΔG_{rel} is calculated using the equation: ΔG_{rel} (Structure) = G_{tot}(Structure) - [G_{tot}(**1a_t1**) + G_{tot}(**DMAD**)]

Table S2. (contd.)

Structure	E_{scf}(B3LYP)	G_{corr}	G_{tot} (B3LYP)	E_{scf}(M06)	G_{tot} (M06)	ΔG_{rel}(M06) [3]
**NCov(1a_t2)**	−1084.40601	0.24675	−1084.15926	−1083.98737	−1083.74062	45
**TS1(1a_t2)**	−1084.38402	0.25012	−1084.13390	−1083.96966	−1083.71955	100
**10a_t2**	−1084.41974	0.25629	−1084.16345	−1084.01203	−1083.75574	5
**TS2(1a_t2)**	−1084.40853	0.25447	−1084.15406	−1083.99898	−1083.74451	34
**11a_t2**	−1084.42096	0.25466	−1084.16630	−1084.01342	−1083.75875	−3
**TS3(1a_t2)**	−1084.41893	0.25176	−1084.16717	−1084.01161	−1083.75985	(−6) [4]
E-**12a_t2**	−1084.45764	0.25665	−1084.20098	−1084.05259	−1083.79594	−101
Z-**12a_t2**	−1084.45806	0.25661	−1084.20145	−1084.05182	−1083.79521	−99
**TS4$_{5C}$(1a_t2)**	−1084.43855	0.25541	−1084.18315	−1084.03328	−1083.77787	−53
**TS4$_{6C}$(1a_t2)**	−1084.43208	0.25710	−1084.17499	−1084.03107	−1083.77397	−43
**13a_t2**	−1084.43922	0.25533	−1084.18390	−1084.03498	−1083.77966	−58
E-**13a_t2**	**ring opening**					
**14a_t2**	−1084.43295	0.25761	−1084.17534	−1084.03205	−1083.77446	−44
**15a_t2**	−1084.44078	0.25714	−1084.18365	−1084.04478	−1083.78798	−80
E-**15a_t2**	−1084.44833	0.25878	−1084.18955	−1084.05165	−1083.79287	−93
**16a_t2**	−1084.43437	0.25751	−1084.17686	−1084.04121	−1083.78370	−69
**17a_t2**	−1084.47084	0.25192	−1084.21892	−1084.05733	−1083.80541	−126
E-**17a_t2**	−1084.46445	0.25017	−1084.21427	−1084.04948	−1083.79930	−110
**18a_t2**	−1084.47396	0.25299	−1084.22097	−1084.06412	−1083.81113	−141

[1] B3LYP = B3LYP(SMD,ACN)/6-311+G(2df,p)//B3LYP(SMD,ACN)/6-31G(d,p); M06 = M06-2X(SMD,ACN)/6-311++G(3df,2pd) //B3LYP(SMD,ACN)/6-31G(d,p). [2] E_{scf}, G_{corr}, and G_{tot} are given in a.u. ΔG_{rel} is given in kJ mol^{-1}. ΔG_{rel} is calculated using the equation: ΔG_{rel} (Structure) = G_{tot}(Structure) - [G_{tot}(**1a_t2**) + G_{tot}(**DMAD**)]. 4 Interconversion from the fumarate to maleate anion substructure is associated with the barrierless intramolecular proton transfer from the guanidine subunit.

Table S2. (contd.)

Structure	E_{scf}(B3LYP)	G_{corr}	G_{tot} (B3LYP)	E_{scf}(M06)	G_{tot} (M06)	ΔG_{rel}(M06) [3]
**NCov(1b_t1)**	−1123.73579	0.27347	−1123.46232	−1123.30238	−1123.02974	23
**TS1(1b_t1)**	−1123.71342	0.27838	−1123.43504	−1123.28164	−1123.00326	92
**10b_t1**	−1123.74695	0.28566	−1123.46129	−1123.32499	−1123.03933	−3
**TS2(1b_t1)**	−1123.73506	0.28263	−1123.45243	−1123.31061	−1123.02798	27
**11b_t1**	−1123.74643	0.28375	−1123.46268	−1123.32406	−1123.04031	−5
**TS3(1b_t1)**	−1123.74007	0.27989	−1123.46019	−1123.31735	−1123.03746	2
E-**12b_t1**	−1123.77562	0.28086	−1123.49476	−1123.35280	−1123.07194	−88
Z-**12b_t1**	−1123.78028	0.28468	−1123.49560	−1123.35847	−1123.07379	−93
**TS4$_{5C}$(1b_t1)**	−1123.76549	0.28474	−1123.48075	−1123.34339	−1123.05865	−53
**TS4$_{6C}$(1b_t1)**	−1123.75496	0.28514	−1123.46982	−1123.33817	−1123.05303	−39
**13b_t1**	−1123.76803	0.28565	−1123.48238	−1123.34720	−1123.06155	−61
E-**13b_t1**	**ring opening**					
**14b_t1**	−1123.75382	0.28552	−1123.46830	−1123.33732	−1123.05181	−35
**15b_t1**	−1123.77238	0.28691	−1123.48546	−1123.35957	−1123.07266	−90
E-**15b_t1**	−1123.78446	0.28744	−1123.49703	−1123.37118	−1123.08375	−119
**16b_t1**	−1123.75699	0.28722	−1123.46977	−1123.34765	−1123.05999	−57
**17b_t1**	−1123.80535	0.28171	−1123.52365	−1123.37546	−1123.09375	−145
E-**17b_t1**	−1123.80239	0.27881	−1123.52359	−1123.37212	−1123.09332	−144
**18b_t1**	−1123.79752	0.28241	−1123.51511	−1123.37199	−1123.08958	−134

[1] B3LYP = B3LYP(SMD,ACN)/6-311+G(2df,p)//B3LYP(SMD,ACN)/6-31G(d,p); M06 = M06-2X(SMD,ACN)/6-311++G(3df,2pd) //B3LYP(SMD,ACN)/6-31G(d,p). [2] E_{scf}, G_{corr}, and G_{tot} are given in a.u. ΔG_{rel} is given in kJ mol^{-1}. ΔG_{rel} is calculated using the equation: ΔG_{rel} (Structure) = G_{tot}(Structure) - [G_{tot}(**1b_t1**) + G_{tot}(**DMAD**)].

Table S2. (contd.)

Structure	E_{scf}(B3LYP)	G_{corr}	G_{tot} (B3LYP)	E_{scf}(M06)	G_{tot} (M06)	ΔG_{rel}(M06) [3]
**NCov(1b_t2)**	−1123.73168	0.27424	−1123.45744	−1123.29457	−1123.02033	47
**TS1(1b_t2)**	−1123.71135	0.27763	−1123.43372	−1123.27658	−1122.99896	103
**10b_t2**	−1123.74825	0.28251	−1123.46574	−1123.32337	−1123.04087	−7
**TS2(1b_t2)**	−1123.73591	0.28194	−1123.45397	−1123.30963	−1123.02769	28
**11b_t2**	−1123.74797	0.28155	−1123.46643	−1123.32281	−1123.04127	−8
**TS3(1b_t2)**	−1123.74341	0.27793	−1123.46548	−1123.31853	−1123.04060	−6
E-**12b_t2**	−1123.77978	0.28293	−1123.49685	−1123.35683	−1123.07390	−93
Z-**12b_t2**	−1123.78161	0.28416	−1123.49746	−1123.35837	−1123.07431	−94
**$TS4_{5C}$(1b_t2)**	−1123.76908	0.28465	−1123.48444	−1123.34549	−1123.06084	−59
**$TS4_{6C}$(1b_t2)**	−1123.75413	0.28758	−1123.46655	−1123.33802	−1123.05045	−32
**13b_t2**	−1123.77072	0.28467	−1123.48605	−1123.34874	−1123.06407	−68
E-**13b_t2**	**ring opening**					
**14b_t2**	−1123.75511	0.28576	−1123.46935	−1123.33925	−1123.05349	−40
**15b_t2**	−1123.77029	0.28685	−1123.48344	−1123.35657	−1123.06972	−82
E-**15b_t2**	−1123.78201	0.28909	−1123.49292	−1123.36947	−1123.08038	−110
**16b_t2**	−1123.77341	0.28716	−1123.48625	−1123.34868	−1123.06070	−59
**17b_t2**	−1123.80600	0.28003	−1123.52597	−1123.37515	−1123.09512	−149
E-**17b_t2**	−1123.80312	0.27947	−1123.52365	−1123.37220	−1123.09273	−143
**18b_t2**	−1123.79875	0.28183	−1123.51692	−1123.37245	−1123.09062	−137

[1] B3LYP = B3LYP(SMD,ACN)/6-311+G(2df,p)//B3LYP(SMD,ACN)/6-31G(d,p); M06 = M06-2X(SMD,ACN)/6-311++G(3df,2pd) //B3LYP(SMD,ACN)/6-31G(d,p). [2] E_{scf}, G_{corr}, and G_{tot} are given in a.u. ΔG_{rel} is given in kJ mol^{-1}. ΔG_{rel} is calculated using the equation: ΔG_{rel} (Structure) = G_{tot}(Structure) - [G_{tot}(**1b_t2**) + G_{tot}(**DMAD**)].

Table S2. (contd.)

Structure	E_{scf}(B3LYP)	G_{corr}	G_{tot} (B3LYP)	E_{scf}(M06)	G_{tot} (M06)	ΔG_{rel}(M06) [3]
**NCov(1c_t1)**	−1242.92411	0.36801	−1242.55610	−1242.42116	−1242.05315	30
**TS1(1c_t1)**	−1242.89782	0.37442	−1242.52340	−1242.40141	−1242.02700	99
**10c_t1**	−1242.92199	0.37979	−1242.54220	−1242.43725	−1242.05746	19
**TS2(1c_t1)**	−1242.91392	0.38058	−1242.53334	−1242.42491	−1242.04433	53
**11c_t1**	−1242.92932	0.38010	−1242.54923	−1242.44159	−1242.06149	8
**TS3(1c_t1)**	−1242.92108	0.37694	−1242.54415	−1242.43350	−1242.05657	21
E-**12c_t1**	−1242.96302	0.37984	−1242.58318	−1242.47682	−1242.09698	−85
Z-**12c_t1**	−1242.95167	0.38134	−1242.57033	−1242.47037	−1242.08903	−64
**TS4$_{5C}$(1c_t1)**	−1242.93757	0.38311	−1242.55447	−1242.45502	−1242.07192	−19
**TS4$_{6C}$(1c_t1)**	**ring opening**					
**13c_t1**	−1242.93968	0.38327	−1242.55641	−1242.45808	−1242.07481	−27
E-**13c_t1**	**ring opening**					
14c_t1 [4]	*−1242.92886*	*0.38357*	*−1242.54529*	*−1242.44173*	*−1242.05877*	*15*
**15c_t1**	−1242.94126	0.38305	−1242.55821	−1242.46473	−1242.08168	−45
E-**15c_t1**	−1242.95457	0.38457	−1242.57000	−1242.47803	−1242.09347	−76
**16c_t1**	−1242.92606	0.38447	−1242.54159	−1242.45422	−1242.06975	−14
**17c_t1**	−1242.98168	0.37785	−1242.60384	−1242.48582	−1242.10798	−114
E-**17c_t1**	−1242.97869	0.37785	−1242.60084	−1242.48509	−1242.10724	−112
**18c_t1**	−1242.96046	0.37813	−1242.58233	−1242.47065	−1242.09252	−73

[1] B3LYP = B3LYP(SMD,ACN)/6-311+G(2df,p)//B3LYP(SMD,ACN)/6-31G(d,p); M06 = M06-2X(SMD,ACN)/6-311++G(3df,2pd) //B3LYP(SMD,ACN)/6-31G(d,p). [2] E_{scf}, $G_{corr,}$ and G_{tot} are given in a.u. ΔG_{rel} is given in kJ mol^{-1}. ΔG_{rel} is calculated using the equation: ΔG_{rel} (Structure) = G_{tot}(Structure) - [G_{tot}(**1c_t1**) + G_{tot}(**DMAD**)]. [4] Partially-optimized point taken from the Scan calculations at the inflection point. Optimization of the TS structure did not converge.

Table S2. (contd.)

Structure	E_{scf}(B3LYP)	G_{corr}	G_{tot} (B3LYP)	E_{scf}(M06)	G_{tot} (M06)	ΔG_{rel}(M06) [3]
**NCov(1c_t2)**	−1242.92146	0.36859	−1242.55287	−1242.41772	−1242.04913	41
**TS1(1c_t2)**	−1242.89318	0.37533	−1242.51785	−1242.39876	−1242.02343	108
**10c_t2**	−1242.92400	0.38114	−1242.54286	−1242.43538	−1242.05424	27
**TS2(1c_t2)**	−1242.91212	0.37855	−1242.53357	−1242.41989	−1242.04133	61
**11c_t2**	−1242.92485	0.37923	−1242.54561	−1242.43537	−1242.05614	22
**TS3(1c_t2)**	−1242.91580	0.37622	−1242.53958	−1242.42618	−1242.04996	38
E-**12c_t2**	−1242.95221	0.37937	−1242.57284	−1242.46448	−1242.08512	−54
Z-**12c_t2**	−1242.95012	0.37936	−1242.57076	−1242.46582	−1242.08646	−57
**TS4$_{5C}$(1c_t2)**	−1242.94073	0.38303	−1242.55771	−1242.45631	−1242.07328	−23
**TS4$_{6C}$(1c_t2)**	**ring opening**					
**13c_t2**	−1242.94293	0.38300	−1242.55993	−1242.45960	−1242.07660	−32
E-**13c_t2**	**ring opening**					
**14c_t2**	**ring opening**					
**15c_t2**	−1242.94526	0.38208	−1242.56318	−1242.46893	−1242.08685	−58
E-**15c_t2**	−1242.95590	0.38296	−1242.57294	−1242.47842	−1242.09546	−81
**16c_t2**	−1242.92394	0.38368	−1242.54025	−1242.45352	−1242.06983	−14
**17c_t2**	−1242.97853	0.37646	−1242.60207	−1242.48200	−1242.10554	−107
E-**17c_t2**	−1242.97582	0.37731	−1242.59850	−1242.47947	−1242.10216	−99
**18c_t2**	−1242.96251	0.37897	−1242.58354	−1242.47340	−1242.09443	−78

[1] B3LYP = B3LYP(SMD,ACN)/6-311+G(2df,p)//B3LYP(SMD,ACN)/6-31G(d,p); M06 = M06-2X(SMD,ACN)/6-311++G(3df,2pd) //B3LYP(SMD,ACN)/6-31G(d,p). [2] E_{scf}, $G_{corr,}$ and G_{tot} are given in a.u. ΔG_{rel} is given in kJ mol^{-1}. ΔG_{rel} is calculated using the equation: ΔG_{rel} (Structure) = G_{tot}(Structure) - [G_{tot}(**1c_t2**) + G_{tot}(**DMAD**)].

S3. The reaction energy profiles calculated for **1a** and **1c**

Calculation method: M06-2X(SMD=ACN)/6-311++G(3df,2pd)//B3LYP(SMD=ACN)/6-31G(d,p)

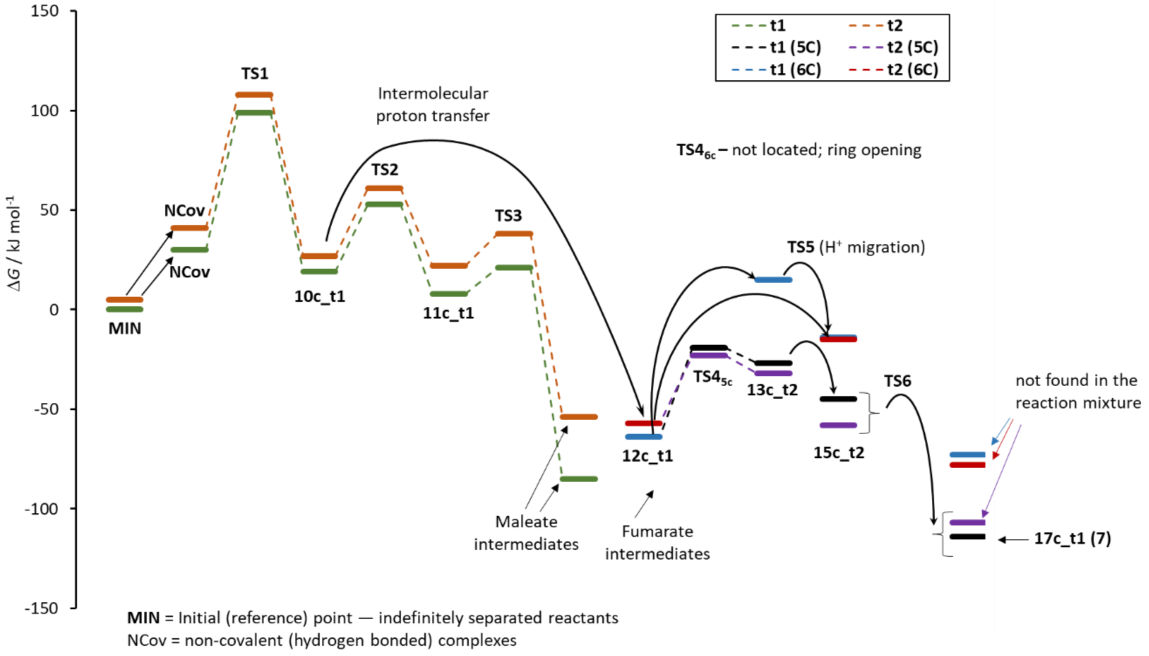

Figure S1. Energy profile for the AMA/CYC reaction of the tautomers of furfurylguanidine **1a**. Only the lowest energy points are labeled.

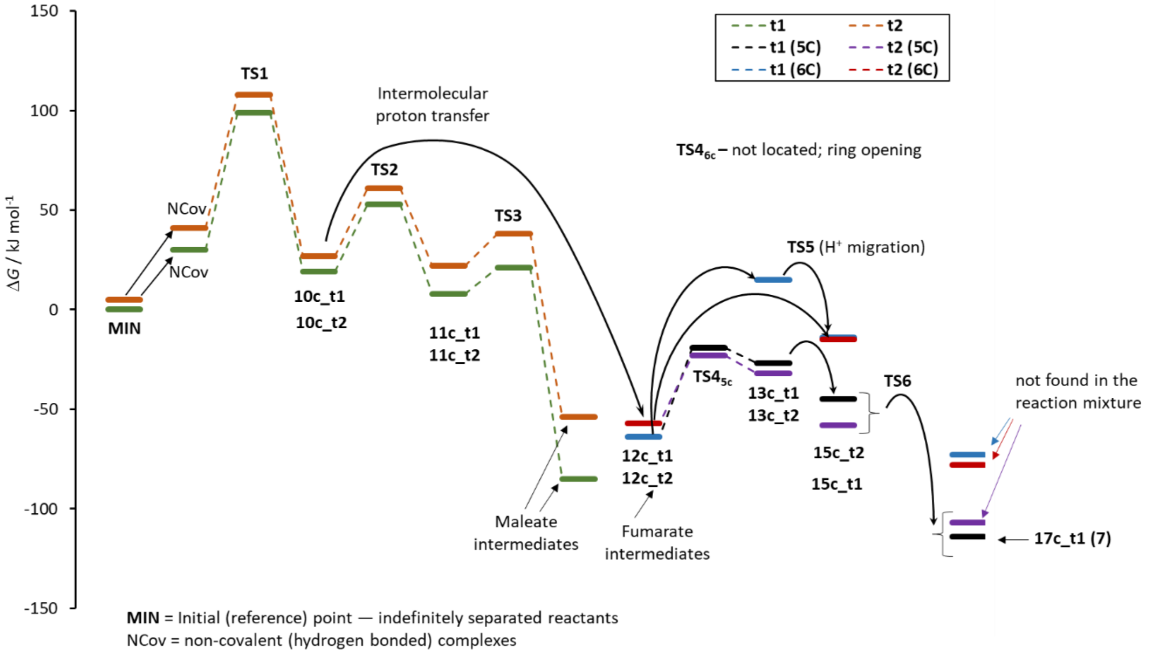

Figure S2. Energy profile for the AMA/CYC reaction of the tautomers of furfurylguanidine **1c**. Only the lowest energy points are labeled.

S4. The Entropy correction approach

- Gibbs energies of the stationary points along the reaction paths starting from **1b_t1** and **1b_t2** were corrected for the entropy contributions of the low-energy vibrations.
- The correction approach proposed by Cramer and Truhlar [1] was applied in which all vibrations below 100 cm^{-1} were shifted to 100 cm^{-1} and the vibrational entropy was recalculated.
- The uncorrected and corrected energy profiles were compared in Figures S3 and S4.
- The color coding was taken from the Figure 3 in the main text.

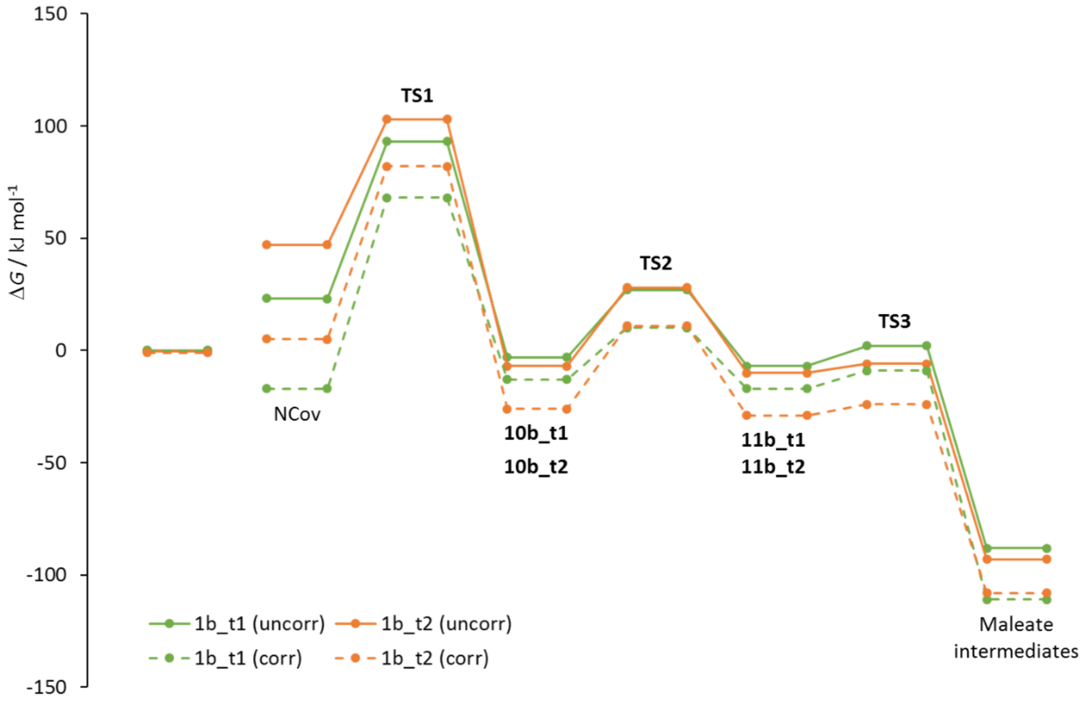

Figure S3. The first part of the uncorrected (solid lines) and corrected (dashed lines) reaction profiles starting from **1b_t1** (green) and **1b_t2** (yellow) tautomers.

Correction for the entropy contributions lowered the relative energies of covalently bonded structures by approximately 20-30 kJ mol^{-1} giving no change in the qualitative interpretation. The main difference is visible in the energetics of the non-covalent intermediates (**NCov**, Fig. S3) where the stabilization amounts more than 40 kJ mol^{-1}. This confirms an entropy as the main source of the unusually high energy of the **NCov** type of the intermediates. The barrier for the intramolecular cyclization step was reduced from -53 to -66 kJ mol^{-1} (33 and 36 kJ mol^{-1} above intermediates **12b_t1** for the uncorrected and corrected approach, respectively) and they are not considered as the reaction rate determining step. However, they do discriminate between the intramolecular cyclization to the smaller and larger ring. Both approaches predict the formation of the imidazolidinone system as the more favorable process.

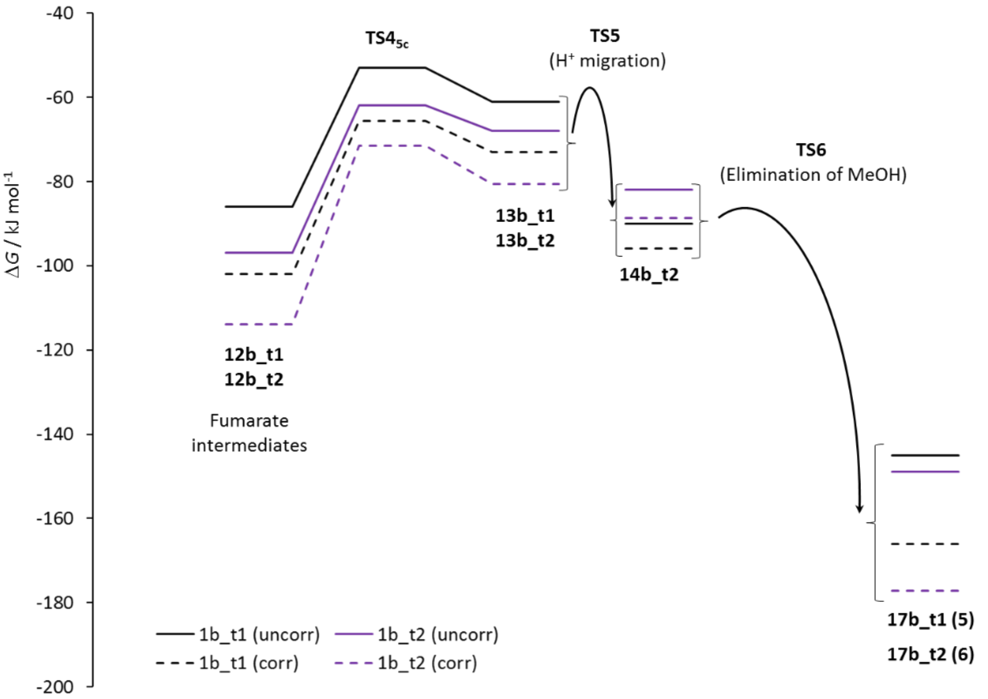

Figure S4. The second part of the uncorrected (solid lines) and corrected (dashed lines) reaction profiles starting from **1b_t1** (black) and **1b_t2** (purple) tautomers

The second important point is the difference in the relative energies of the products **17b_t1** and **17b_t2**. These structures correspond to the isolated products **5** and **6**, respectively, and their energy difference is compared with their relative abundance obtained experimentally (Figure 1, the main text). Entropy corrected approach predicts significantly larger difference in their relative energies (11 kJ mol^{-1}) with respect to the uncorrected approach (4 kJ mol^{-1}). Although both approaches favor the same isomer, the large difference obtained by entropy corrected approach would imply the absence of the NMR detectable amount of the other isomer. In this respect, the uncorrected data led to better agreement with the experiment. The described feature is consequence of the molecule of methanol hydrogen bonded to the product that remained for the sake of consistency with other structures along the considered paths. Therefore, we decided to present the uncorrected results since they provide trends in relative energies consistent with the entropy corrected ones and better agreement with the experimental results.

S5. Comparison of selected density functionals

Relative energies of all possible products (Scheme 1 in the main text) were calculated by five selected DFT methods. Besides B3LYP and M06-2X approach used for the reaction path calculations, The M08-HX in conjuntion with the Jensen's polarization consistent basis sets as well as M11 and wB97xD functionals with Dunning's augmented correlation consistent basis sets were employed. These three methods were recommended by Molteni and Ponti [2] and by Yepes et al. [3] as the very good methods for the kinetics and thermodynamics of the reactions.

B3LYP: B3LYP(SMD)/6-311+G(2df,p)//B3LYP(SMD)/6-31G(d,p)

M06-2X: M06-2X(SMD)/6-311++G(3df,2pd)//B3LYP(SMD)/6-31G(d,p)

M08-HX: M08-HX(SMD)/pcseg-3//M08-HX/pcseg-2

M11: M11(SMD)/aug-cc-pvtz//M11(SMD)/6-31+G(d,p)

wB97xD: wB97xD(SMD)/aug-cc-pvtz//wB97xD(SMD)/6-31+G(d,p)

In all cases the solvent of choice was acetonitrile and it was approximated as the dielectric continuum with the dielectric constant of 35.688 as implemented in Gaussian16.

The relative amount of the isomer *i* (Υ_i) were calculated using the equation (S1):

$$\Upsilon_i = e^{-\frac{\Delta G_{\mathrm{rel}}}{RT}} \qquad \text{(S1)}$$

where ΔG_{rel} is the Gibbs energy of the product *i* relative to the most stable product.

This is a variant of the approach used by Molteni and Ponti [2] except that the relative Gibbs energies of the final products instead of the relative enthalpies of the key TS structures were used.

Table S3. The product abundances (ϒ) relative to the most stable one calculated by different DFT approaches using the equation (S1).

**Ratios**	**Exp.**	**M08-HX**		**M06-2X**		M11		B3LYP		wB97xD	
	**ϒ**	**ΔG_{rel}**	**ϒ**	**ΔG_{rel}**	ϒ	ΔG_{rel}	ϒ	ΔG_{rel}	ϒ	ΔG_{rel}	ϒ
**17a_t1**	-	21.7	0.00	31.2	0.00	26.8	0.00	22.1	0.00	21.3	0.00
***E*–17a_t1**	-	33.3	0.00	41.9	0.00	35.1	0.00	29.6	0.00	28.3	0.00
**17a_t2**	1.00	4.3	**0.18**	14.0	0.00	15.9	0.00	2.0	0.45	12.8	0.01
***E*–17a_t2**	-	25.9	0.00	32.3	0.00	38.1	0.00	17.5	0.00	30.2	0.00
**18a_t1**	0.33	0.9	**0.69**	8.8	0.03	4.0	0.20	11.1	0.01	0.0	1.00
**18a_t2**	0.39	0.0	**1.00**	0.0	1.00	0.0	1.00	0.0	1.00	4.6	0.16
**17b_t1**	0.50	2.1	**0.44**	4.7	0.15	0.0	1.00	10.5	0.01	0.1	0.96
***E*–17b_t1**	-	10.2	0.02	11.5	0.01	8.8	0.03	15.1	0.00	8.4	0.03
**17b_t2**	1.00	0.0	**1.00**	0.0	1.00	0.5	0.83	0.0	1.00	0.0	1.00
***E*–17b_t2**	-	8.5	0.03	12.7	0.01	9.1	0.03	14.0	0.00	7.6	0.05
**18b_t1**	-	14.6	0.00	15.2	0.00	11.7	0.01	31.3	0.00	17.9	0.00
**18b_t2**	-	18.1	0.00	18.9	0.00	14.8	0.00	36.6	0.00	22.2	0.00
**17c_t1**	1.00	0.0	**1.00**	0.0	1.00	0.1	0.94	0.0	1.00	0.3	0.90
***E*–17c_t1**	0.23	2.7	**0.34**	1.7	0.51	0.0	1.00	4.9	0.14	0.0	1.00
**17c_t2**	-	9.0	0.03	12.2	0.01	8.2	0.04	8.1	0.04	4.8	0.15
***E*–17c_t2**	-	11.6	0.01	12.1	0.01	14.2	0.00	11.1	0.01	9.0	0.03
**18c_t1**	-	48.3	0.00	45.7	0.00	45.3	0.00	62.3	0.00	52.3	0.00
**18c_t2**	-	43.5	0.00	40.9	0.00	38.0	0.00	59.3	0.00	43.4	0.00

S6. The methanol elimination catalyzed with guanidine

The steps within the suggested mechanism that are assumed to occur due to intermolecular interactions are modeled using guanidine **6rMe** as the catalyst and / or a supporting molecule for the proton shift.

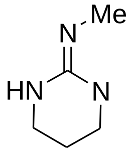

6rMe

Figure S5. Structure of the guanidine catalyst **6rMe** representing the simplified **1b_t1** structure.

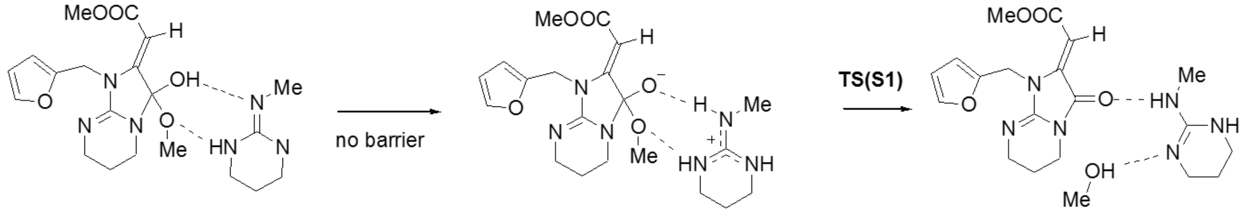

15b_t1 **19** **20**

ΔG_{rel}(**15b_t1**) = -97 kJ mol^{-1}

ΔG_{rel}(**19**) = -97 kJ mol^{-1}

ΔG_{rel}(**TS(S1)**) = -72 kJ mol^{-1}

ΔG_{rel}(**20**) = -134 kJ mol^{-1}

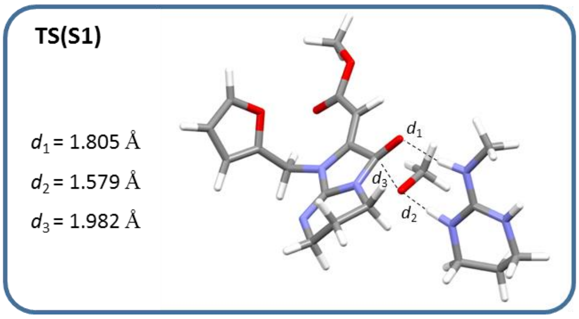

Figure S6. Calculated relative Gibbs energies (ΔG_{rel}) for the methanol elimination step catalyzed with guanidine. The values are calculated relative to the sum of the Gibbs energies of the guanidine **1b_t1**, **DMAD**, and guanidine catalyst **6rMe**.

S7. Cartesian coordinates

S7.1. G3 Optimized geometries

1aH⁺

C 0.240948 0.079294 0.095551
N 0.539287 -0.775482 1.069933
C 1.691474 -0.301009 1.835088
C 2.319614 0.693091 0.850583
N 1.179241 1.008427 -0.010802
H -0.161131 -1.363768 1.471251
H 2.352255 -1.117766 2.07947
H 1.366003 0.185402 2.745633
H 3.11297 0.239966 0.270598
H 2.689517 1.583472 1.333902
H 1.267658 1.597262 -0.811101
N -0.838966 -0.01549 -0.649608
C -1.237514 0.934633 -1.689527
C -2.204148 0.298663 -2.626612
H -1.698941 1.806636 -1.246366
H -0.357954 1.2472 -2.235631
C -3.502415 0.521682 -2.875804
C -3.874539 -0.411296 -3.909713
C -2.764774 -1.109928 -4.182114
O -1.739833 -0.693228 -3.415159
H -2.551639 -1.903444 -4.866162
H -4.834959 -0.528763 -4.368235
H -4.125434 1.249555 -2.397122
H -1.515373 -0.696529 -0.368689

1a_t1

C 0.2608827016 0.0087151 0.0148626769
N 0.5238908068 -0.6899281043 1.1692605503
C 1.4359817557 0.0697103675 2.0110698943
C 2.1905759698 0.8838675528 0.9613506762
N 1.166188066 1.0357365116 -0.0635825119
H -0.2563416204 -1.1433421307 1.5956724637
H 2.0845170285 -0.5817886694 2.579576002
H 0.9022510115 0.7201418547 2.6979636241
H 3.0497022119 0.3372464552 0.5852134001
H 2.5206611301 1.8438440751 1.3322867752
H 1.4437221827 1.3502698813 -0.9690395245
N -0.6784904255 -0.2912129713 -0.7837502927
C -0.829134663 0.5223865551 -1.9671088979
C -2.0571060985 0.1122591541 -2.7174887201
H -0.9135182195 1.5841731777 -1.7331912756
H 0.0226587475 0.4322208459 -2.6427888167
C -3.0136544992 -0.807847801 -2.5173689761
C -3.9265134073 -0.682779829 -3.6301538681

C -3.4367646474 0.2983968533 -4.3957838966
O -2.3014302085 0.7928299764 -3.8564402763
H -3.7589271619 0.7448048055 -5.3123338391
H -4.8144806869 -1.253676568 -3.8128216976
H -3.0781739736 -1.4910590923 -1.6969584697

1a_t2
C 0.2690074093 0.1504381254 0.1251336102
N 0.170758332 -0.0568173271 1.4882380095
C 1.3106969397 0.6424850221 2.0771228312
C 2.2505316742 0.7407672217 0.8608623302
N 1.3877685517 0.5726152523 -0.3074641278
H -0.7257621672 0.1199024082 1.8943315949
H 1.7344514167 0.084901831 2.9015531002
H 1.0197640639 1.624917576 2.4367425044
H 3.0013067564 -0.0454962608 0.8845738311
H 2.7774852921 1.6873682268 0.8270036437
N -0.8281684481 -0.108655506 -0.6366423696
C -0.7063911893 -0.1904765877 -2.0824176756
C -2.0508113953 -0.2333042369 -2.7274166965
H -0.1647086725 0.6804614911 -2.4245496205
H -0.1505997792 -1.0688688172 -2.393542841
H -1.4736527537 -0.7536825011 -0.2298903168
C -2.6777032638 -1.1719952786 -3.4530881484
C -3.9550953421 -0.6106397278 -3.81802307
C -3.9783314911 0.6134511234 -3.2759864398
O -2.8327904211 0.8608279017 -2.6123369135
H -4.7063027782 1.3965537219 -3.2718321418
H -4.72910089 -1.0690378587 -4.3991257775
H -2.2950048444 -2.1404287996 -3.7046023165

1bH⁺
C -0.0635268286 1.063706493 -0.1261343825
N -0.2143293604 2.0040809079 0.7891350673
N 1.0862015624 0.9176435085 -0.7793203841
C -1.4498963231 2.2169829854 1.5456090675
H 0.5125778103 2.6755156092 0.9054254478
C -2.2283321298 0.9150411338 1.6327857116
H -1.1690190909 2.5714781079 2.5264599259
H -2.0389621431 2.9851368307 1.0592085987
C -2.3740645773 0.3142481978 0.2444284089
H -1.7083132742 0.2175452924 2.2805324251
H -3.2067100032 1.105984745 2.0554008911
N -1.0568130872 0.2264892128 -0.382391871
H -2.7752614784 -0.6872991932 0.2911123176
H -3.0319676902 0.9185472038 -0.3692179186
H -0.9036108109 -0.4780494903 -1.0726384457
C 2.2667867589 1.7661728268 -0.6278839173
H 2.1047382698 2.7371381643 -1.0779637805
H 2.4723411757 1.9030919659 0.42616609

C 3.4419765691 1.1251657261 -1.2805370359
C 4.1746551863 1.4548100797 -2.3537716488
C 5.1902688884 0.4383015276 -2.4693052613
C 4.9700164267 -0.4092458915 -1.4559686215
O 3.9145686735 -0.0090954749 -0.7220972473
H 5.4557014092 -1.3063347321 -1.1360224989
H 5.9617158205 0.3672810169 -3.208426889
H 4.0253773776 2.3044986085 -2.988448748
H 1.0833588684 0.2773496376 -1.5456703009

1b_t1
C 0.0563616708 0.8727678337 0.0289373934
N -0.1367422221 1.9118033237 0.8910014106
N 1.1292724993 0.6390238666 -0.6271904361
C -1.4485282871 2.3376443382 1.3654210562
H 0.5424235338 2.6381478312 0.8416492765
C -2.3536530837 1.1313083454 1.5465590509
H -1.3074254372 2.8610020958 2.3022964083
H -1.900292424 3.0338968733 0.663078398
C -2.3436890775 0.310967941 0.2680828253
H -1.9983584321 0.5226506391 2.3722339115
H -3.3609260623 1.4597578707 1.7762100921
N -0.9697730282 -0.0323959111 -0.0575844754
H -2.894954435 -0.6118201195 0.3949831713
H -2.818219786 0.8712187113 -0.5357519927
H -0.8437082656 -0.6783713955 -0.8070202215
C 2.25079033 1.5390534399 -0.4590356269
H 2.031587097 2.5515623578 -0.7955560577
H 2.5584311339 1.6193540051 0.5845387965
C 3.4281571885 1.0683680449 -1.2508916529
C 4.096369515 1.5936816158 -2.2903517963
C 5.1585206472 0.6662274981 -2.5984116504
C 5.0327948643 -0.3308729649 -1.7145788702
O 3.9918186668 -0.1026947049 -0.8886831012
H 5.5797788127 -1.2346670154 -1.5493068703
H 5.8967056827 0.7487630124 -3.3702484604
H 3.8743638989 2.5171804675 -2.786102579

1b_t2
C -0.8959972992 -0.0052116743 -0.3674442102
N -1.4962866005 0.1609243595 0.7389442035
C -1.1229426469 1.2954887945 1.5703086698
N 0.0175642726 0.8524938874 -0.9309884048
H 0.6400717452 0.4309949632 -1.5879765324
N -1.1405077178 -1.116425785 -1.1463735282
C -2.2655895335 -1.9806085937 -0.8365210313
C -2.2597868209 -3.1866483043 -1.7160129156
H -2.1887211697 -2.2857227382 0.1973479426
H -3.2180734847 -1.4746019729 -0.9585800454
O -1.2515322933 -4.0702219739 -1.5568446371

C -1.4503954214 -5.0770143705 -2.4292635115
C -2.5617063544 -4.8675580191 -3.1459885592
C -3.0970474417 -3.6141274416 -2.6739282407
H -3.9836275137 -3.1157823482 -3.0103305291
H -2.9614532302 -5.5036968418 -3.9092278745
H -0.7235771876 -5.8611677899 -2.4209284797
H -2.0130415435 1.6403767195 2.0872804189
H -0.4326842102 0.9624886153 2.3441175115
C -0.4944122184 2.4507458764 0.7959007001
C 0.5952206086 1.9060128699 -0.1128829648
H -0.0819389994 3.1872288389 1.4776629268
H -1.2502392069 2.9447339731 0.1912463784
H -1.0161393491 -0.9552843756 -2.1250508428
H 0.982523256 2.6742685019 -0.7705188794
H 1.4245223606 1.5301778291 0.4828444351

1cH⁺

C 0.1455778506 0.4814245667 0.9232843496
N 0.6426521833 0.260374922 2.130692018
N 0.9560799872 0.8401061104 -0.0705857116
N -1.1559456002 0.364581104 0.6976572543
H 0.0402463456 -0.1840098514 2.7896549604
C 2.0081060785 0.5353641224 2.6065278875
H -1.4408731894 0.307495424 -0.2564837146
C -2.2243555324 0.1553613286 1.6867810245
H -1.8948215934 0.5933166476 2.6183716436
C -3.4582348021 0.914080073 1.2107900074
C -2.5096558617 -1.3313218703 1.8861623506
C 1.916333676 1.0081682567 4.0530932537
C 2.8977127333 -0.6965223951 2.4628465641
H 2.4056127033 1.3444198576 2.0098274386
C 0.5666053933 1.3875336902 -1.3657966962
C 0.463405125 0.3598251154 -2.4440168704
H 1.3222167692 2.1056052374 -1.6467318118
H -0.3658772844 1.9245737487 -1.256813879
O -0.5164619034 -0.5622059715 -2.32045398
C -0.4431956698 -1.3788038791 -3.3926323392
C 0.5542515473 -1.001383225 -4.2009723303
C 1.1558828956 0.1493131599 -3.5718143753
H 1.9865939486 0.7258580949 -3.9249829601
H 0.8414791469 -1.4654808721 -5.1222224255
H -1.1603986256 -2.1701924043 -3.4343004313
H 1.9347065414 0.7187658041 0.0722582052
H -3.2911574273 -1.4638519705 2.6271281975
H -2.8401115199 -1.7825383483 0.9554280455
H -1.6264944921 -1.8619155813 2.2267041912
H -4.2632514282 0.7957016103 1.9274406949
H -3.244559927 1.9721162917 1.1045924947
H -3.8004395261 0.5330778922 0.2526450101
H 2.9072820441 1.2363807404 4.4293104796

H 1.3047557981 1.9005541914 4.1305701845
H 1.4844238651 0.2373362633 4.6851300789
H 3.9007344525 -0.4734978698 2.8115894151
H 2.5053946297 -1.5196634488 3.0523765427
H 2.9627806684 -1.0188395653 1.4285342335

1c_t1
C 1.3002778968 0.2686462433 0.9113701204
N 2.5838195682 0.660842059 0.5645107507
N 1.0793000687 -0.9641170965 1.2599466662
N 0.3397381908 1.2655317393 0.8437319688
C 0.6350424441 2.7033146078 0.9146308623
H -0.5087573427 1.0345657373 1.3550774655
H 2.6207358044 1.4256365991 -0.1055488322
C 3.6169327516 -0.3471940288 0.3208844017
C -0.2736144635 -1.2821155915 1.7060950472
C 1.2064920759 3.1143812553 2.2662119709
C -0.6547471467 3.4469266364 0.6025316666
H 1.3579834602 2.9432121294 0.1283821428
H 3.5592706908 -1.0528184081 1.1537052467
C 3.3974028367 -1.1035148249 -0.984098094
C 4.9666154525 0.3536831669 0.3434885284
H -1.0202011071 -1.0720544321 0.9223607866
H -0.5781648896 -0.7027610973 2.5931137603
C -0.3598315473 -2.717533343 2.076026415
H 4.1797190828 -1.8555121627 -1.1319275111
H 2.4295176674 -1.6093093941 -0.9721280247
H 3.425517964 -0.4088945271 -1.8309079977
H 5.7757811923 -0.3682862955 0.1988129434
H 5.0257572918 1.0945954794 -0.4618265565
H 5.1222271865 0.8668579785 1.2965555933
H -0.4817484149 4.5266897375 0.6080950593
H -1.0417764191 3.158720166 -0.3785234356
H -1.4156972742 3.2200359215 1.357573388
H 1.4174550348 4.1886696203 2.2831713882
H 0.4842918314 2.8913642457 3.0587642597
H 2.1345079603 2.5769079524 2.474260861
C -0.5064014601 -3.3856175978 3.2615447257
C -0.4970934886 -4.779644484 2.954713501
C -0.3434073131 -4.8693868051 1.6012392065
O -0.2597707004 -3.6219364881 1.0495326762
H -0.2798355812 -5.6962878285 0.9077441728
H -0.5908891385 -5.6085596162 3.6440372356
H -0.6074261648 -2.9272122537 4.237075641

1c_t2
C -0.8493651505 0.0296617036 -0.4755077332
N -1.4173967759 0.1445053501 0.683188617
C -1.0363311814 1.2496630486 1.5594842097
N 0.0103004258 0.93708443 -1.0826912611

C 1.1037968169 0.5128860623 -1.97242643
N -1.1098861766 -1.0978468529 -1.2506358295
C -2.1616635138 -2.0019589569 -0.7931612411
C -2.2390548129 -3.1787782593 -1.6927555032
H -1.921634921 -2.3145786924 0.226482149
H -3.1492770002 -1.5210948853 -0.7579431415
O -1.1914508824 -4.063193456 -1.649021462
C -1.4630007933 -5.0518508669 -2.5521779707
C -2.6584702993 -4.8171079094 -3.1683161674
C -3.1600644506 -3.6023568518 -2.613406689
H -4.0868028915 -3.0990755228 -2.8580129047
H -3.1203515266 -5.4379097872 -3.9247138497
H -0.7199983373 -5.8330243847 -2.631777513
C -1.2687612065 0.8071174347 2.9972071212
H 0.0336540308 1.4989604749 1.4580400551
C -1.8568696797 2.4935114047 1.2290477537
H 0.3197941094 1.6645873303 -0.4415991357
C 1.715013975 1.7689793055 -2.5736219071
C 2.1466321546 -0.3287254073 -1.2476094115
H 0.6675070875 -0.0775600204 -2.7831637291
H 2.9449662135 -0.6283205543 -1.9346896807
H 2.5927410873 0.2494480587 -0.4312511009
H 1.6943835995 -1.2329842802 -0.8323886528
H -0.9905365755 1.5975847482 3.702028208
H -2.3256394434 0.5634624581 3.145505605
H -0.6768967296 -0.0843765532 3.2250353453
H -1.6202168444 3.3149634772 1.9136422398
H -1.6650567667 2.8327046917 0.2066858003
H -2.9230448103 2.2620358699 1.3193841095
H -1.1750645107 -0.900667765 -2.2473790402
H 2.5135262922 1.5071914121 -3.2735631465
H 0.9589477426 2.3518584634 -3.1066437916
H 2.1458787451 2.3965292823 -1.7854429217

9H
C -0.25033607 0.2469728768 -0.0682378327
O -0.0879080996 0.0199175077 1.2044321856
O 0.6098658934 0.6048621792 -0.8107262294
C 1.219197842 0.2152185245 1.747394019
C -1.6647266216 0.0124187944 -0.5439916635
H 1.1342417831 -0.0232833044 2.7949185209
H 1.9235384055 -0.4466736718 1.2664489254
H 1.5247945338 1.2426241657 1.6187323895
C -1.9290223098 0.2168621039 -1.8223593571
C -3.2351045936 0.0548646446 -2.5233679005
O -3.089216152 0.3549315967 -3.7958336145
O -4.2625170858 -0.2891939771 -2.0312043526
C -4.2397374224 0.2584397588 -4.6309068509
H -3.9086154327 0.5423232593 -5.6168895336
H -4.612823385 -0.7552522454 -4.639830199

H -5.0103224164 0.9329402623 -4.2872601588
Cl -2.8150467336 -0.5056524046 0.636991996
H -1.1130271351 0.5380469296 -2.4384543437

DMAD
C 0.0717134319 0.0295736226 -0.2122909237
O -0.0404939993 -0.0291039367 1.0862694224
O 1.0853985399 0.0392649684 -0.831708356
C 1.1694731379 -0.0873043413 1.8493161106
C -1.2408324254 0.0838802585 -0.856448544
H 0.8558111084 -0.1353361902 2.8789255976
H 1.7320101712 -0.9697625444 1.5844524664
H 1.7596918847 0.7998659093 1.6758817414
C -2.2887206424 0.1254068029 -1.4104633133
C -3.58123962 0.2204931764 -2.089450081
O -4.0779973878 -0.9619112238 -2.3291137149
O -4.0787092372 1.2628231649 -2.3676575893
C -5.3455360024 -1.0210925964 -2.9920514351
H -5.5704053433 -2.0706344295 -3.0852329064
H -6.0977408417 -0.5251541914 -2.3974369306
H -5.2753887745 -0.5624474492 -3.9668505438

S7.2. B3LYP Optimized geometries (from 1a tautomers)

1a_t1
C 0.272352 -0.009719 0.01303
N 0.550169 -0.717592 1.175585
C 1.441815 0.071384 2.029531
C 2.191558 0.907206 0.981699
N 1.163125 1.053039 -0.054921
H -0.235466 -1.19078 1.604889
H 2.11082 -0.56596 2.612493
H 0.883223 0.719253 2.71941
H 3.071179 0.368603 0.60484
H 2.512619 1.877019 1.369083
H 1.455923 1.359399 -0.974573
N -0.671349 -0.331952 -0.803207
C -0.815241 0.499977 -1.986651
C -2.052696 0.112524 -2.735445
H -0.873466 1.575697 -1.740786
H 0.04736 0.408287 -2.671776
C -3.040568 -0.803237 -2.524573
C -3.958577 -0.675425 -3.624538
C -3.46218 0.306994 -4.421993
O -2.297784 0.799822 -3.894635
H -3.788266 0.75578 -5.348479
H -4.864249 -1.242188 -3.792412
H -3.103805 -1.483163 -1.688126

1a_t2

C 0.276957 0.103401 0.1375
N 0.212029 -0.117316 1.516392
C 1.314908 0.6736 2.090454
C 2.236377 0.836415 0.855237
N 1.376559 0.609615 -0.321674
H -0.701115 0.011227 1.940857
H 1.795264 0.148144 2.920425
H 0.957082 1.645426 2.456085
H 3.048299 0.094522 0.872494
H 2.707721 1.824847 0.8213
N -0.817424 -0.228571 -0.618672
C -0.708883 -0.287775 -2.076931
C -2.047442 -0.262266 -2.731584
H -0.11138 0.572966 -2.391502
H -0.181549 -1.189406 -2.416685
H -1.437205 -0.914183 -0.203796
C -2.736851 -1.176974 -3.473198
C -3.977134 -0.556471 -3.844132
C -3.952104 0.689463 -3.29791
O -2.784969 0.888636 -2.617733
H -4.6468 1.516413 -3.297537
H -4.775179 -0.983347 -4.435451
H -2.399814 -2.173079 -3.725296

NCov(1a_t1)

C 1.0838098504 0.4074396551 2.0668025817
N 1.6672426181 0.1903451158 3.3111579633
C 2.8977242507 0.9825497445 3.4251686561
C 2.6076086083 2.1302584567 2.4471572431
N 1.7631833004 1.4548439369 1.4604518119
H 1.6905155128 -0.761563726 3.6545320878
H 3.7779795988 0.4081312044 3.1069447265
H 3.0580245598 1.3318986319 4.4480394388
H 3.5166356341 2.5334109581 1.9941107381
H 2.0758370056 2.9487325295 2.9531205817
H 1.2255350792 2.0127641505 0.8049370388
N 0.0850021586 -0.198401395 1.5186450046
C -0.5075165965 -1.2991379669 2.2797812943
C -1.8801309557 -1.6315046869 1.7923364697
H 0.0907756554 -2.2193324068 2.1956366747
H -0.5689055016 -1.0698745676 3.3570927637
O -2.8746382945 -0.7250519346 2.0629695873
C -4.0337862729 -1.2207596016 1.533857378
C -3.8051610426 -2.4221908265 0.9381147977
C -2.4036803235 -2.6885975337 1.1068388067
H -1.8547879109 -3.5540335157 0.7615459759
H -4.5382419049 -3.0431323548 0.4418493956
H -4.915892626 -0.6132178115 1.6715922013
C 0.9534576891 0.084766917 -2.7130042903

C 0.1818036055 0.7195379679 -2.0288004378
C 1.8868620609 -0.742049209 -3.4563753151
C -0.679741119 1.5510497032 -1.2099387079
O 2.0263923741 -0.3018182669 -4.7091273214
O 2.4501096834 -1.7024378721 -2.9699768834
C 2.941822905 -1.0577672061 -5.5413545786
O -1.9379189086 1.1229500591 -1.2389817026
O -0.273369386 2.5284119122 -0.6051382919
C -2.8702459303 1.8534281486 -0.4012866557
H -2.5645644959 1.7762671929 0.6431176867
H -2.9128638639 2.9005797291 -0.7071395841
H -3.8323969615 1.3688966087 -0.5578100977
H 2.9231135548 -0.5614895603 -6.5104009147
H 3.9475513338 -1.031784408 -5.1166755264
H 2.6033670554 -2.091840772 -5.6333115963

NCov(1a_t2)
C 0.4974175568 -1.125426568 0.810625074
N 0.7237104639 -2.1295917612 1.7591915778
C 2.1319801692 -2.5265155677 1.5819140104
C 2.7063152027 -1.2860433225 0.853486004
N 1.5481569153 -0.5986744069 0.2588465225
H 0.0513352141 -2.8899898258 1.7643007541
H 2.2095073771 -3.4295443147 0.96178645
H 2.61588833 -2.7258903509 2.54215282
H 3.4450232299 -1.5625955111 0.0922804775
H 3.2157921508 -0.61849432 1.5651504295
N -0.7840771456 -0.743148826 0.5392358239
H -0.8953921047 0.0551357546 -0.0759442149
C -1.9467996382 -1.120365202 1.3409671349
C -2.6614595547 -2.3434292511 0.8571211926
H -1.6434829986 -1.2579674491 2.3855397029
H -2.655245858 -0.2894315791 1.3180750479
O -2.0090939173 -3.5420837449 1.0195555344
C -2.8186556811 -4.5208234773 0.5093715649
C -3.9696733748 -3.9753547801 0.0340736116
C -3.866895159 -2.5600814055 0.2588965863
H -4.5954036528 -1.8015734856 0.0075933705
H -4.7917230235 -4.5100161995 -0.421185013
H -2.4349899521 -5.5286515806 0.5635711881
C 1.3795906825 2.8043329867 -0.5199838579
C 0.2080640027 2.8565794395 -0.8207978871
C 2.7571111516 2.7344316721 -0.0658290029
C -1.1966786118 2.7577062711 -1.1549893308
O 3.5838769315 2.4701416517 -1.079185057
O 3.077936962 2.9058688588 1.0932850427
C 4.988270233 2.3705544672 -0.733677373
O -1.6816134633 3.9027093707 -1.624949589
O -1.8308738269 1.7244031632 -1.011593654
C -3.0878413125 3.8969141946 -1.9856277959

H 5.499573521 2.1653781333 -1.6729346351
H 5.3393352572 3.3103738267 -0.3026601429
H 5.1457362392 1.5544210998 -0.0254296473
H -3.2934561829 4.9046454396 -2.3426132197
H -3.2708609688 3.1642647406 -2.7739652073
H -3.6990231638 3.6662158594 -1.1108682928

TS1(1a_t1)
C -0.4993518012 0.0086627884 -0.2499200529
N -0.0479819636 -0.2960267642 1.0210980303
C 0.8948592749 0.7355952549 1.4737008795
C 0.4513349547 1.9397533278 0.6322063156
N -0.0778656858 1.2811332132 -0.566591274
H 0.1763243596 -1.2616306248 1.224408606
H 1.9305702577 0.4526774186 1.2463778264
H 0.8057610152 0.9155604681 2.5472555527
H 1.2798768219 2.6087220164 0.3889675026
H -0.3264518193 2.5171862246 1.1498889302
H -0.6418204334 1.8330774001 -1.2096089065
N -1.2144237287 -0.7386518315 -1.0356396305
C -1.5599675005 -2.0911613422 -0.6057575567
C -2.8057569332 -2.5836054659 -1.2665256732
H -0.7586341467 -2.7991013928 -0.8499331133
H -1.708697499 -2.1277258211 0.4844273053
O -3.9762258338 -1.9460699747 -0.9446297298
C -4.9795647279 -2.5648110349 -1.6351474429
C -4.4799187055 -3.5838843491 -2.3851878974
C -3.0639332588 -3.5954472616 -2.1454590048
H -2.3317164677 -4.267601385 -2.5709699587
H -5.0435769733 -4.2439928594 -3.0298515342
H -5.9722448327 -2.1659960117 -1.4863004259
C -1.3937933792 -0.1669754848 -3.1482180542
C -2.0039181803 0.8945535031 -3.367624295
C -0.6564255603 -1.2929777294 -3.7418566721
C -2.5873523194 2.1034113337 -2.8779221019
O -1.0002708644 -1.4068478671 -5.0391158802
O 0.1576670981 -2.0060114048 -3.1919238154
C -0.3083258981 -2.4398797874 -5.7744951295
O -3.9005479047 2.1956362935 -3.1449879606
O -1.9457299623 2.9749721905 -2.2920513495
C -4.5624167388 3.3776889735 -2.6438139794
H -4.4830402782 3.4323872349 -1.5554233591
H -4.1308822573 4.2776982768 -3.087925967
H -5.6053676919 3.2724801413 -2.9415169266
H -0.7040338838 -2.3857628423 -6.7883677346
H 0.7685980598 -2.2555768453 -5.7763154919
H -0.5111366121 -3.4213109792 -5.339397031

TS1(1a_t2)
C 0.0812954888 0.1558044619 -0.6576379496

N -0.1349458845 -0.4534805767 0.5666811136
C 1.1878314405 -0.5907194628 1.2020584684
C 2.005692204 0.4779516826 0.4425531785
N 1.2742962238 0.6748126891 -0.8225471645
H -0.7318029364 -1.274284383 0.5709366175
H 1.5930246088 -1.5973583396 1.0393917846
H 1.1326069084 -0.4057560513 2.2775781775
H 3.0352720206 0.1677358042 0.2588731204
H 2.0393460712 1.4198590826 1.0067163791
N -0.9113198778 0.2203470146 -1.5737310496
H -0.7213570937 0.7429456178 -2.4171527112
C -2.3078753334 -0.1387440935 -1.3195781609
C -2.6475746754 -1.5582296246 -1.6410507583
H -2.5485072137 0.0765364523 -0.2723519866
H -2.9275324352 0.5133951608 -1.9376641962
O -2.1407654442 -2.5157675333 -0.795338766
C -2.5536839488 -3.7332927626 -1.2644225927
C -3.3134326568 -3.5721242955 -2.3804364445
C -3.3734263472 -2.1581852792 -2.6260742193
H -3.8893614559 -1.6533321663 -3.4310475553
H -3.776275592 -4.3591748183 -2.9593889708
H -2.2289288474 -4.5938962678 -0.6989375144
C 1.4076885549 2.4610853151 -2.0124473201
C 0.3639895724 3.0960153163 -2.2437826287
C 2.8592773297 2.4410895168 -2.2501839298
C -1.0253506066 3.3978832013 -2.3211154019
O 3.1333609302 3.1213748988 -3.3812636707
O 3.7086689787 1.9306911991 -1.5490772004
C 4.5347491525 3.2315744735 -3.7117060124
O -1.3850967264 4.385115578 -1.4739770753
O -1.801340559 2.8443182601 -3.0967255846
C -2.7782582007 4.7578987623 -1.5063851049
H 4.5684767247 3.8010935674 -4.6402422137
H 5.0762825303 3.7592513988 -2.9229468685
H 4.9746375226 2.2420408572 -3.8571232666
H -2.8799792664 5.5614397138 -0.7769776387
H -3.0618300866 5.1126500136 -2.5002517342
H -3.4129120741 3.9132856164 -1.2266521491

10a_t1

C 1.296988 0.567029 1.441002
N 1.627521 0.287599 2.724018
C 2.595526 1.283152 3.216177
C 2.380847 2.440835 2.227482
N 1.802361 1.757015 1.056775
H 1.710827 -0.673153 3.031663
H 3.612609 0.881454 3.156079
H 2.381548 1.561874 4.249127
H 3.311128 2.948202 1.967004
H 1.671064 3.178563 2.618272

H	1.267215	2.323688	0.380646
N	0.545860	-0.223768	0.668740
C	0.006750	-1.487321	1.239088
C	-1.231054	-1.942849	0.552429
H	0.757487	-2.277805	1.167987
H	-0.216579	-1.303690	2.294112
O	-2.370675	-1.213063	0.767041
C	-3.374296	-1.821628	0.071863
C	-2.905786	-2.925667	-0.572088
C	-1.508119	-3.004113	-0.259474
H	-0.799253	-3.748085	-0.595060
H	-3.479285	-3.599611	-1.192931
H	-4.346228	-1.355536	0.138544
C	0.479606	0.004223	-0.792061
C	-0.135638	1.020976	-1.396254
C	1.258040	-1.041774	-1.516524
C	-0.763216	2.152626	-0.822238
O	1.101886	-0.987878	-2.853979
O	1.973158	-1.873496	-0.972873
C	1.853464	-1.958883	-3.602846
O	-2.123467	2.141669	-0.898052
O	-0.150247	3.159967	-0.406189
C	-2.785342	3.333559	-0.448164
H	-2.558085	3.545792	0.600597
H	-2.502463	4.198910	-1.054611
H	-3.852769	3.137069	-0.561680
H	1.611980	-1.771785	-4.649686
H	2.927173	-1.835628	-3.437479
H	1.562779	-2.975997	-3.326508

10a_t2

C	0.3539824618	-0.5419705628	0.8141834355
N	0.4746045944	-1.6342897088	1.5992758593
C	1.7591836386	-2.3064581861	1.3387351929
C	2.5426087647	-1.2447234595	0.540322659
N	1.5018905967	-0.257107076	0.1740116286
H	-0.3463074685	-2.1988363918	1.7878290933
H	1.5911275527	-3.2155033696	0.7536191434
H	2.2549016323	-2.5747717722	2.2736090017
H	3.0069052621	-1.6541638173	-0.3595627166
H	3.3040266793	-0.7555977067	1.149759232
N	-0.7590142901	0.1876294008	0.707356795
H	-0.8697692782	0.7345687614	-0.1591005714
C	-1.9789302363	-0.0657514292	1.4898376865
C	-2.8483047453	-1.1667343283	0.9766063264
H	-1.6917430841	-0.2604146491	2.5279385079
H	-2.5534317826	0.8618205154	1.4783569686
O	-2.4392035796	-2.4520459452	1.2493264176
C	-3.3555786775	-3.2993364085	0.6871815802
C	-4.3365496213	-2.5858803007	0.0731668003

C -4.0064581505 -1.20110071 0.2595107243
H -4.5597071735 -0.3416283614 -0.0928903759
H -5.1930191485 -2.9911480658 -0.4471309022
H -3.1676489762 -4.3542086825 0.8192388332
C 1.8192656974 1.036149226 -0.4351497455
C 1.2671803547 1.5176105267 -1.5534870922
C 2.7715316943 1.8190891286 0.4074858672
C 0.2112305393 0.9367166612 -2.3017192786
O 3.2996656323 2.8797058129 -0.235575633
O 3.0546413777 1.5498083655 1.5676727979
C 4.1863345974 3.6982134015 0.5470950157
O 0.5980001053 0.3954546608 -3.4916603545
O -0.9963028947 1.0193528583 -1.9931814699
C -0.4594989449 -0.0805383484 -4.3383923392
H 4.5175963985 4.4933126189 -0.1216443744
H 3.6666882294 4.1252332687 1.4091511331
H 5.0454121546 3.119742885 0.8973074638
H 0.0337995397 -0.4961021018 -5.2186778089
H -1.0492112016 -0.8574614813 -3.843429074
H -1.1253192492 0.7339537711 -4.6382674268

TS2(1a_t1)
C 1.2824862565 0.3106807691 1.7458983302
N 1.6618262592 0.0134395102 3.0150022732
C 2.5128192962 1.0939302111 3.5451986461
C 2.1298065362 2.2712548867 2.6348062002
N 1.6240071408 1.5788251931 1.440930926
H 1.8816842505 -0.9414313864 3.2666052363
H 3.5704791928 0.8291205642 3.4405528564
H 2.2931116282 1.2833365342 4.5967475965
H 2.9813870536 2.9072489807 2.3882405575
H 1.3405505961 2.8871296232 3.0802426675
H 1.1632624745 2.0951328797 0.6918444282
N 0.6536750299 -0.5339180186 0.9253895514
C 0.3173192133 -1.9127203258 1.3607452906
C -0.9915873212 -2.3726692851 0.8218872546
H 1.0957999214 -2.6020596537 1.0284008758
H 0.2754774028 -1.9266388445 2.4528001912
O -2.119913673 -1.7917354664 1.3402346331
C -3.1900714753 -2.3774099223 0.7284669344
C -2.7729343216 -3.3202805028 -0.1600574685
C -1.3396970666 -3.3161430933 -0.1002136668
H -0.655444678 -3.9299488214 -0.6689345926
H -3.4029400253 -3.9408245279 -0.7817542695
H -4.1626257723 -2.0188758283 1.0314703394
C 0.3284179544 -0.0701714743 -0.439474124
C -0.3693383226 1.0269353622 -0.6184939401
C 1.0026127082 -0.9057556508 -1.4772979377
C -0.7024473915 2.2930577675 -1.0068812213
O 0.6192098471 -0.577609354 -2.7252645475

O 1.8132337486 -1.7903328038 -1.2405547306
C 1.2444094625 -1.3267833665 -3.7829205918
O -1.9990794468 2.4697146271 -1.4466193326
O 0.0946130197 3.2619195159 -0.8994049612
C -2.3976984902 3.8317743492 -1.6256716856
H -2.3355467675 4.3995282303 -0.6908830703
H -1.7901006488 4.33763486 -2.3822893386
H -3.4370826357 3.7941696905 -1.9592264235
H 0.8205409468 -0.9365614655 -4.7085327513
H 2.3280792215 -1.182820456 -3.775822164
H 1.0226538754 -2.3930263076 -3.6877689713

TS2(1a_t2)
C 0.5365006327 -0.6283455867 0.8246480122
N 0.6287957107 -1.6507327073 1.7098930756
C 1.9815388457 -2.2336676702 1.6430004881
C 2.7939766747 -1.1121217113 0.9697499574
N 1.7418485246 -0.2775785512 0.3491716611
H -0.1631953828 -2.2736934672 1.8187039577
H 1.9672360135 -3.1471669466 1.0396161264
H 2.3515947531 -2.4719374779 2.6413414728
H 3.4790557439 -1.487868896 0.2069622031
H 3.353195122 -0.51469981 1.6913697896
N -0.611216894 -0.0286100047 0.5061190073
H -0.5946030903 0.6455066702 -0.2571407973
C -1.9086061784 -0.3345133824 1.1274505645
C -2.637606114 -1.4825410659 0.5124979309
H -1.7520129181 -0.5081085534 2.1971159819
H -2.5227887932 0.5614197461 1.0271311227
O -2.1589668973 -2.7388300184 0.8006301173
C -2.9527592794 -3.6366999147 0.140205419
C -3.923831584 -2.9832527528 -0.5518303561
C -3.718068047 -1.5828270291 -0.3115297736
H -4.2982209255 -0.7576426187 -0.7005714853
H -4.6940307864 -3.4381573537 -1.1587382957
H -2.6984134118 -4.6779524825 0.2691552841
C 1.9464060938 0.8362521057 -0.5724198596
C 1.1698372776 0.985041343 -1.6223663338
C 2.9905042014 1.7930852026 -0.0921662612
C 0.2614818054 1.437717093 -2.5358949997
O 3.3280060743 2.692693953 -1.0356648557
O 3.4882435583 1.7833613224 1.0246374665
C 4.2979032101 3.6770013081 -0.6340825706
O 0.5792570421 1.1689488505 -3.8576340997
O -0.8186608549 1.9997218778 -2.2246792404
C -0.4615320923 1.4477293313 -4.7968665625
H 4.4467353538 4.3143043712 -1.5061044678
H 3.9272528174 4.2701948131 0.2060596202
H 5.2406207742 3.2017283019 -0.3501832727
H -0.0612929585 1.1678167878 -5.7740728461

H -1.3637316087 0.8609023249 -4.5919735892
H -0.7313124126 2.508497598 -4.8045645913

11a_t1

C 1.4600831831 0.2739955056 1.9413281309
N 1.9560086834 -0.2767413769 3.0795598672
C 2.6862180811 0.7565235159 3.8395887151
C 2.0999016702 2.0557267513 3.2587044794
N 1.6154698935 1.6079183358 1.9461111268
H 2.3330763719 -1.2161594468 3.0627306062
H 3.7612437805 0.6784395992 3.6458929234
H 2.5071071208 0.6524922911 4.9106882701
H 2.8467066316 2.8448461654 3.157940063
H 1.266005304 2.4287884433 3.8636725253
H 0.9340917413 2.1194120675 1.3680824031
N 0.8604932217 -0.4034367055 0.9569092613
C 0.5480674769 -1.8456566288 1.1401928717
C -0.8374182885 -2.1893299658 0.7151757038
H 1.2517059551 -2.4504372234 0.5672114976
H 0.6635044578 -2.0818149911 2.1999694504
O -1.8661281664 -1.6770445096 1.4625690414
C -3.0280055356 -2.1465014005 0.9215000208
C -2.7657293058 -2.9500918151 -0.1450581657
C -1.3378555496 -2.9765008871 -0.2808632885
H -0.7569155639 -3.5079692449 -1.0215227262
H -3.4938112632 -3.4588928956 -0.7611279588
H -3.9388349527 -1.8219698233 1.4024686359
C 0.4166897463 0.2767353796 -0.2684077765
C -0.1483783973 1.4895909488 -0.2155568324
C 0.8298935756 -0.4862831732 -1.4835040232
C -0.4839520677 2.2932248676 -1.3611038545
O 0.1523606649 -0.1054036139 -2.5838697525
O 1.7002401739 -1.3456934472 -1.5092779008
C 0.5615261105 -0.7270566382 -3.815011137
O -1.8168271811 2.2143047532 -1.6714977845
O 0.2641264494 3.0914261472 -1.9234417997
C -2.2588001908 3.0980206198 -2.7092535185
H -2.0784018228 4.1451843365 -2.4474603761
H -1.7584422611 2.8824090918 -3.6584240324
H -3.3315336937 2.9234719655 -2.812239271
H -0.0730136976 -0.2926232433 -4.5877342662
H 1.6125973281 -0.5155211478 -4.0282303999
H 0.4135153163 -1.8092176071 -3.770971729

11a_t2

C -0.1014425753 -0.8098368241 1.2870585361
N 0.171954135 -1.7027489362 2.2758444823
C 1.6077400092 -2.0270283505 2.239649117
C 2.2016449542 -0.8164745761 1.5014685569
N 1.0283897368 -0.259528194 0.7881304013

H -0.4877841154 -2.4594243106 2.4207490942
H 1.7729486278 -2.9602465491 1.6902249579
H 2.0121252376 -2.1309951766 3.2476875777
H 2.9842436379 -1.1008833228 0.7968680413
H 2.6037007649 -0.0724346195 2.1899470858
N -1.3078062649 -0.4839563477 0.8526149034
H -1.2349504183 0.1765205615 0.0124825637
C -2.5695479416 -0.9692135235 1.4219793567
C -2.9708265852 -2.3385174317 0.9800530007
H -2.5120564185 -0.9301439934 2.5159209662
H -3.3430281513 -0.2657403584 1.1089640921
O -2.3095396544 -3.3913771175 1.5690774946
C -2.8070905854 -4.5429229594 1.0210538528
C -3.7697933265 -4.2478424029 0.1075916502
C -3.8753391062 -2.8160353449 0.0797430722
H -4.5387596168 -2.2190344422 -0.5304803712
H -4.3389497318 -4.9585861982 -0.4750935632
H -2.3804044857 -5.4642608121 1.3881693043
C 1.0571735795 0.74841209 -0.2470832434
C -0.0547271613 1.0542820646 -0.9377489668
C 2.3597529689 1.4778120239 -0.3350401317
C -0.1524681121 2.1197287065 -1.9057705615
O 2.5965900628 1.9326226296 -1.5802608715
O 3.1150342439 1.6922858601 0.6013610706
C 3.7472210877 2.7862371308 -1.7246575461
O -0.1455952876 1.6461301394 -3.1908328202
O -0.3698048754 3.3039535197 -1.6583043294
C -0.3707986394 2.6283340663 -4.210223117
H 3.7757117021 3.0625095027 -2.7787021674
H 3.6473173582 3.6799137941 -1.1032253737
H 4.6611634793 2.2542365557 -1.4490835718
H -0.345952271 2.0858226681 -5.1572209056
H -1.3432339512 3.1149920775 -4.0882791534
H 0.4093106898 3.3957504011 -4.2051254841

TS3(1a_t1)
C 1.3909689297 0.2967786832 1.9362518792
N 1.8235219226 -0.2450805441 3.1193781513
C 2.3334042699 0.8621188432 3.9567363239
C 1.6715740217 2.0990293906 3.3096328679
N 1.3131229891 1.6149074086 1.9712786462
H 2.3845481506 -1.0877428085 3.0878937126
H 3.4258685427 0.9129082426 3.8942898108
H 2.0442353235 0.7245089079 5.0002041283
H 2.3474955288 2.9552884223 3.2561373522
H 0.768358376 2.4039039234 3.8509429424
H 0.708020638 1.9881577665 1.0440734166
N 1.0179806712 -0.4293293883 0.8530389454
C 0.8034216574 -1.8938007228 0.9916558351
C -0.5977830924 -2.3126421115 0.6969824064

H 1.4754417372 -2.4259074045 0.3195522759
H 1.0517502669 -2.1721042997 2.0157409197
O -1.5794861746 -1.854862958 1.5374799988
C -2.7589354238 -2.3932314071 1.1098686238
C -2.5529854293 -3.1878130379 0.0245383916
C -1.1451538781 -3.1331486777 -0.2464772353
H -0.6078691669 -3.6359954273 -1.0381342292
H -3.3058041654 -3.7404887054 -0.5198721456
H -3.6370955061 -2.1158568983 1.6739387953
C 0.5546262521 0.2496924734 -0.3448921076
C 0.2213358317 1.5520198796 -0.3216324467
C 0.7031436736 -0.5664911104 -1.5901781389
C -0.0946694943 2.3500219918 -1.4922166249
O -0.1998871514 -0.2222731069 -2.5255014256
O 1.5680119255 -1.4087541801 -1.7759889102
C -0.0305994972 -0.8384419404 -3.8165960288
O -1.42964707 2.6100184001 -1.5812468069
O 0.7220324672 2.8648655223 -2.2482544372
C -1.8170014397 3.5006756308 -2.6387734079
H -1.3381994078 4.4780187616 -2.5287906774
H -1.5593725341 3.0880968627 -3.6185535503
H -2.8998924465 3.6065190653 -2.5555035769
H -0.8291807905 -0.4353249724 -4.4391769881
H 0.9440744 -0.5854854103 -4.2413876692
H -0.1227909073 -1.9245930642 -3.7407020167

TS3(1a_t2)
C -0.1174906786 -0.7726897889 1.3814228674
N 0.1716685957 -1.664120139 2.3726283445
C 1.6129120237 -1.961440039 2.3383192116
C 2.1898649189 -0.7287737042 1.6230639259
N 1.01551784 -0.200341458 0.8945622647
H -0.4708584698 -2.4371951368 2.5033224035
H 1.8010748557 -2.8812268663 1.7732668373
H 2.0151097448 -2.0750235439 3.3463366108
H 2.994047958 -0.9880258313 0.9343479011
H 2.5602851261 0.0177221981 2.3279134477
N -1.3094374209 -0.4506695443 0.9249137813
H -1.1652923439 0.2940493251 0.0565881509
C -2.5634525109 -0.9934691347 1.4538863349
C -2.9357099266 -2.3364409807 0.9146269595
H -2.5175330643 -1.0284363984 2.5496976581
H -3.3537139855 -0.2892901766 1.1846711612
O -2.2119328103 -3.4092245676 1.379997332
C -2.6973239083 -4.5284476421 0.7591775992
C -3.712037127 -4.1942429947 -0.0816456429
C -3.8658441575 -2.7698108182 0.0176696864
H -4.5761867615 -2.1483972607 -0.5097849658
H -4.2836530502 -4.8742329866 -0.6977443877
H -2.2222754355 -5.4608144457 1.0252665615

C 1.0115628025 0.800017173 -0.1371980374
C -0.1422686314 1.1276039478 -0.7501642407
C 2.3269827352 1.4815795746 -0.3406453655
C -0.2837724389 2.1145007803 -1.8008989772
O 2.4659682955 1.9320283205 -1.6005037454
O 3.1701105216 1.6583356684 0.5248102651
C 3.6384903839 2.7297681223 -1.85482288
O -0.3264224924 3.3920329817 -1.3122490704
O -0.484513958 1.8689793502 -2.9851022197
C -0.6141510473 4.4126875204 -2.2801364487
H 3.5778627222 3.0097068994 -2.9064185806
H 3.6429402806 3.6231163797 -1.2252695303
H 4.5465002428 2.1508742662 -1.6694477601
H -0.6408886883 5.3511833263 -1.7237043697
H 0.1626240963 4.460013907 -3.0490215438
H -1.5799522362 4.239921717 -2.764009539

12a_t1
C 1.0069565394 0.1888632048 1.8353362512
N 1.8055696703 -0.5215503062 2.7239442252
C 2.7547359256 0.4659096878 3.2786861107
C 2.0794435123 1.8104322387 2.892872754
N 1.0582267804 1.4784062564 1.8841025389
H 2.2194283018 -1.3784656322 2.372444639
H 3.7402607298 0.3559276242 2.8112394002
H 2.8689211782 0.3400331879 4.3585724815
H 2.8003695519 2.5364779288 2.5013401009
H 1.5960452696 2.2750436227 3.7627544197
N 0.1454292935 -0.4838666035 0.9810597698
C -0.0639946682 -1.9450405076 1.0976987705
C 0.9079168669 -2.7569230284 0.3063891446
H -1.0883673456 -2.1574920946 0.7960409384
H 0.0201433549 -2.217823668 2.1505552958
O 0.7846772686 -2.7168904753 -1.0575715965
C 1.7555158759 -3.5257744088 -1.5754810975
C 2.48795126 -4.0879715528 -0.5761633368
C 1.9359090937 -3.5874821432 0.6506543743
H 2.255068833 -3.8202779824 1.6575585067
H 3.315699109 -4.7734684195 -0.692994707
H 1.7892920669 -3.5948122924 -2.6526867102
C -0.3851474181 0.2483308641 -0.0885854885
C -1.677640553 0.270878698 -0.4880936077
C 0.6438637616 0.9635999374 -0.9443577815
C -2.8380648881 -0.2791792107 0.2325578295
O 0.1417882383 2.0622054831 -1.5253346273
O 1.7696645155 0.5467616346 -1.1249712926
C 1.0275897583 2.7479959863 -2.4366610581
O -3.967254744 -0.029508423 -0.4661220386
O -2.8369679304 -0.8493759215 1.3130367685
C -5.1938017818 -0.4727303723 0.1461180191

H -5.1775353387 -1.5541533474 0.3033419372
H -5.351116521 0.0302285409 1.1036562166
H -5.9843768108 -0.2046384189 -0.5545371656
H 0.4610075127 3.6022379928 -2.8059709881
H 1.9251806131 3.0844417252 -1.9128303172
H 1.3099024809 2.092471926 -3.2638502338
H -1.9085183624 0.8001982691 -1.4036114451

E-**12a_t1**
C 1.012141576 0.4511045169 2.1184954934
N 1.8740966592 -0.152153206 3.0371563293
C 2.1966865142 0.8887461592 4.0325815413
C 1.0947062036 1.9426359593 3.7556499453
N 0.5164324657 1.5926476749 2.4464499976
H 2.6722160864 -0.6411480199 2.6446659309
H 3.2006191771 1.2912644292 3.8554723928
H 2.1581673206 0.4886317508 5.0489568583
H 1.4845335481 2.9655290283 3.7486545953
H 0.3029952809 1.9022337574 4.5146045962
N 0.7238785979 -0.249019308 0.9333275952
C 0.8715305642 -1.7328809879 0.9622593395
C -0.3713834085 -2.4777958108 0.6071655784
H 1.674763804 -2.0500048468 0.2949627911
H 1.1614539071 -1.98910712 1.980931568
O -1.5101455264 -2.1728658704 1.3039500226
C -2.4820421946 -3.0348438164 0.8817138096
C -1.9857037543 -3.8880899381 -0.0548938449
C -0.6095468471 -3.5244734874 -0.2354327367
H 0.1087639957 -3.9787045649 -0.9035273509
H -2.5293664716 -4.6734522507 -0.5611477988
H -3.4549101079 -2.9070928975 1.3325994905
C 0.3320018819 0.3907361024 -0.2397035684
C -0.0595045636 1.6878290382 -0.3485394138
C 0.453633715 -0.4541932337 -1.4996469203
C -0.2435431199 2.3554194092 -1.6400165168
O -0.7370828123 -0.6550765437 -2.0650156521
O 1.5090174523 -0.8969441475 -1.9061771687
C -0.7216378197 -1.3541611286 -3.3266612172
O -0.8566313998 3.551430736 -1.4616624224
O 0.1118144566 1.9575999631 -2.7415369097
C -1.0589285056 4.3324661488 -2.6522273379
H -0.1045171198 4.5652799556 -3.1322849929
H -1.6978287533 3.8015978907 -3.3628935357
H -1.5468795191 5.2502640933 -2.3230096235
H -1.7661987021 -1.443273118 -3.6232259191
H -0.1639757942 -0.7753690542 -4.0662929804
H -0.2726506866 -2.3433375888 -3.2149188551
H -0.1911470998 2.2813443262 0.5429378899

12a_t2

C 0.814686568 0.7614530555 0.5927385333
N 1.7086439656 1.7931250533 0.3887720687
C 0.9986203891 3.019045329 0.0041431992
C -0.3083732536 2.47916191 -0.5913801495
N -0.4173915718 1.1513046581 0.0427109029
H 1.5698027176 3.5985770585 -0.7246438392
H 0.8028418911 3.6504038962 0.8791889851
H -0.239861685 2.3522607672 -1.6777722793
H -1.1645073267 3.1051833978 -0.3478978957
N 0.9626945914 -0.3874576828 1.1468040883
C 2.2991801545 -0.7501086115 1.6301117325
C 2.6054163805 -0.2477605123 3.0077420187
H 3.1017357409 -0.3912517465 0.9701232104
H 2.3566617704 -1.8455023585 1.6316834151
O 1.8174304388 -0.7084347168 4.0308393465
C 2.2685240578 -0.1303698578 5.1842099884
C 3.3256287856 0.6849384209 4.9238232487
C 3.5448375445 0.6094968546 3.5061600132
H 4.3068132119 1.1189818701 2.9319931016
H 3.8815165838 1.2671821395 5.6457055138
H 1.738849234 -0.3984120486 6.0864144193
C -1.4164881073 0.2328316463 -0.2207663072
C -2.7525404767 0.4714419426 -0.2457592645
C -0.959706707 -1.1317926475 -0.709674562
C -3.4498691851 1.6811938056 0.2055097364
O -0.1561501873 -1.2727437446 -1.6088589027
O -1.6346829242 -2.1287374579 -0.1249260974
O -4.7737481493 1.5727598261 -0.0569731484
O -2.9675155669 2.6490958868 0.7770426203
C -5.5917772429 2.6705286656 0.3874488771
H -6.6084372457 2.4207056406 0.0835661952
H -5.5390903205 2.7784638348 1.4740246675
H -5.2759571793 3.6050409309 -0.0834127745
H -3.394210922 -0.3324546033 -0.5851039906
C -1.3356144809 -3.4547053214 -0.6139163325
H -1.5709450246 -3.5342030629 -1.677809439
H -0.2808287315 -3.6893474633 -0.4529505815
H -1.9661357191 -4.1282666258 -0.0344055676
H 2.4521129815 1.8877308718 1.0695192493

E-**12a_t2**
C -0.4117845793 -0.7447301206 1.3444665296
N 0.1620426338 -1.6596976056 2.2118674537
C 1.6095821754 -1.7320258277 2.007425966
C 1.9161186527 -0.3413419895 1.4538460143
N 0.6380618185 0.0363731682 0.8087306163
H -0.3297692281 -2.5393826416 2.3120909287
H 1.8731350315 -2.5145910519 1.285098109
H 2.1373893342 -1.9229634337 2.9440407538
H 2.7306753787 -0.3588707125 0.7290389345

H 2.1563966071 0.367082496 2.2523455471
N -1.6501259531 -0.5562980853 1.0635770259
H -1.5233851366 1.007170861 -0.4662011236
C -2.6462737952 -1.3972391729 1.7423865518
C -2.8933298395 -2.7159003092 1.0797796882
H -2.3853643596 -1.5672567231 2.7983044676
H -3.5950131098 -0.8529841731 1.7336066775
O -1.946232833 -3.6959996482 1.2716140304
C -2.3482023429 -4.7987902624 0.5669064154
C -3.5257129237 -4.5465126023 -0.0638478337
C -3.8786822199 -3.1941564601 0.2675335492
H -4.7535169544 -2.6473234818 -0.0561493963
H -4.0788179414 -5.2354643923 -0.6870696465
H -1.6997783885 -5.6601728485 0.6247976717
C 0.5320754508 1.1217089832 -0.0387143488
C -0.6072494119 1.54841824 -0.6481737903
C 1.8589735164 1.8410864938 -0.2282096872
C -0.7414879633 2.6920034605 -1.5478197644
O 2.4583004199 1.4597352631 -1.3588601853
O 2.3233459773 2.6053938566 0.5905464112
C 3.7280339159 2.0931495798 -1.6422708752
O 0.3635123974 3.4729652725 -1.643242485
O -1.7733359095 2.9436694334 -2.1559752882
C 0.2628159902 4.5992238955 -2.5323151886
H 4.0680548498 1.6546054881 -2.5795967592
H 3.5944927189 3.1714291197 -1.7521184899
H 4.4440484732 1.8889134036 -0.8433214373
H 1.2228716524 5.1117794284 -2.4689257677
H 0.0790683122 4.2699189572 -3.5583808047
H -0.542117417 5.2688861417 -2.2190894697

$TS4_{5C}$(1a_t1)
C 1.4052179191 -0.1701762376 1.4064779512
N 2.1385876886 -0.8322449764 2.3069922595
C 3.3117992145 0.0049806146 2.6336589859
C 3.3531606391 0.957202725 1.4106783819
N 1.954704564 0.9411449261 0.9549018379
H 4.20855133 -0.6085560187 2.7313601741
H 3.1423646162 0.5504558492 3.5683681387
H 3.9992667908 0.5657067081 0.6162439655
H 3.6790671328 1.9631140901 1.6788166302
N 0.2351613155 -0.5295379556 0.8000873318
C -0.4952035172 -1.7696840397 1.1509978559
C -0.1837750253 -2.9159243562 0.2521841444
H -1.5592252751 -1.5505287416 1.0838953561
H -0.2562867631 -2.0061985668 2.1909598596
O 1.0422308889 -3.5166200442 0.3874873457
C 1.0982614416 -4.5252807298 -0.5309150273
C -0.0601480146 -4.5849447723 -1.2429992006
C -0.8945092427 -3.5348704828 -0.7343466767

H -1.8923090192 -3.2707730808 -1.0548192964
H -0.2939093344 -5.2862849499 -2.0317320613
H 2.0130955626 -5.0989966538 -0.5480738994
C 0.0123928775 0.3251912498 -0.2826488694
C -1.0698015416 0.5342782223 -1.0629082581
C 1.3144214724 1.1702843745 -0.5809924238
C -2.4262373905 -0.0093656117 -1.0108645372
O 1.1992217391 2.3448623673 -0.9775139349
O 2.187936673 0.2650305698 -1.3266971242
O -3.1070935347 0.3600392545 -2.1230197461
O -2.9534353249 -0.6707828087 -0.1221582301
C -4.4798641418 -0.064086783 -2.1898574634
H -4.5511945325 -1.1550390875 -2.1876845402
H -5.0527048457 0.3352542572 -1.3488203353
H -4.8644090539 0.3344506467 -3.1290124574
H -0.902437594 1.2716133153 -1.8412446273
C 2.6547119624 0.7941777178 -2.5599192523
H 3.3487565386 0.0510606306 -2.9641861329
H 1.8430440145 0.9470108226 -3.2841543393
H 3.1804809754 1.7461798217 -2.4326146312
H 1.7819957947 -1.5523682657 2.9207598464

TS4$_{5C}$(1a_t2)
C 0.8725676343 1.2374215116 0.4994801252
N 1.8009972519 2.1645175265 0.7705741927
C 1.3019900815 3.5046543922 0.412994823
C -0.2262771344 3.2939576604 0.3565540948
N -0.3267040311 1.8369428756 0.13864852
H 1.7038667574 3.8156048905 -0.556800926
H 1.5853834478 4.2373330769 1.1698705076
H -0.7079046375 3.8429006303 -0.4469380741
H -0.7065290984 3.5544106562 1.3051872206
N 0.8222524315 -0.0530526567 0.5595729109
C 1.8161839902 -0.9701983687 1.091054025
C 2.9165737404 -1.2991877649 0.1383265082
H 2.2360358276 -0.5670610326 2.0215793913
H 1.2689501663 -1.8831072661 1.3450361702
O 3.8266090231 -0.2988597531 -0.1054918104
C 4.7402561192 -0.7906846615 -0.9972031077
C 4.4374535989 -2.0767364785 -1.3191266228
C 3.2498529035 -2.4068898866 -0.5819575075
H 2.7139466102 -3.345847063 -0.5867620363
H 4.9879521431 -2.7148625965 -1.9962748456
H 5.5275262863 -0.1126123886 -1.2908931077
C -1.3612841084 0.9348958665 0.121139002
C -2.6995840401 1.1132106809 0.0025055117
C -0.7962534843 -0.5270667196 0.284818589
C -3.4255577616 2.3405276609 -0.2991612709
O -0.6615778554 -1.0638727412 -1.0477517138
O -1.251303738 -1.2575845424 1.1879957075

O -4.7617775057 2.1457404519 -0.1386493635
O -2.9685356587 3.411921579 -0.680628472
C -5.5979292067 3.2711308405 -0.4524252248
H -6.6213559232 2.9349074847 -0.2825751464
H -5.3708156546 4.1209223099 0.1972268134
H -5.471360424 3.5748813274 -1.4952168344
H -3.3018969683 0.2205111054 0.1274868983
H 2.787286015 1.9382947941 0.7739378121
C -1.402121852 -2.2558146515 -1.2830195826
H -2.4860277485 -2.0752305628 -1.3100499839
H -1.0867534963 -2.6201493739 -2.2650274626
H -1.1997197013 -3.0231828132 -0.5300727305

TS4$_{6C}$(1a_t1)
C 0.8874830869 0.2961590419 2.1322438096
N 1.3663662653 -0.1480673424 3.335002299
C 1.4010643256 1.0121109574 4.2493632732
C 0.4252435525 1.9892106081 3.5641211646
N 0.3944829813 1.5172993911 2.1722046556
H 2.4138250908 1.4266688781 4.297128761
H 1.0868759809 0.7253038546 5.254643832
H 0.7602937894 3.0264216185 3.6233584044
H -0.5856468609 1.9190602947 3.9813250026
N 0.8656212502 -0.4568917458 1.001136512
C 1.120884049 -1.9222044448 1.0578315448
C -0.0500844348 -2.7359970072 0.6227694435
H 1.9736565123 -2.1648600019 0.4260226511
H 1.3730034865 -2.1700069853 2.0888011829
O -1.1648022054 -2.706450895 1.4204343507
C -2.0870868938 -3.5372554277 0.8532083563
C -1.5868857725 -4.100264178 -0.2803282724
C -0.2605333423 -3.5757121988 -0.4326422748
H 0.4454421136 -3.7904783996 -1.2223070102
H -2.0964448073 -4.7974165403 -0.9305943984
H -3.0329302401 -3.6158502215 1.3685575688
C 0.3116329457 0.1303465592 -0.1713258607
C -0.4349331614 1.2490007419 -0.1345021003
C 0.7642121001 -0.4452802308 -1.4717334164
C -0.9011965387 1.9389915014 1.1325369294
O -0.1012671728 -0.1844595783 -2.4647383984
O 1.8125683146 -1.0483088392 -1.6308527173
C 0.3126517894 -0.6007816192 -3.7829545806
O -0.6556094856 3.3382088264 0.9325866276
O -2.0052428829 1.5787439727 1.6149136951
C -1.5318382437 4.2091794041 1.6373420738
H -1.2975842889 4.2674959767 2.7092822255
H -1.3928017705 5.2022868277 1.1997356034
H -2.5772451007 3.9037009795 1.5310327704
H -0.490504649 -0.2898930574 -4.4505266862
H 1.2498314637 -0.1148002841 -4.0645380674

H 0.4374975772 -1.6854340814 -3.8197862043
H -0.7865737614 1.6708533771 -1.0684919184
H 2.1698419374 -0.7658817325 3.3427491685

TS4$_{6C}$(1a_t2)
C 0.2368019703 -0.5665769531 1.1471069737
N 0.2084885607 -1.4469113083 2.1888637851
C 1.5748339741 -1.8994824234 2.4841365077
C 2.4247357534 -0.7585201332 1.9116864456
N 1.5275055931 -0.2020664811 0.8706320604
H 1.7876121004 -2.8483175414 1.9786187696
H 1.7242735774 -2.0256604806 3.5576750411
H 3.3577771247 -1.1037257951 1.4768425391
H 2.6387584903 0.0070555484 2.6657570028
N -0.7906121157 -0.0643218639 0.5156213631
C -2.1654339993 -0.3588431565 0.9252154731
C -2.7228745506 -1.6359940478 0.391867272
H -2.2369692104 -0.3473485466 2.0200029656
H -2.7527144167 0.4815775953 0.5443476874
O -2.2869734241 -2.7963648328 0.9902937978
C -2.9132366018 -3.8402216117 0.3641470278
C -3.7391554182 -3.3730140835 -0.6093959021
C -3.6148430144 -1.9424071964 -0.5921051299
H -4.1235138262 -1.2327091556 -1.2294738689
H -4.3626511185 -3.9695128793 -1.2607570148
H -2.6680909529 -4.8268018402 0.7277355857
C 1.7785214811 0.9278872981 0.0655534065
C 0.7936607306 1.5398478518 -0.6200366511
C 3.2033376657 1.3401504681 -0.0962114414
C -0.6699203242 1.1632989805 -0.6604089492
O 3.3117552707 2.6019416439 -0.5438201045
O 4.1637610748 0.6212972691 0.1216270008
C 4.653252153 3.0523342997 -0.8276952994
O -0.8474973383 0.343058156 -1.8536066356
O -1.5123036775 2.0689590082 -0.4396966465
C -1.7467909926 0.880757839 -2.8136738562
H 4.5450141718 4.075526313 -1.186233679
H 5.2642095861 3.0302404496 0.0776062978
H 5.1149061677 2.427434506 -1.5958859107
H -1.923583637 0.0889398014 -3.5482179374
H -2.6997413622 1.1830143467 -2.3700382209
H -1.3251013656 1.7516241633 -3.3378029709
H 1.0500454062 2.3908413104 -1.2391255709
H -0.5468405062 -2.1218475179 2.2220587869

13a_t1
C 1.4304025422 -0.2002710165 1.4805007565
N 2.1467790369 -0.8513704454 2.3956214832
C 3.3230138333 -0.019134995 2.7321420098
C 3.3879275439 0.9365656716 1.5123370692

N 1.9967220547 0.9118869802 1.037103099
H 4.2147265238 -0.6382872791 2.8373603935
H 3.1460955416 0.5242419846 3.6660691835
H 4.0492990722 0.5534078378 0.7282478833
H 3.6998075 1.9437582041 1.7913994918
N 0.2667759251 -0.5333722276 0.8564898305
C -0.4748863316 -1.7788080808 1.1633017027
C -0.1329483953 -2.9088731946 0.255177192
H -1.5354671692 -1.5566335367 1.0642112642
H -0.2671493101 -2.0312615914 2.2061775139
O 1.0848478512 -3.5167042921 0.4283259117
C 1.1755849877 -4.5071101273 -0.5069415178
C 0.0474384717 -4.5475502058 -1.267352594
C -0.8040529415 -3.5046444427 -0.7724952465
H -1.7864922175 -3.2295054846 -1.1288866663
H -0.1558822585 -5.2321182901 -2.0789170085
H 2.088504497 -5.0840629854 -0.4974984088
C 0.0709876788 0.3659555818 -0.1993705741
C -0.9848327659 0.6063363772 -1.0056492587
C 1.3940335388 1.2166877472 -0.4000420197
C -2.3322598752 0.0392409121 -1.0426106757
O 1.2975056064 2.4098809839 -0.7655812518
O 2.2857020085 0.3238403631 -1.1963809234
O -2.9781210111 0.4717534369 -2.1528279202
O -2.8795637629 -0.6908565929 -0.2223559719
C -4.3382223676 0.0282108283 -2.3018144325
H -4.3865771218 -1.0614009879 -2.3767730042
H -4.950494895 0.3580917204 -1.4584277237
H -4.695477822 0.4833797336 -3.2258971143
H -0.7972796947 1.3951407691 -1.7271361116
C 2.2501262306 0.5902448804 -2.5868677964
H 3.097576767 0.0626312614 -3.0371197815
H 1.3262925301 0.2221365248 -3.0606536003
H 2.3411307758 1.6624512384 -2.7916737744
H 1.7856914225 -1.5787662607 2.9990805916

13a_t2

C 0.9503841481 0.6457617411 0.5505232512
N 1.8832248643 1.5761735842 0.769505064
C 1.3799830574 2.9028069487 0.3655118597
C -0.149553506 2.6886597028 0.3224328802
N -0.2481273186 1.2225624789 0.169751485
H 1.7792921218 3.1774401526 -0.615949582
H 1.6660067944 3.6617312329 1.0948344211
H -0.6322233331 3.2007132962 -0.5045531251
H -0.627718609 2.991460575 1.2590870606
N 0.8940144656 -0.6449791537 0.6783167366
C 1.8953922284 -1.5501032841 1.221486438
C 2.9989528106 -1.8847125895 0.275321148
H 2.3090811949 -1.1289376045 2.1460374605

H 1.3463157303 -2.4583681065 1.4857996996
O 3.9190591779 -0.8908483487 0.0407563178
C 4.8334892566 -1.3869452903 -0.8477476691
C 4.5214020838 -2.6690411667 -1.1766069855
C 3.32686268 -2.991962176 -0.4478989573
H 2.7823954141 -3.9258898988 -0.4600630014
H 5.0704808522 -3.3093398734 -1.8528301897
H 5.6284842867 -0.7146744257 -1.1338360362
C -1.2706401189 0.3017663745 0.21259732
C -2.6138038139 0.4540016447 0.1289333913
C -0.6429691745 -1.1319021562 0.4024174493
C -3.3629312466 1.6622659963 -0.1953701504
O -0.4513100138 -1.6726338814 -0.947045226
O -1.1131912104 -1.8933808088 1.2789257262
O -4.6912702827 1.4611401749 0.0110601527
O -2.9263854141 2.7231161359 -0.6264090677
C -5.5475551486 2.5682546435 -0.3140175698
H -6.5616133994 2.2305047466 -0.0974879317
H -5.305882393 3.4424777416 0.296715566
H -5.4588673919 2.8349977989 -1.3707420613
H -3.1962051596 -0.4409200252 0.3150926964
H 2.8691905206 1.3497662661 0.794538168
C -1.493525038 -2.5384025599 -1.3699627936
H -2.4008984495 -1.9874472384 -1.6614095474
H -1.1236081469 -3.0769142722 -2.2482907224
H -1.7567955195 -3.2593393752 -0.5891496755

14a_t1
C 0.9423320182 0.2332475662 2.21397464
N 1.468592902 -0.2330782162 3.3803715594
C 1.4973838189 0.8847252004 4.3465901355
C 0.4746958898 1.8627708058 3.7423811308
N 0.4247353458 1.4493486025 2.3312340278
H 2.4996313716 1.3243093247 4.3834155717
H 1.2220938868 0.5408326228 5.3450060258
H 0.7791311353 2.9056870924 3.8316238035
H -0.5241007287 1.7416827039 4.1739050636
N 0.897324703 -0.4648085129 1.0563651678
C 1.1736631362 -1.9284344118 1.0408952682
C -0.0083615299 -2.741305977 0.6353649058
H 1.9939888774 -2.1290627039 0.3544073931
H 1.4841112034 -2.2143729035 2.0457783994
O -1.0696293808 -2.7798154691 1.5028047919
C -2.0109728149 -3.5985645144 0.9500399943
C -1.5747046387 -4.0880562291 -0.242602824
C -0.27111426 -3.5269281121 -0.449580196
H 0.3858832341 -3.6818413137 -1.2935252137
H -2.11221478 -4.7600275978 -0.8968676855
H -2.9194885056 -3.7265387638 1.5197643905
C 0.2858714531 0.1695131703 -0.0689688806

C -0.4495758872 1.2864647948 0.0780666168
C 0.6669693486 -0.3478812528 -1.4113338036
C -0.780776422 1.9063952633 1.42582233
O -0.2344368236 -0.0124653999 -2.3499311878
O 1.6908449145 -0.9674670086 -1.6511094938
C 0.1084437443 -0.3707101067 -3.7046492294
O -0.4443041941 3.3405870756 1.337814214
O -1.9238431672 1.6094306022 1.8937538897
C -1.5534528687 4.1496045698 0.9904505533
H -2.4191590271 3.9491731066 1.63065211
H -1.2407542662 5.1912426955 1.1179133713
H -1.8643204253 4.0134133396 -0.0584959721
H -0.7154632604 -0.0071261614 -4.3182264434
H 1.0443949586 0.106238106 -4.0053320318
H 0.2029480387 -1.4547639575 -3.8016902726
H -0.8667663041 1.760946535 -0.8017792507
H 2.273670304 -0.847619565 3.3487061309

14a_t2
C 0.1156040954 -0.4090983661 1.0278422923
N 0.0650414549 -1.2728269992 2.0790157513
C 1.4275264575 -1.7003016392 2.4269654708
C 2.277775274 -0.5556993752 1.8625045213
N 1.4054078525 -0.0348685555 0.7828041244
H 1.6695846105 -2.6542873391 1.9450805544
H 1.5415810169 -1.8056685414 3.5069244282
H 3.2298822344 -0.8911281743 1.4628818455
H 2.4525892326 0.2281223419 2.6079776811
N -0.9087402359 0.0654036088 0.3592883972
C -2.2878309058 -0.2692720908 0.7416406242
C -2.7790950514 -1.5848328744 0.2399968767
H -2.3883430566 -0.2268025106 1.8327717031
H -2.8925237448 0.5393396178 0.3256403584
O -2.3947975458 -2.6980718426 0.9543090966
C -2.9446407683 -3.7896259802 0.3383835027
C -3.672271342 -3.3988780896 -0.7416334323
C -3.5635228167 -1.9687819427 -0.8061420579
H -4.0110529356 -1.3098531049 -1.5367780227
H -4.2224339129 -4.0455605956 -1.4108777386
H -2.7295143191 -4.7466786461 0.7894099567
C 1.6678003671 1.0712421589 -0.0538158865
C 0.6729295372 1.6761769953 -0.7295398121
C 3.0959723103 1.4411119217 -0.2582318644
C -0.8000821271 1.3367545242 -0.665929285
O 3.2235926454 2.6731719627 -0.7788813316
O 4.0449665726 0.7162198238 -0.0100545045
C 4.5702251734 3.0806395248 -1.0992953608
O -1.1249672115 0.6195059233 -1.9124561421
O -1.5757791387 2.275966284 -0.3292458177
C -1.6920424358 1.4508607339 -2.9132610396

H 4.4779705776 4.0833228741 -1.5156626281
H 5.1891874819 3.0991216079 -0.1993175796
H 5.0132289626 2.4039278062 -1.8338708623
H -2.0706284432 0.7886694501 -3.6986903924
H -2.5157899871 2.055217088 -2.5201491086
H -0.9517459879 2.1293369434 -3.3665529463
H 0.9191368451 2.50009937 -1.3874150811
H -0.6882387357 -1.9500108933 2.1157537095

15a_t1
C 1.6296645001 -0.6259157167 1.3098357307
N 2.3607694057 -1.4205270208 1.9957364746
C 3.5977091396 -0.6319861815 2.2463943792
C 3.5570934328 0.5725324606 1.2559934012
N 2.106925859 0.6376615123 1.0126561535
H 4.4847276705 -1.2536496419 2.0978483591
H 3.6059657257 -0.2827352671 3.2868086318
H 4.098673293 0.3584642839 0.328222103
H 3.9359352895 1.4988216602 1.692012963
N 0.3851381822 -0.8062732413 0.7183132558
C -0.4811012254 -1.9286088407 1.1257273991
C -1.4318149395 -1.5902780524 2.2264658622
H -1.0506287063 -2.2502951568 0.257448405
H 0.1967301995 -2.7270278756 1.4377558392
O -0.9096328626 -1.4498215721 3.4878282982
C -1.9479872048 -1.1594870934 4.3244418307
C -3.1185500601 -1.1173058876 3.6314838929
C -2.7822851413 -1.3958059775 2.2650197486
H -3.4492089124 -1.4456806314 1.4169580609
H -4.0991318424 -0.9132731741 4.0385625892
H -1.6924411693 -1.0167047746 5.3640612221
C 0.1019612597 0.274357856 -0.0954826589
C -0.985206551 0.6449849781 -0.8186133097
C 1.3899137636 1.1484055743 -0.1309616062
C -2.2238302917 -0.0699984484 -1.1186893696
O 1.040381574 2.4919914314 -0.0446043294
O 2.1929112463 0.8840705633 -1.2641861529
H 1.8598733461 3.0120242778 -0.1110723531
O -3.1333797201 0.7941965257 -1.6326566426
O -2.4623728829 -1.2671533333 -1.0006028365
C -4.3851003036 0.2184868466 -2.0458556735
H -4.2276196039 -0.555222417 -2.8017664551
H -4.915983794 -0.2139844489 -1.1936683476
H -4.9621054627 1.0427666933 -2.4658781331
H -0.9191179792 1.6312208419 -1.2610671415
C 1.7212419584 1.3238553926 -2.5434617077
H 2.5597546171 1.1854860348 -3.2291651517
H 0.8728694639 0.723798386 -2.8888742344
H 1.4379527267 2.3804094343 -2.5295504965

E-**15a_t1**
C 1.7840984566 -0.6526709031 1.4886822652
N 2.4792422061 -1.5012848754 2.1477134498
C 3.7906849911 -0.8172917134 2.3161551047
C 3.7963105293 0.3634417309 1.2983545497
N 2.3470623939 0.5597766111 1.1404730165
H 4.6127971662 -1.5155070549 2.1382304509
H 3.8831810311 -0.4474236103 3.3454591551
H 4.2554913895 0.0704711402 0.3461866873
H 4.2904703405 1.2589188483 1.6794488062
N 0.5025425023 -0.7303719345 0.963246085
C -0.4116809613 -1.8422949996 1.248185824
C -1.5438445822 -1.4904436115 2.1556528817
H -0.8194520778 -2.2146781642 0.3048722626
H 0.2068795452 -2.6306354104 1.6857512113
O -1.2269384424 -1.1243649441 3.4370766521
C -2.4026254689 -0.8852330306 4.0896411217
C -3.4584189895 -1.0975531351 3.2580344603
C -2.898710023 -1.489992876 1.9961881901
H -3.4328126221 -1.7413873381 1.0903726638
H -4.5047498952 -0.9869365611 3.506105453
H -2.3161018217 -0.5765093212 5.1209499015
C 0.2817554959 0.3239826374 0.1069377802
C -0.8728857781 0.5452090091 -0.574389141
C 1.6113100176 1.1384095649 0.0275542267
C -1.1043770883 1.6627387113 -1.4738194746
O 1.466097251 2.4943592745 0.2218148469
O 2.1756849135 0.811092696 -1.231322668
H 0.8206953809 2.7811288643 -0.4729956248
O -2.2649727232 1.5121532603 -2.1387884058
O -0.3920617687 2.6598197106 -1.6456749218
C -2.6298694282 2.5777633398 -3.0378657741
H -2.7320428438 3.5219425459 -2.4973350679
H -1.8835257617 2.6900833536 -3.82794083
H -3.5881945744 2.2819274803 -3.4645978539
H -1.6994749425 -0.1444226532 -0.4578003027
C 3.0995637975 1.7569249452 -1.7866995959
H 3.5586441248 1.2478195134 -2.6372861355
H 2.5892265636 2.6574064629 -2.142077161
H 3.8836516962 2.0471984367 -1.0805020893

15a_t2
C 0.498140491 -1.6749327962 0.5108876721
N 0.5037083634 -2.9282378323 0.7680254137
C 1.2619488315 -3.5539755554 -0.3405416717
C 1.5489713645 -2.4450051805 -1.4030013723
N 1.1446139952 -1.2460984245 -0.6559602973
H 0.6852587519 -4.3758766902 -0.7759228854
H 2.1959858273 -3.9794089246 0.0452441143
H 0.9366402175 -2.5571617118 -2.3016574686

H 2.5964314909 -2.3909842688 -1.6941606029
N -0.0609758407 -0.5962364939 1.1313758928
C -0.5026773242 -0.6218773686 2.5279931907
C -1.8034385065 0.0770512943 2.7473598604
H -0.5863649799 -1.6775735678 2.80101046
H 0.2510521701 -0.163255019 3.1748946572
O -2.9233250648 -0.4856295837 2.1949573234
C -3.9835760731 0.3020258471 2.54164621
C -3.5689402343 1.3496210315 3.3041658421
C -2.1468196391 1.2042336364 3.4386277743
H -1.4679885994 1.846964288 3.9821235371
H -4.1916952037 2.1290313393 3.7202190681
H -4.9476926517 -0.0138105074 2.1714808984
C 0.9909455085 0.0922199571 -0.8607593149
C 1.258959927 0.9359473015 -1.8928756608
C 0.3725333029 0.6274025364 0.4607421204
C 1.8974415887 0.7072908005 -3.1838299096
O -0.6636564042 1.5028594456 0.1554980919
O 1.3227257734 1.2129304348 1.3255088048
H -1.019005222 1.8446002499 0.9953394222
O 2.4903878917 -0.5046243415 -3.3137321005
O 1.9240910305 1.5469905466 -4.0746428341
C 3.1144805749 -0.7639476594 -4.5834138639
H 3.5237357302 -1.7719298168 -4.5101173349
H 2.3838871383 -0.7133045716 -5.3949661983
H 3.916241807 -0.0463765187 -4.7757820166
H 0.9220165885 1.9565757226 -1.764210731
C 1.8781766613 2.4813036549 0.9551410092
H 2.4367375408 2.8231773177 1.8288215244
H 2.5625191545 2.3872593284 0.1056729501
H 1.099186022 3.2113810999 0.7170764253

E-**15a_t2**
C 0.3295406567 -1.6072453654 0.5056546516
N 0.3044514148 -2.8731981541 0.6979528108
C 1.0083912659 -3.4695047502 -0.464404512
C 1.358481563 -2.3096191634 -1.4593259913
N 0.9496479927 -1.1575243598 -0.6622039913
H 0.3710047813 -4.2188132878 -0.9437045874
H 1.9161553288 -3.9803999671 -0.1249386446
H 0.7829510708 -2.3578197134 -2.3891763655
H 2.422745043 -2.2569281599 -1.7024707298
N -0.1468785153 -0.5282668005 1.1890342425
C -0.5142797031 -0.6049019712 2.6026357204
C -1.8867251198 -0.105760042 2.913053298
H -0.4248464558 -1.6599325743 2.8811445364
H 0.205804988 -0.0450969545 3.2076067169
O -2.9352635411 -0.7085254608 2.2723363932
C -4.0815055865 -0.1353770035 2.7469170109
C -3.7875909225 0.8089930908 3.6809078218

C -2.3558343764 0.8286699203 3.789359694
H -1.7546323601 1.453428226 4.4353158228
H -4.4962788625 1.4197155982 4.2228049399
H -5.0043770126 -0.5022764513 2.3226706845
C 0.9264740476 0.1841760972 -0.7901525438
C 1.3864979468 0.8982204977 -1.8586050638
C 0.3035904728 0.7166568408 0.5387908368
C 1.2677547119 2.3317525642 -1.9926577193
O -0.7708305611 1.5539752132 0.3468262449
O 1.3576378581 1.3059087811 1.2774847778
H -0.4219606088 2.2800147018 -0.2348547186
O 1.9521827164 2.7880430984 -3.0603446746
O 0.6165528016 3.1064021193 -1.2735815923
C 1.8613395259 4.2014885036 -3.3212385959
H 2.4530809316 4.3693993171 -4.221225475
H 0.8236417063 4.4993452101 -3.4909025969
H 2.2720402176 4.777252711 -2.4881662681
H 1.8585361846 0.3656735476 -2.6765347364
C 1.0685514405 2.4910817932 2.0334411618
H 1.9553000467 2.6586232706 2.649015276
H 0.9183879754 3.3567491265 1.3813962365
H 0.1968819367 2.3738139505 2.6828959292

16a_t1
C 0.8738242985 0.2076299076 2.2763306237
N 1.3614127605 -0.3188179121 3.3494773716
C 1.2766602423 0.7218983867 4.3882254587
C 0.3722475779 1.8374848357 3.8105850094
N 0.4039836877 1.5145179124 2.3718778575
H 2.2817789887 1.1097885164 4.6028403443
H 0.8807460385 0.3138021316 5.3235809549
H 0.7487311593 2.8441239032 3.9980430199
H -0.6530784421 1.7652329288 4.1961781252
N 0.8426570288 -0.4178146675 1.0416818097
C 1.249933946 -1.8403328878 0.9655930348
C 0.1322294456 -2.7668120248 0.6249200209
H 2.036821793 -1.95177155 0.2210957211
H 1.6605354569 -2.0734718259 1.9487126315
O -0.8614903485 -2.9257151773 1.5569869034
C -1.7537028199 -3.8243505128 1.0471146329
C -1.3525612294 -4.2470557332 -0.182502843
C -0.1258909726 -3.5558778123 -0.45917847
H 0.4864007588 -3.6328564421 -1.3465270512
H -1.8641566887 -4.9577893161 -0.816419071
H -2.6050039295 -4.0515895387 1.6715766678
C 0.2594303153 0.2592330631 -0.033560866
C -0.4083082818 1.4168377061 0.119472123
C 0.5392883023 -0.2521102468 -1.4176611494
C -0.5946795336 2.0485801319 1.4705616844
O -0.4699417196 0.0186247293 -2.2551230687

O	1.5737836903	-0.8008581556	-1.7500584782
C	-0.2463044958	-0.3265206452	-3.640755885
O	-0.3651757641	3.4446392924	1.4888567732
O	-1.9269790731	1.7686944568	1.8721356587
C	-1.2286047591	4.2477800807	0.6784978502
H	-2.2827060139	3.9971764829	0.8318099519
H	-1.0563818461	5.2805857217	0.9889090851
H	-0.9865923952	4.157027734	-0.3872197678
H	-1.1527579631	-0.0237675672	-4.1635695347
H	0.6192118281	0.2109851355	-4.0346575048
H	-0.0891459512	-1.4024322762	-3.7433286487
H	-0.8218344624	1.9208753338	-0.7418413214
H	-2.1213116288	2.323523901	2.6472253464

16a_t2

C	0.2590947781	-0.5599916299	1.4615403657
N	0.2193481861	-1.2098840518	2.5769066791
C	1.5996025428	-1.2498339635	3.0887115463
C	2.4142535828	-0.2413611411	2.2435287887
N	1.5090018206	-0.0603852868	1.0916204924
H	2.0069185035	-2.2650317652	2.9873100979
H	1.62453871	-0.995813232	4.1529503659
H	3.381587865	-0.6223407496	1.923889945
H	2.5608877749	0.7145882406	2.7607265302
N	-0.8038604117	-0.372641516	0.6043878077
C	-2.1110091902	-0.9195570552	1.0188678621
C	-2.6926596676	-1.9090913832	0.0624054462
H	-1.9523673774	-1.3821750553	1.9952944431
H	-2.8264936114	-0.1039533924	1.1503937061
O	-1.955341191	-3.0261711238	-0.2212508166
C	-2.7022326325	-3.8012967686	-1.0640217808
C	-3.8997885498	-3.2083403384	-1.3168240826
C	-3.8947537783	-1.9739456298	-0.5827695204
H	-4.6824637839	-1.2343802031	-0.5352935308
H	-4.6884909243	-3.5947545852	-1.9471588708
H	-2.2437732792	-4.7238061863	-1.3880184853
C	1.6522164512	0.7763152976	-0.0008131639
C	0.5814373748	1.1388915875	-0.7330129331
C	3.0437139553	1.145052636	-0.4167788449
C	-0.8140499217	0.7398263383	-0.3487272482
O	3.0510514658	2.2036233268	-1.2389185596
O	4.0460005807	0.5516454275	-0.064072477
C	4.3443397855	2.5888163249	-1.7543421596
O	-1.4642797897	0.3850700569	-1.5407344433
O	-1.5256678824	1.7865007671	0.3129378981
H	-2.3934596817	0.1833708659	-1.3380453848
H	4.1559283553	3.4505586948	-2.3936176313
H	5.0147460286	2.8609712868	-0.936105793
H	4.7840378557	1.7737198304	-2.3337746386
H	0.6968889784	1.7206835488	-1.6354596852

C -1.7059183631 2.9966813968 -0.4284801266
H -2.3811823946 3.6172524218 0.1647730145
H -0.7595461097 3.5338387116 -0.5642964221
H -2.1572850551 2.8089452972 -1.4080593904

17a_t1
C 1.7956272899 -0.809391473 1.1699670357
N 2.4288578082 -1.6136961215 1.9289061118
C 3.7672631539 -1.000808914 2.1275044014
C 3.8044281839 0.3485667821 1.3251331407
N 2.4574018772 0.342678178 0.7565213557
H 4.5394998686 -1.6926558543 1.7782424054
H 3.9334032713 -0.8309029226 3.1954622608
H 4.5571972392 0.3535715742 0.5337788659
H 3.9471420011 1.2219445016 1.9652889855
N 0.5231170907 -0.8054387511 0.630207461
C -0.487511341 -1.7946812185 1.0568415413
C -1.4298410709 -1.2815800128 2.0932095547
H -1.0579567026 -2.0992711315 0.1824967422
H 0.0791302104 -2.6482644698 1.4363949788
O -0.9268971944 -1.0683251663 3.3516320474
C -1.9589059397 -0.626775564 4.1280448933
C -3.1063953712 -0.5590421603 3.3994856096
C -2.7612955083 -0.9829884353 2.0732200233
H -3.4116600684 -1.0579522888 1.2140018952
H -4.0772240001 -0.2461471856 3.7575220589
H -1.7167395879 -0.4105915286 5.1580268313
C 0.3614650873 0.3522793829 -0.1266294552
C -0.6461578458 0.847580662 -0.8842815009
C 1.66649237 1.131421614 -0.0092036599
C -1.9143897166 0.2268101083 -1.2776738578
O 1.952552886 2.2157921584 -0.5090905064
O -2.7425677244 1.1657570746 -1.7853096988
O -2.2156758423 -0.959171054 -1.2278297456
C -4.0082916673 0.6896791745 -2.2808414325
H -3.8608577291 -0.0544341398 -3.0675220155
H -4.6001185445 0.2504854329 -1.4737116015
H -4.5139509487 1.5681176543 -2.681673483
H -0.4740055093 1.8398991521 -1.2894879239
H 0.9644359713 3.4024828056 -1.5399514505
O 0.4137878789 3.9024288931 -2.1701655669
C 0.8971941264 3.5934007754 -3.4699633306
H 0.2631449069 4.1136513315 -4.1951339539
H 1.9332839176 3.9309474011 -3.6241151837
H 0.8532651739 2.5171967351 -3.6937778332

E-**17a_t1**
C 1.7861939424 -0.7048588824 1.1237801919
N 2.304285428 -1.2351333954 2.1684585982
C 3.7704205521 -1.0219262626 2.0431197196

C 4.0283566857 -0.341491672 0.654387645
N 2.6514394938 -0.1308312496 0.2107612976
H 4.2888486773 -1.9813634025 2.1212409902
H 4.1154944997 -0.3879935977 2.8657820834
H 4.560741813 -0.992642637 -0.0439440628
H 4.5610063455 0.6078780468 0.7382752537
N 0.5048550439 -0.5722842819 0.6475058344
C -0.6799070218 -1.08200722 1.3294132503
C -1.5687146244 0.0075396365 1.8559670879
H -1.2485270276 -1.7132753199 0.6377024245
H -0.330043216 -1.7260496966 2.1441362243
O -2.8890576588 -0.3304439659 1.9758396753
C -3.5358901282 0.7501149896 2.5102872996
C -2.6563719978 1.764319555 2.7253202902
C -1.3715388439 1.2809640178 2.3019098575
H -0.4335744655 1.8170780482 2.3263985476
H -2.8861289821 2.7391210145 3.1325590434
H -4.5979010901 0.6345086158 2.6669738331
C 0.5353133181 0.0567944802 -0.6060378327
C -0.5674380935 0.2784860606 -1.3549387856
C 2.0099262717 0.317346152 -0.9164031648
C -0.6488637074 0.9887091808 -2.6441515577
O 2.5275187866 0.7717350874 -1.9183489517
O -1.7849590096 0.605500986 -3.2815255789
O 0.1131331112 1.8227406445 -3.094757425
C -2.0392040998 1.2538322054 -4.540590499
H -2.1348157773 2.3349220395 -4.4083218255
H -1.2358034537 1.0485398525 -5.252849319
H -2.978204674 0.8349092884 -4.9029738836
H -1.5178596031 -0.0932209588 -0.9886488929
H 1.3676913547 -2.2349090351 3.4424696285
O 0.7565969762 -2.8121670559 3.9517468145
C 1.077837198 -4.1520217116 3.6139563406
H 0.3508293286 -4.8089656877 4.1033141688
H 1.0273597444 -4.3424053876 2.5311169541
H 2.0810109036 -4.4456474812 3.9609017252

17a_t2
C 0.6337205887 -1.0824383562 0.6384641725
N 0.6215243802 -2.2486106883 1.1491266072
C 1.1934971537 -3.1314158627 0.0994466962
C 1.6718464206 -2.2243953689 -1.0820862069
N 1.1395988207 -0.9233203027 -0.648398904
H 0.4255181309 -3.8402606992 -0.2274892496
H 2.0206822375 -3.7104754522 0.5192347293
H 1.2578849838 -2.5137515418 -2.0459669355
H 2.7601397968 -2.1786997312 -1.1613344284
N 0.2529698269 0.1608271007 1.1157947447
C -0.3572895731 0.4178741789 2.4316903937
C -1.8437482864 0.5299541568 2.3942108301

H	-0.0376857723	-0.3961277125	3.0865128009
H	0.0543733568	1.3550645925	2.8109999003
O	-2.5597097541	-0.6266405284	2.2237845617
C	-3.8808540608	-0.2831195997	2.2267289474
C	-4.02249315	1.0596123199	2.3993498457
C	-2.6943308224	1.5908642726	2.5076252624
H	-2.3967346807	2.6206756118	2.6495802491
H	-4.9541450321	1.6060394802	2.4454988504
H	-4.5806828286	-1.0960479282	2.1006807126
C	1.1601604071	0.39255581	-1.0116956161
C	1.5754304181	1.0769116132	-2.1069364925
C	0.5488597647	1.1208846589	0.1690121853
C	2.1379351106	0.4993536063	-3.3237268271
O	0.3608777163	2.3262602527	0.2658124271
O	2.5053433666	1.4798734227	-4.1828993068
O	2.2667126318	-0.6899127206	-3.5814923342
C	3.0596663328	1.0379474602	-5.4349986481
H	3.2905449539	1.946728064	-5.991249555
H	3.9696827436	0.4543394876	-5.2726902395
H	2.3370554863	0.4315282279	-5.9874264657
H	1.4601312613	2.1534199112	-2.0673500634
H	-0.0882989193	3.3710084656	1.771000608
O	-0.2491113896	3.7972945649	2.6317380405
C	0.7010981436	4.8437431385	2.7830582159
H	0.5522995048	5.2884755598	3.7718728655
H	1.7378101855	4.4813900526	2.7253713737
H	0.573786546	5.6369114829	2.0314342523

E-**17a_t2**

C	0.484746677	-1.2809156102	0.5720035115
N	0.2048855477	-2.5108999248	0.7615487895
C	0.6322370992	-3.1949497024	-0.4887809665
C	1.2370694761	-2.1125165998	-1.4506496379
N	1.0605238078	-0.9163929931	-0.6306047839
H	-0.2310133671	-3.690895877	-0.9424606473
H	1.3684894496	-3.96688948	-0.2462637308
H	0.6884527681	-2.023471935	-2.3923758414
H	2.2958039641	-2.2796527804	-1.668936047
N	0.3544556699	-0.1371166505	1.3350819837
C	-0.2049298378	-0.0678884788	2.6935707743
C	-1.6320589155	0.3636644789	2.741493477
H	-0.0817271203	-1.0590703769	3.1358492216
H	0.4027494684	0.6401915918	3.2594587012
O	-2.5830481116	-0.5852624643	2.465713977
C	-3.7986083363	0.0273608984	2.5630457978
C	-3.6448491538	1.3385092336	2.8954482826
C	-2.2320450219	1.559260749	3.0108963938
H	-1.7171625525	2.4818487563	3.248528695
H	-4.4363679664	2.0600396212	3.0426937781
H	-4.6591117817	-0.597478754	2.3747083929

C 1.3408233811 0.4199843292 -0.7032800371
C 1.9293699495 1.0234704588 -1.7706402793
C 0.8601532863 0.9571969765 0.6413189979
C 2.2555824864 2.4382801047 -1.9555492019
O 0.904226825 2.0940109475 1.0754276447
O 2.8939810256 2.5834337696 -3.1516730683
O 2.0248130142 3.3825577706 -1.2200778633
C 3.278566324 3.9258968847 -3.4876013974
H 3.7625346761 3.8592347451 -4.4625652109
H 2.4044775381 4.5802090351 -3.5467084657
H 3.9771807018 4.3290748023 -2.749198669
H 2.1952072864 0.3792262739 -2.6023705804
H 0.3772502382 3.3140351335 2.4369113131
O 0.0413156288 4.0571672059 2.9665331998
C -0.4162976075 5.034876924 2.0409285284
H -0.7709138 5.8967053302 2.6150206507
H 0.3832248302 5.3787142662 1.3686680508
H -1.2502155473 4.6732533402 1.4212912669

18a_t1
C 0.473886546 0.4585830677 2.2532550421
N 0.9418124866 0.0223463581 3.3649479199
C 0.5787975707 1.0120546124 4.3986550786
C -0.341968651 2.0625525328 3.7194991335
N -0.2451809532 1.6559115761 2.3074425412
H 1.4896927937 1.476084409 4.7942349342
H 0.0751711505 0.517552466 5.234586132
H -0.0116888242 3.0952416971 3.8426881025
H -1.3839335866 1.989896834 4.0460346217
N 0.6170236113 -0.1524534542 1.0140323851
C 1.2856668315 -1.4823877526 0.9763202864
C 0.3644503482 -2.6026525079 0.6361936941
H 2.0946974517 -1.4489556634 0.2494112236
H 1.7092892563 -1.6148821708 1.9721991315
O -0.6081497523 -2.9159266772 1.5505652966
C -1.3021394703 -3.9799967806 1.0517912214
C -0.7930215787 -4.3576941763 -0.1523503176
C 0.2907019257 -3.4584279933 -0.4252758115
H 0.9337406097 -3.448214527 -1.2937640972
H -1.1436739677 -5.1707559807 -0.7723885449
H -2.1147135007 -4.3425838303 1.663782566
C 0.0453515092 0.441964194 -0.0955038126
C -0.6745832927 1.5920114819 -0.0206745012
C 0.3493998235 -0.1356226868 -1.4555382293
C -0.8637546353 2.2752589554 1.2497578634
O -0.7204169771 -0.0830535365 -2.2493609099
O 1.4477003308 -0.5340129924 -1.7912587537
C -0.5131692727 -0.5057074986 -3.6195710499
O -1.5162063667 3.3206473583 1.3861132965
H -1.4780385034 -0.3780582501 -4.1078254992

H 0.2447647009 0.117733422 -4.0982155439
H -0.2054551522 -1.5529246308 -3.6478146583
H -1.0983362593 2.0395541064 -0.9080690881
H -1.2233376429 4.6411835325 2.6372229956
O -0.9163574947 5.3600534902 3.2233870378
C -0.0500507257 6.175429654 2.4507965855
H 0.3475665429 6.9586378086 3.1049785704
H 0.8041924327 5.6180393087 2.0370852737
H -0.5675963141 6.6689512442 1.6134138841

18a_t2

C 0.017924878 -0.6750117522 1.4504349456
N 0.1860195191 -1.599490144 2.3228553698
C 1.6206099635 -1.6167684582 2.6554987981
C 2.3217139564 -0.5794560854 1.735984971
N 1.1610430515 0.0172171469 1.0351772385
H 2.0298263099 -2.6205683683 2.5034366547
H 1.7562893212 -1.3668678237 3.7138936423
H 2.9963379665 -1.038361485 1.0126214154
H 2.8678246877 0.1866885997 2.2871264516
N -1.1991106326 -0.306379664 0.8944433234
C -2.4240541436 -1.0247033119 1.3157634938
C -2.8729225261 -2.0732442813 0.354215664
H -2.2098561986 -1.4614184328 2.2913841893
H -3.2127441433 -0.2799316989 1.4172214053
O -2.2360205652 -3.2876422098 0.4005383219
C -2.8122697799 -4.0816761885 -0.5476801689
C -3.8024012667 -3.407447165 -1.1943379382
C -3.8401925074 -2.0991584117 -0.6084521368
H -4.4959696401 -1.2806332171 -0.868583252
H -4.4285263566 -3.7904740558 -1.9880766213
H -2.4144458577 -5.0821912631 -0.6320430531
C 1.096422545 1.0466504026 0.1428569409
C -0.1028162407 1.4304999178 -0.3799149192
C 2.3833125152 1.7093186578 -0.2694108708
C -1.3236579862 0.7473921534 -0.011677432
O 2.1541330448 2.8589139187 -0.907336183
O 3.4866207466 1.2459973958 -0.0569292755
C 3.3259301421 3.5560406159 -1.392013469
O -2.4445338459 1.0510288977 -0.4502124054
H 2.946496772 4.4574832646 -1.8707020312
H 3.9845045963 3.8121652755 -0.5594846899
H 3.8637913128 2.9368242859 -2.1131567653
H -0.1802062266 2.2411668518 -1.0892538525
H -2.3979990954 2.4580286257 -1.6216374175
O -2.2304781758 3.2701567119 -2.1415598839
C -2.619501205 4.3640393363 -1.3262345074
H -2.400226202 5.2873311406 -1.8729504596
H -3.6964130469 4.3584673362 -1.0963167854
H -2.071535686 4.4007674821 -0.3722617075

S7.3. B3LYP Optimized geometries (from 1b tautomers)

1b_t1

C 0.038522206 0.859363814 0.0191778037
N -0.1386724416 1.9107697718 0.8936201726
N 1.1213767908 0.6082940438 -0.6539882001
C -1.4542177983 2.3469764255 1.3750658068
H 0.5490914016 2.6468507902 0.8179925334
C -2.3704914887 1.1421465004 1.5617497256
H -1.3103802082 2.8790595665 2.3204990489
H -1.9136640632 3.0529265981 0.6672531895
C -2.3775764854 0.3134846439 0.2804960998
H -2.0133443551 0.5261187135 2.3947950989
H -3.3827156505 1.4821304591 1.7994103778
N -1.0024183577 -0.0505866833 -0.0531023362
H -2.9492225033 -0.6094633415 0.4153423083
H -2.8605406357 0.884659202 -0.5285889198
H -0.8790721664 -0.7109037038 -0.8099303852
C 2.2517821904 1.5117735893 -0.4685111069
H 2.034942639 2.543352884 -0.7942287037
H 2.5475145191 1.5880176645 0.593522317
C 3.4417300577 1.0636317442 -1.2543484503
C 4.1254806416 1.5983265006 -2.3073392657
C 5.1967104935 0.6903961837 -2.6113197433
C 5.0863081642 -0.3315608234 -1.7204115684
O 4.025351422 -0.1212155184 -0.8849323767
H 5.6491628775 -1.237910343 -1.5523983714
H 5.9432743455 0.7889181177 -3.3873362688
H 3.8921734051 2.5280012009 -2.8082107859

1b_t2

C -0.9048163869 0.0227717424 -0.4005870547
N -1.5023260037 0.1502271863 0.7381154307
C -1.0851754345 1.2571995962 1.6030021729
N 0.0107797254 0.9041724583 -0.9552622081
H 0.6315797388 0.5018735092 -1.6471579202
N -1.1675146331 -1.073083983 -1.216715549
C -2.302064283 -1.9434933355 -0.9086795801
C -2.2781044927 -3.1836514959 -1.7355916535
H -2.2476428703 -2.1908088787 0.1551582228
H -3.2692272981 -1.4467993368 -1.0700224237
O -1.2655211433 -4.0798341461 -1.5033289088
C -1.4519259211 -5.1309177206 -2.3551248173
C -2.5571477009 -4.9299135782 -3.1227465979
C -3.093990828 -3.6606812906 -2.7201013496
H -3.9728353356 -3.1660899245 -3.11041076
H -2.9438999032 -5.5974337552 -3.8802104798
H -0.7244187168 -5.926535545 -2.2912174624
H -1.9597528689 1.5916878348 2.1755786829

H	-0.3633375288	0.8871970305	2.3485110089
C	-0.4731775554	2.4463073871	0.8533670609
C	0.5991318089	1.9413665292	-0.1085472724
H	-0.039490037	3.1661467918	1.5553623426
H	-1.2530857171	2.9665150698	0.2839160199
H	-1.0574026257	-0.8857236272	-2.207602095
H	0.9666601961	2.7469130462	-0.7522698914
H	1.4589078151	1.5544514354	0.459357082

NCov(1b_t1)

C	-1.1421325658	1.1953121045	1.661820871
N	-1.5710510027	2.5039329335	1.7170464463
N	0.0252180194	0.799841223	1.2435306124
C	-2.9756218493	2.8889157111	1.8914737057
H	-1.0131892795	3.1612977398	1.190833202
C	-3.6826615721	1.8931655942	2.8050938098
H	-3.0013733941	3.8970832448	2.3165659803
H	-3.4916024801	2.9284503424	0.9205879034
C	-3.4399071415	0.4787375749	2.2868183976
H	-3.2947025096	1.9811991112	3.8262820552
H	-4.7543670464	2.1114762597	2.8282718175
N	-2.0003294235	0.2481244863	2.1913067658
H	-3.8575508853	-0.2656613314	2.9712469115
H	-3.9428993985	0.3495305029	1.3148142782
H	-1.7069641795	-0.6970787629	1.9698174887
C	0.9600524338	1.8237411635	0.7898396932
H	0.5886280462	2.3837189973	-0.0850968466
H	1.1528392354	2.580094064	1.5719155283
C	2.2690592653	1.2273017453	0.3853177529
C	2.90693422	1.0905774084	-0.813267679
C	4.1517012024	0.424429766	-0.5473449028
C	4.1815720514	0.2065380786	0.7952364478
O	3.0456147812	0.6922499512	1.3802321102
H	4.9009221306	-0.2473032225	1.4608114047
H	4.9167297427	0.1490597818	-1.2602609396
H	2.5315599357	1.4204672909	-1.7723141228
C	-0.5984547125	-1.6271559177	-2.1909891988
C	-0.3065878201	-1.9490175777	-1.0606209736
C	-0.9279174761	-1.3157635194	-3.5695518447
C	0.0089395951	-2.2776586406	0.3174011118
O	-1.0970457395	-0.0014637931	-3.7376599141
O	-1.0274438552	-2.1637375201	-4.4342321875
C	-1.4259252072	0.4207546924	-5.0849421186
O	1.3234375263	-2.3421903039	0.5068196721
O	-0.8477456206	-2.4946541446	1.1566090103
C	1.7508311612	-2.5782611793	1.8710806839
H	-1.5292616067	1.5032550389	-5.0276250066
H	-0.6230872058	0.1482738132	-5.7732409928
H	-2.3633761721	-0.0374970475	-5.4069527517
H	2.8376512758	-2.6210897409	1.8261941548

H 1.4231711958 -1.7531777843 2.5051626594
H 1.3442143256 -3.5227641338 2.2382670043

NCov(1b_t2)

C 0.0034886299 2.2549301685 0.6202572978
N -0.0018345945 3.3511795176 1.4671705108
N 1.2598403844 1.8243001287 0.2135356874
C -1.2690641426 4.0179991957 1.7750369447
H 0.7915056582 3.9730183179 1.3800876357
C -2.3855440329 2.9789036268 1.804419187
H -1.1693560274 4.5225670574 2.741831978
H -1.4989220749 4.7886802109 1.024376956
C -2.3532824862 2.1699474974 0.5036730184
H -2.2384377124 2.3076414287 2.6594513375
H -3.3526714427 3.475727975 1.9355080461
N -1.0363245129 1.6117218497 0.1923802952
H -3.067976623 1.3397266361 0.5610113898
H -2.6994319145 2.8068875938 -0.3273549572
C 2.4744211237 1.937274285 1.0291096221
H 3.2685780105 2.447115993 0.4711755927
H 2.2518944089 2.5549384302 1.9043512637
C 3.0258429869 0.6212050044 1.4741420178
C 4.216264695 -0.0076377478 1.2506663743
C 4.1666924994 -1.2508686345 1.9678341573
C 2.9465512917 -1.2891396025 2.5704277911
O 2.2398493484 -0.1556393305 2.2859567698
H 2.4512651747 -2.0087053031 3.2055088158
H 4.9355626347 -2.0088725797 2.0250858058
H 5.0292787806 0.3691818763 0.6455158087
H 1.1993554868 0.9808144738 -0.3495302615
C -1.9502684248 -1.567521675 -1.4956561711
C -0.7515917205 -1.7342893717 -1.4602077904
C -3.3871552741 -1.3674569031 -1.4409917294
C 0.6920001319 -1.8579537848 -1.4275966169
O 1.0681603315 -3.1344280849 -1.4684893674
O 1.4427492269 -0.8994401507 -1.3744986182
C 2.4984912079 -3.3763648926 -1.4245029256
O -3.9162054349 -1.3423081229 -2.6654628214
O -3.996703759 -1.2450204845 -0.3963886715
C -5.3515829299 -1.1442875345 -2.7259946883
H 2.6048051567 -4.4586660519 -1.4779670294
H 2.9881813706 -2.8990722875 -2.2755378456
H 2.9142464382 -2.9936953738 -0.4901561927
H -5.5969295621 -1.1581905295 -3.7867720095
H -5.8666081446 -1.9520765838 -2.2021847662
H -5.6186021635 -0.1827632376 -2.2825818412

TS1(1b_t1)

C -1.2895012539 0.8737918454 0.6255055582
N -1.3565141538 2.223696989 0.8341022945

N	-0.3778000965	0.298102403	-0.1356659553
C	-2.4041251951	2.8915623985	1.610573718
H	-0.8522795853	2.8027959294	0.1790687441
C	-2.923330136	1.9571093982	2.6978714138
H	-1.9778767667	3.8008216875	2.0449970988
H	-3.2317803035	3.1946417322	0.9538691028
C	-3.3070083409	0.6213273817	2.0671518534
H	-2.1483619908	1.7983551394	3.4560873496
H	-3.7907741503	2.4085374355	3.1876682216
N	-2.1846312098	0.0974427794	1.2960635961
H	-3.558431082	-0.1167493629	2.8334751826
H	-4.1958940882	0.7507918064	1.4319059737
H	-2.0682150338	-0.908533606	1.2369141031
C	0.6261653031	1.1495848944	-0.7620073317
H	0.2041885608	1.7830209556	-1.5561091926
H	1.0762353912	1.8288068823	-0.0183435644
C	1.7213935845	0.3469589522	-1.38478785
C	2.1755135735	0.2214802485	-2.6661839739
C	3.2942223185	-0.6783372872	-2.6230418142
C	3.438564589	-1.0350844069	-1.3183158544
O	2.4925688497	-0.4184170517	-0.5489184618
H	4.1244715236	-1.6847141223	-0.794933247
H	3.9022522843	-1.0127466638	-3.4522409133
H	1.7619767859	0.7130020466	-3.5359133599
C	-1.0668096275	-1.4424487985	-1.3445989926
C	-1.1145437669	-2.5182334273	-0.7219019603
C	-1.3084568095	-0.7651730538	-2.6265729812
C	-1.0583184188	-3.1887447882	0.540470874
O	-1.2040962766	-1.6604451641	-3.6276134326
O	-1.5929486685	0.4038982433	-2.7910404049
C	-1.4900391154	-1.1489894069	-4.9480913466
O	-0.0985896305	-4.1274701568	0.5909672096
O	-1.8220090587	-2.9408337713	1.4724044335
C	0.0420355951	-4.8162607848	1.8524917605
H	-1.374670663	-2.0005949668	-5.6180520144
H	-2.5102303504	-0.761086867	-4.9981937888
H	-0.7845991767	-0.3582549523	-5.2141922236
H	0.8696925198	-5.5109514891	1.7114938998
H	0.2727523617	-4.1109567936	2.6544243386
H	-0.8725462919	-5.3618452273	2.0966699373

TS1(1b_t2)

C	-0.2761246797	1.1111999025	-0.0983792503
N	-0.3164608107	2.366800656	0.4435633766
N	0.9497473658	0.6517459258	-0.5009863816
C	-1.5864919466	2.9420384731	0.8989663609
H	0.5189938513	2.6980954525	0.9056808779
C	-2.5212981332	1.8277545018	1.3606505554
H	-1.3633619618	3.6461711571	1.7032583076
H	-2.0503746891	3.5081509899	0.0810821823

C	-2.6402544678	0.7903689379	0.240895721
H	-2.1155425553	1.3631221643	2.2669794038
H	-3.5089775956	2.2323338618	1.6033732238
N	-1.3427838585	0.3658828741	-0.2846619429
H	-3.1674376693	-0.0996381195	0.604892846
H	-3.251827098	1.1996525946	-0.574020219
C	2.1792698307	1.4420347293	-0.3943228627
H	1.9808562511	2.4525962814	-0.7618943934
H	2.5189830159	1.5302047445	0.6488301047
C	3.2815352349	0.8624665046	-1.2146232283
C	3.7660994295	1.1302695163	-2.4617234011
C	4.8414943093	0.2089590683	-2.6911841338
C	4.9320784125	-0.5528732428	-1.5666843947
O	3.9924193667	-0.168278414	-0.6552598598
H	5.578909482	-1.3637788946	-1.2663536584
H	5.4625884732	0.1300738057	-3.5725246981
H	3.4001607839	1.8935409135	-3.1345376894
H	1.0607290018	-0.3594921619	-0.5398431569
C	-1.2794752276	-1.1449046305	-1.760117026
C	-0.674056886	-2.2097120093	-1.5184170911
C	-2.0895644297	-0.4008185274	-2.7323686809
C	0.1900419847	-2.843157368	-0.5707021991
O	-0.3213203869	-3.9811780784	-0.0634266673
O	1.2997036093	-2.4061206687	-0.2662226146
C	0.4922793981	-4.6503799102	0.9218843356
O	-1.3254108383	0.0847603075	-3.7238778428
O	-3.2954889876	-0.2554261913	-2.6604109368
C	-2.0290656892	0.8168069988	-4.7530204994
H	-0.079516602	-5.5282802523	1.2226911475
H	1.4500430314	-4.9534425128	0.4918227011
H	0.6697864905	-4.0027132503	1.7840896899
H	-1.2626382115	1.1278319898	-5.4622149088
H	-2.7646780772	0.1762139296	-5.2448797276
H	-2.5299205209	1.6894709512	-4.3272773693

10b_t1

C	-1.4614505447	0.9000810117	0.3447911316
N	-1.3643984735	2.1886319131	0.7100428676
N	-0.6275727218	0.3722046055	-0.5874977299
C	-2.1037384179	2.8033807444	1.8231242881
H	-0.8945049246	2.8250356479	0.0818343111
C	-2.685780449	1.730794574	2.7362559109
H	-1.4158287303	3.4569065381	2.367180607
H	-2.904395139	3.4268691063	1.4090351836
C	-3.3525096645	0.6562347203	1.8806641311
H	-1.8949622995	1.2752495945	3.3418227002
H	-3.4106564927	2.188657616	3.4145960173
N	-2.3880664834	0.1175877781	0.9154428711
H	-3.7019625946	-0.1809661396	2.4871985043
H	-4.2182471278	1.0708907121	1.3500885832

H	-2.1923698473	-0.8926158387	0.9458599772
C	0.6316439591	1.0368083036	-0.9851279323
H	0.543480692	1.4021263341	-2.0115283048
H	0.8014227841	1.8978896692	-0.3353001161
C	1.8101861647	0.1271483184	-0.8929697687
C	2.6585193768	-0.3993211788	-1.8223138515
C	3.6335095618	-1.1659622639	-1.1002202692
C	3.3017702183	-1.0530424937	0.2148066418
O	2.1984544359	-0.2634115732	0.3624099237
H	3.7278391531	-1.4461203717	1.1258633976
H	4.4628745529	-1.7264761457	-1.508565784
H	2.5954603863	-0.2568590931	-2.8923164829
C	-1.0776224604	-0.7524184765	-1.4322115518
C	-1.0307278958	-2.0469811277	-1.1008367018
C	-1.6073898815	-0.2458935914	-2.7312989947
C	-0.6536307775	-2.5457229088	0.17582222
O	-1.9616918221	-1.2296561089	-3.5846686791
O	-1.7172735523	0.9391734317	-3.0201307155
C	-2.4872308597	-0.7902718829	-4.8486163078
O	0.5425909178	-3.1960740911	0.1895787017
O	-1.3882931714	-2.527541286	1.1849498346
C	0.9398662121	-3.7556157474	1.4497573747
H	-2.7145213397	-1.7008226717	-5.4041786254
H	-3.3962073587	-0.1981550431	-4.7115429251
H	-1.7506525133	-0.1929670534	-5.3928965033
H	1.9308279335	-4.180824055	1.2834079778
H	0.9914657738	-2.9890935355	2.228164678
H	0.2497324205	-4.5415769412	1.7705314096

10b_t2

C	0.1783441726	1.1538685648	-0.1774002736
N	-0.0061303519	2.3415528091	0.4239502315
N	1.3853079612	0.8491052023	-0.7033243182
C	-1.3117582838	2.7643292382	0.9401106777
H	0.8113018026	2.8379262411	0.7510458071
C	-2.0315249912	1.5443768332	1.5024899071
H	-1.1299615305	3.520567541	1.7049503886
H	-1.8966690369	3.2252429509	0.1357222962
C	-2.1232002839	0.4759074178	0.4192668445
H	-1.4813358341	1.1672593908	2.3712110672
H	-3.0406365603	1.8088972316	1.8303064894
N	-0.8381682315	0.279163125	-0.2874824341
H	-2.4058817582	-0.4895317012	0.8489903489
H	-2.8763307112	0.7517781254	-0.3253197337
C	2.4982662355	1.81036725	-0.7268768158
H	2.1144525881	2.78250823	-1.0458647457
H	2.9351096911	1.9325464569	0.2742634428
C	3.5589660593	1.3967262359	-1.6877669481
C	3.8844878685	1.7940583881	-2.9514791734
C	5.0184903514	1.0102419468	-3.3479842034

C 5.298457232 0.1921821137 -2.2968004276
O 4.4220932627 0.414463981 -1.2744264135
H 6.0493655543 -0.5635291372 -2.1203068948
H 5.5516310769 1.0544402153 -4.2873700491
H 3.376912534 2.5545784049 -3.5286198578
H 1.6416860629 -0.1458906854 -0.6794605021
C -0.8129488576 -0.8002030706 -1.2916095393
C -0.1977695608 -1.978439024 -1.1409505637
C -1.5662888761 -0.3999241191 -2.5146199001
C 0.6533752618 -2.2964947161 -0.0451407353
O 0.1217681821 -3.1819784155 0.8445266923
O 1.8351078764 -1.9140457691 0.0518218603
C 0.9999285216 -3.6190519853 1.8934135508
O -1.6724027794 -1.3926536787 -3.4229719139
O -2.0470352281 0.710270029 -2.7015944581
C -2.3742307625 -1.0510776783 -4.630282359
H 0.4066447841 -4.2950643396 2.5113936301
H 1.8660951284 -4.1514327541 1.489724511
H 1.350839208 -2.7772685361 2.4973388362
H -2.3641571679 -1.9546368096 -5.2408956641
H -3.40465566 -0.755929023 -4.4141742308
H -1.8724499497 -0.2359864799 -5.1588864265

TS2(1b_t1)
C -1.2382834117 1.0321196426 0.5462963088
N -1.2744321696 2.3250972962 0.9226964224
N -0.3855853153 0.6321771706 -0.4273786217
C -2.1209481372 2.8525197741 2.0023078092
H -0.8729151588 3.0106924876 0.3002632353
C -2.4967686204 1.7390938518 2.9715257725
H -1.5610748911 3.6445176031 2.5064327177
H -3.0228327709 3.3000636059 1.5678706556
C -3.0214539665 0.5453355281 2.1810620043
H -1.6230056982 1.4371409656 3.5586753048
H -3.262219876 2.1015170562 3.6624587645
N -2.0381580907 0.1519508634 1.1671070408
H -3.1820622792 -0.3211789875 2.8252039532
H -3.9772605713 0.791642811 1.702068108
H -1.8901228704 -0.838389887 0.9967602778
C 0.6055503744 1.5768083996 -0.9981392853
H 0.1592446453 2.1483356685 -1.8164284295
H 0.9085694478 2.2721646212 -0.2097840914
C 1.8237607084 0.8909539368 -1.5100837916
C 2.3234597913 0.6881943797 -2.7635058767
C 3.5713079485 -0.0004116562 -2.6005442359
C 3.7379080889 -0.1661339801 -1.2599579518
O 2.6868092112 0.3750794614 -0.5782881106
H 4.5086440802 -0.6219789689 -0.6561931569
H 4.2480934331 -0.3268442833 -3.3776948165
H 1.8542317558 0.989491231 -3.6893942332

C -0.5190683773 -0.7180037838 -1.0040808774
C -0.3891820982 -1.7968871844 -0.267183668
C -0.9251276146 -0.656907385 -2.4429667312
C -0.6239291451 -2.9505014633 0.4229569376
O -0.8910084723 -1.8639944219 -3.0395029464
O -1.2593197437 0.3635080477 -3.0281178275
C -1.2978391417 -1.8837860576 -4.4193226549
O 0.4482756514 -3.8243917049 0.4971783521
O -1.7103341216 -3.1952509084 1.0074950905
C 0.2653273717 -4.9287835402 1.3857282783
H -1.2050889168 -2.92247158 -4.73771463
H -2.3331735773 -1.5491389716 -4.5261223901
H -0.649850562 -1.2452277338 -5.0256617911
H 1.1878743544 -5.5110452505 1.3281447835
H 0.1079510426 -4.5993937135 2.4188466723
H -0.5805043069 -5.5565559401 1.0884886287

TS2(1b_t2)

C -0.0581541444 1.4873370642 -0.1990768866
N -0.1345368593 2.6912107539 0.3954171789
N 1.1425353533 1.0552557477 -0.632583044
C -1.3755969263 3.2787723268 0.9057044781
H 0.724601447 3.1902433136 0.5723816235
C -2.3178788643 2.1569003932 1.3177238225
H -1.1117569103 3.9162247499 1.7519153104
H -1.8365949355 3.9089156878 0.1354641363
C -2.4772158598 1.1879133838 0.1537083914
H -1.9162316099 1.6382530277 2.1948201415
H -3.2964936018 2.5658905609 1.5829022241
N -1.1658370813 0.7373410102 -0.3635886958
H -3.0186537509 0.2917872777 0.4671314043
H -3.0374804381 1.6539408906 -0.6617961002
C 2.382629942 1.8406465727 -0.5228680257
H 2.2731172914 2.7796918427 -1.0750642892
H 2.5874491014 2.0862318405 0.5278220331
C 3.5419933324 1.0930825427 -1.0803295149
C 4.1770620892 1.1085512736 -2.2877032743
C 5.2227988484 0.1305494143 -2.2107948365
C 5.1446165299 -0.4077021174 -0.9625037089
O 4.1312829084 0.1689602236 -0.2574209686
H 5.7118902997 -1.1653981574 -0.442579926
H 5.9347067717 -0.1319333158 -2.9807374099
H 3.9272835122 1.7399143642 -3.129173157
H 1.2227941318 0.0803139675 -0.9036428007
C -1.1148696311 -0.5435387696 -1.0908949766
C -0.3165159587 -1.5246068318 -0.742275159
C -2.0042630547 -0.5187514878 -2.2967747919
C 0.6803485347 -2.44864765 -0.6186140497
O 0.3483768538 -3.5554395065 0.1516741699
O 1.8242458204 -2.3209188522 -1.1192910582

C 1.4247989216 -4.4619476203 0.3965330958
O -2.1895069986 -1.7454171565 -2.8231903437
O -2.5052793764 0.4887445143 -2.7744221865
C -2.9962012548 -1.7916126254 -4.0134722224
H 1.006507417 -5.2577248634 1.0174281959
H 1.8160450685 -4.8928233274 -0.5308360909
H 2.2512145791 -3.9808552508 0.931193236
H -3.0408034587 -2.8435366639 -4.29704468
H -4.0026184137 -1.4120435017 -3.817436746
H -2.5419186253 -1.2050670463 -4.8165254987

11b_t1
C -1.3577531111 1.1545754262 0.59341402
N -1.4254541594 2.4871272146 0.7951194453
N -0.6546754952 0.6576510877 -0.460699409
C -2.1305667221 3.0970038405 1.9322770372
H -1.260704606 3.0902817506 0.0014817288
C -2.2457076681 2.1046641767 3.0846198895
H -1.5728639455 3.9876059646 2.2342343163
H -3.1276186515 3.4177377992 1.6072569889
C -2.7820109785 0.7753234002 2.5582848569
H -1.264784868 1.9462688102 3.5453492654
H -2.9157589004 2.5119767525 3.8462683508
N -1.9448657927 0.3205298886 1.4488397731
H -2.7528267922 0.0007834126 3.3275917729
H -3.8233094299 0.8828914823 2.2293208639
H -1.6734894288 -0.6742365913 1.300575019
C 0.3585058893 1.5062357243 -1.1391515547
H -0.0383309509 1.8962436441 -2.078377682
H 0.5846449364 2.3485868677 -0.4814613765
C 1.6228233604 0.7718038749 -1.4282968046
C 2.2531640266 0.4215665012 -2.5869343256
C 3.4741437934 -0.2301292789 -2.2085739023
C 3.4950692005 -0.2300203998 -0.8478833396
O 2.3786576518 0.380584862 -0.3536137936
H 4.1940480596 -0.6035263012 -0.1142112573
H 4.2287407551 -0.6436471594 -2.8629426601
H 1.8875211246 0.6040323445 -3.5877115861
C -0.8178134342 -0.7229104658 -0.9127646326
C -0.9305780686 -1.7392504013 -0.0442805327
C -1.0011312159 -0.7877553069 -2.3957387473
C -1.2566585089 -3.0974081798 -0.3969865293
O -0.7636360621 -2.019193031 -2.889850091
O -1.3521593364 0.1476631079 -3.1012799633
C -1.0188018948 -2.187735903 -4.295147239
O -0.1468313613 -3.901516284 -0.4257427563
O -2.3907553912 -3.560658904 -0.5072373668
C -0.4047124031 -5.2966194796 -0.6258409839
H -0.7931619417 -3.232305515 -4.510952271
H -2.0643004906 -1.9720705472 -4.5307460128

H -0.3740459032 -1.533514093 -4.8878653532
H 0.5708708018 -5.7861346416 -0.6046105485
H -1.0397108094 -5.7043856173 0.1667136991
H -0.8870312776 -5.4800948333 -1.5909293082

11b_t2

C 0.128858743 1.7278970857 -0.2444147215
N -0.1129331184 2.9710983166 0.2175723051
N 1.385505506 1.3488285236 -0.5085422448
C -1.4421402751 3.4519986807 0.6003343662
H 0.6819589967 3.5098488248 0.5301429522
C -2.2615221489 2.2662656908 1.0879095008
H -1.3067117636 4.2030765921 1.3806051851
H -1.9298925597 3.9340765772 -0.2554847868
C -2.2642279162 1.1932169221 0.0089863411
H -1.8347897013 1.8797473476 2.0197957367
H -3.2928829027 2.5703740326 1.2864761635
N -0.8925671938 0.8608538831 -0.4552887854
H -2.7060128651 0.2686056521 0.3894831824
H -2.8564913582 1.5206558338 -0.8485174776
C 2.5589717822 2.2064451377 -0.3299264269
H 2.4202882196 3.1502045519 -0.8670938339
H 2.7013246837 2.4470447049 0.7342874564
C 3.7828737177 1.5500709832 -0.8676061268
C 4.6131685043 1.8279699113 -1.9124181441
C 5.6335497389 0.8184584128 -1.9034765709
C 5.3455194313 -0.0014995343 -0.8568038598
O 4.2227313819 0.4302353205 -0.2092573074
H 5.8109075451 -0.888959537 -0.4545986434
H 6.4650323924 0.7196746729 -2.5871709749
H 4.5094670783 2.6515662225 -2.6051311924
H 1.4688128331 0.3077117255 -0.5982349977
C -0.6963589392 -0.4542659552 -1.0576110854
C 0.4312167531 -1.1592814806 -0.8859667276
C -1.795651914 -0.8004049839 -2.0136005472
C 0.734612331 -2.3979318836 -1.5568168748
O 0.5584686752 -3.4805219143 -0.734733776
O 1.238179625 -2.512254163 -2.6728761035
C 0.9687389532 -4.7401063456 -1.2809202032
O -1.967339852 -2.13283989 -2.1305543719
O -2.4476779182 0.0079806389 -2.6577150853
C -2.8982764971 -2.556248501 -3.1427644512
H 0.7750684819 -5.4800956628 -0.5019978409
H 0.3960634052 -4.9925568953 -2.1786748659
H 2.03369805 -4.7392959966 -1.5327350868
H -2.9123530198 -3.6451235564 -3.0912456921
H -3.8961316617 -2.1567009686 -2.9443847084
H -2.5700632238 -2.2310739768 -4.1336036753

TS3(1b_t1)

C -1.3255978396 1.4004308479 0.960321656
N -1.624443195 2.7236400562 1.0239330166
N -0.4474537413 0.9709320765 -0.014220383
C -2.5696620024 3.2616123978 2.0113117402
H -1.5100224903 3.2675497563 0.17977159
C -2.6214324527 2.3571241294 3.2392023762
H -2.2422161813 4.2701751653 2.2792602544
H -3.5666542612 3.3419464595 1.5599489777
C -2.8300764871 0.9089822945 2.7959068803
H -1.6846672536 2.4354575902 3.8019597975
H -3.4350554947 2.6810227945 3.8943483734
N -1.8110006475 0.528122048 1.8196371054
H -2.7491303563 0.2227076545 3.6436085609
H -3.8334791791 0.7824380398 2.367280608
H -1.3225310573 -0.5432247267 1.5987964923
C 0.5724871076 1.9213435364 -0.5356695686
H 0.3703076792 2.1698007097 -1.5784106152
H 0.4965801622 2.8371828901 0.0529051608
C 1.9665576922 1.4014051295 -0.4356779293
C 2.9245389196 1.1160155335 -1.3651419895
C 4.0921373301 0.7065148186 -0.638461514
C 3.7572850792 0.7697905245 0.6788979377
O 2.4684449666 1.1963107321 0.8235653028
H 4.2896636537 0.5547923294 1.5935585058
H 5.0466048388 0.4033333785 -1.0454959445
H 2.8113424185 1.1912675158 -2.4376991514
C -0.2855122884 -0.4402713713 -0.2854460907
C -0.5902247313 -1.3675483811 0.6405674963
C 0.081959557 -0.8170359118 -1.6872901454
C -0.6249408156 -2.8003417368 0.3959998453
O -0.2708020762 0.116226376 -2.6000244051
O 0.5820855302 -1.8840488504 -1.9962186396
C 0.0102167137 -0.2175087704 -3.9740059461
O 0.4811245366 -3.4242130734 0.8912755254
O -1.5793769968 -3.4244124244 -0.0538278029
C 0.4769239798 -4.8563522344 0.7967745927
H -0.3107416809 0.6459100552 -4.556841027
H 1.0779587679 -0.3977912587 -4.1197233141
H -0.5503767146 -1.1058222395 -4.2754964244
H 1.4143448827 -5.1863792251 1.2475591025
H -0.3690601975 -5.2878866611 1.3396103856
H 0.432717325 -5.1834659743 -0.2462613931

TS3(1b_t2)

C -0.2994881478 1.8178358662 0.5909955296
N -0.4635404388 3.1063085381 0.9728886355
N 0.9049578185 1.3101987106 0.3689066314
C -1.737204071 3.6908962421 1.3943807523
H 0.3758411366 3.6227726074 1.1909406873
C -2.6265443806 2.5796074513 1.9291967625

H -1.5217818846 4.4406693491 2.158653803
H -2.2214339907 4.1974331878 0.5500304426
C -2.7338249302 1.485480827 0.8768385256
H -2.2079537404 2.1808936396 2.8597037572
H -3.6281213752 2.9627278469 2.1429615729
N -1.3996329599 1.0138946786 0.4264769556
H -3.2660178756 0.6228385052 1.2843872281
H -3.2982248061 1.8518953174 0.0152640678
C 2.1283742499 2.0971366844 0.496296636
H 2.1299323634 2.9416261951 -0.2048492684
H 2.2134065417 2.5182759161 1.5102527987
C 3.3329701095 1.2675867896 0.2060650025
C 4.2819460419 1.3066059603 -0.7721418409
C 5.2059763218 0.2445775192 -0.4898644616
C 4.748622534 -0.3640587756 0.6375322036
O 3.6094668966 0.2472508324 1.0794978982
H 5.0949261696 -1.2007411553 1.2260063853
H 6.0876464835 -0.026358701 -1.0537177475
H 4.3172448426 2.0065921906 -1.5954074081
H 0.8135142048 0.1467129954 0.2144927546
C -1.2722070848 -0.3537473092 -0.0181269139
C -0.0926784028 -0.9973904092 -0.0308791108
C -2.5018870163 -0.9808336367 -0.608812617
C 0.1047641177 -2.3259704585 -0.5822010784
O 0.3048809969 -3.2546787929 0.3979447549
O 0.2240340119 -2.6036064887 -1.7710454786
C 0.6163831447 -4.5785995731 -0.06010484
O -3.1782511785 -0.1350211964 -1.4166827563
O -2.8151674405 -2.1480693109 -0.4605236424
C -4.3414795966 -0.6856412868 -2.0681241081
H 0.7473969103 -5.1810537491 0.8403804406
H -0.196352481 -4.9902613127 -0.6658077944
H 1.5372821484 -4.5879631261 -0.6504835277
H -4.7590021963 0.1291368415 -2.659629197
H -4.0603830288 -1.5186173883 -2.716960596
H -5.0708350178 -1.0304620213 -1.3308738386

12b_t1
C -0.8735290925 1.1841783448 1.032493758
N -1.7886006581 2.1829382048 0.8460702205
N -0.2756170154 0.7476263721 -0.2044985266
C -2.5168276482 2.751995238 1.9794870413
H -2.1845550334 2.2874163899 -0.0786044796
C -1.6613997941 2.605917529 3.2368698197
H -2.7298981281 3.8039583568 1.7654365705
H -3.4786002082 2.2380549673 2.1120019145
C -1.1529673021 1.164771525 3.3562259957
H -0.806337168 3.2895345392 3.1780913442
H -2.2476196524 2.883775185 4.1180284482
N -0.478384516 0.6806500625 2.1434944873

H -0.4455766435 1.0812673648 4.1886244743
H -1.9883788466 0.4901438792 3.5932101398
H -1.1927817924 -1.4452811273 1.1378967053
C 0.5266522586 1.7665423694 -0.9343630416
H 0.6029085659 1.4694962701 -1.9797221809
H -0.0332988799 2.7024843015 -0.9127166122
C 1.8900736073 2.0033773925 -0.3684762187
C 3.1017348897 1.3930735009 -0.5233592558
C 4.0268492261 2.0995525041 0.3131017935
C 3.3128089034 3.0891246946 0.9171159115
O 2.0103637328 3.0477649055 0.5146800627
H 3.567699917 3.8711937997 1.616865425
H 5.0811260291 1.8977347317 0.4420524849
H 3.2999364109 0.5368214077 -1.1514174046
C -0.2723249918 -0.5779467709 -0.5701236258
C -0.8037615181 -1.6102481543 0.1440866917
C 0.3659682 -0.8609104022 -1.9245982015
C -0.8579537544 -2.9635194063 -0.3861350727
O -0.5296774642 -0.7281215534 -2.906575967
O 1.5462102592 -1.0940736174 -2.0781092962
C -0.0465252993 -1.008938569 -4.240887479
O -1.4343502697 -3.8059569078 0.5104195346
O -0.4537096515 -3.3483676338 -1.4789263607
C -1.5419981034 -5.1776225358 0.0963364317
H -0.8974824237 -0.8388072918 -4.899456562
H 0.7760337902 -0.3390872116 -4.5006335143
H 0.2850986997 -2.0472247709 -4.3064614962
H -2.0234284153 -5.6988713142 0.9245227632
H -2.151234992 -5.2673571325 -0.8072997254
H -0.5549662279 -5.6080414367 -0.0936789972

E-**12b_t1**
C -1.2465179676 0.9773138589 0.4205094631
N -1.065576942 2.2918961662 0.7852352617
N -0.2215934505 0.4119929326 -0.3641363047
C -2.1089941119 2.9265196778 1.6021367797
H -0.6306555278 2.8873513638 0.0902229008
C -3.4772337292 2.3360835025 1.2628785355
H -2.0682695536 4.0018630167 1.4163621922
H -1.88558859 2.7659273982 2.6644313506
C -3.3989063975 0.8112963511 1.3896418855
H -3.7472099895 2.616067054 0.2374427232
H -4.245581474 2.7368760187 1.9319658917
N -2.2303043602 0.2073955554 0.7454789817
H -4.297486061 0.3485175806 0.962679806
H -3.3929521481 0.5317539291 2.4536531883
H -0.2226710153 -2.5425348409 -2.0377467978
C 1.1218579378 1.0257535625 -0.4543902217
H 1.8700990456 0.2361275807 -0.4551838092
H 1.2730412691 1.6070392844 0.4579876558

C 1.306216071 1.8852612972 -1.6598550073
C 2.0122536553 1.7437694416 -2.8175687623
C 1.776667139 2.9283376384 -3.5942516311
C 0.9421152769 3.708097972 -2.856469829
O 0.6466985797 3.0920418155 -1.6712446104
H 0.4909464657 4.676517351 -3.0125309243
H 2.1782485858 3.1641031654 -4.5699031552
H 2.6279841958 0.8962469294 -3.0849136881
C -0.5628380401 -0.7063257446 -1.1232841593
C 0.1619830863 -1.8379915435 -1.3108734795
C -1.8476828193 -0.6359716955 -1.9291325298
C 1.3456832282 -2.2856073285 -0.5698995631
O -2.1012237382 0.6110785868 -2.3534731065
O -2.50844049 -1.6078023986 -2.2388093902
C -3.2926161655 0.7675109199 -3.1517100667
O 1.7626262747 -3.4800653357 -1.0502622008
O 1.8923735293 -1.7258924803 0.371165638
C 2.8881912324 -4.0694492623 -0.373379558
H -3.3418407681 1.8277371359 -3.3982909184
H -3.2254533256 0.1683613655 -4.0628471339
H -4.1761501577 0.4684268757 -2.5826051239
H 3.0852477831 -5.0067568803 -0.8938447178
H 3.7613671396 -3.4144921173 -0.430843832
H 2.6510343274 -4.2637627001 0.6759322671

12b_t2
C -0.1368919183 1.8124300546 0.5445950496
N -0.3761077546 2.9396250881 1.2875447046
N 1.0331062416 1.3598443854 0.24333247
C -1.6566214882 3.5536388907 1.6494462071
H 0.4293967233 3.5317126146 1.4308903245
C -2.8108367945 2.6132492162 1.3341016015
H -1.6350425517 3.8159810999 2.7125460641
H -1.7763976418 4.4855434593 1.0816209612
C -2.5220170613 1.9461672973 -0.002636085
H -2.9183892803 1.8409047382 2.1026953612
H -3.7453750926 3.179352708 1.2872976652
N -1.3002927252 1.1155973342 0.1143117406
H -3.3551350378 1.3295300416 -0.3269079238
H -2.3587026859 2.6982016676 -0.7833757711
C 2.196033436 2.0507260466 0.7904029003
H 2.2877940834 3.0793532968 0.4070051235
H 2.1282977273 2.138005921 1.8887604432
C 3.4637978679 1.3460479794 0.4339319163
C 4.558506118 1.702676358 -0.2977188516
C 5.4638609402 0.5879149736 -0.2580252135
C 4.8524026561 -0.3661971221 0.4941959371
O 3.6344073826 0.0782351811 0.9272023015
H 5.1288578269 -1.3637028447 0.8021407094
H 6.4343702041 0.511301408 -0.7284846957

H	4.7020788016	2.6461945943	-0.8061644001
H	0.7195934344	-0.7303289341	-0.1980670981
C	-1.3406523129	-0.2424054651	-0.1531625043
C	-0.2894559313	-1.1023330616	-0.2629384794
C	-2.7541812384	-0.8080692556	-0.2389142776
C	-0.4854816946	-2.5363059854	-0.4469549114
O	0.7122259447	-3.1616246746	-0.5837703404
O	-1.5480669541	-3.1476638967	-0.4792087788
C	0.655097304	-4.585637595	-0.7676709211
O	-3.1456765367	-0.9546799359	-1.507990309
O	-3.4410929407	-1.0215222319	0.7377789483
C	-4.4488136272	-1.5500312425	-1.6963798497
H	1.6918888189	-4.915058856	-0.8439017572
H	0.1691518567	-5.0712689337	0.0830050132
H	0.113338495	-4.840757259	-1.682664967
H	-4.5997739624	-1.5850035524	-2.7748060229
H	-4.4613690984	-2.5580694193	-1.2766379272
H	-5.2235925338	-0.941860089	-1.2239033575

E-**12b_t2**

C	0.212151242	0.4067795676	0.6131563406
N	0.3960848261	1.4925028836	1.4268876498
N	1.1285366406	-0.1993247743	-0.0820603624
C	-0.6795245422	2.2469487124	2.0703430935
H	1.2586804255	1.9975155193	1.2817863879
C	-1.8169970318	1.2998923992	2.4232711204
H	-0.2695069132	2.723349129	2.9656021833
H	-1.0472648675	3.0441689752	1.4083210114
C	-2.2339835048	0.5255081087	1.1813733257
H	-1.4965708477	0.6021484079	3.2041376891
H	-2.6714483203	1.8683359149	2.8015867221
N	-1.065306554	-0.1518942919	0.5848151067
H	-2.9729129805	-0.2341410508	1.4322036382
H	-2.6784604755	1.2052016083	0.4411181898
C	2.5007217572	0.2658742205	-0.0047065104
H	2.8085650737	0.5101602908	1.0276267821
H	3.1561498046	-0.5609865977	-0.3114833503
C	2.8077125979	1.4462016419	-0.8871103229
C	2.0760189733	2.2178377291	-1.7419356981
C	2.9837889632	3.1806330871	-2.3054773112
C	4.1996688359	2.9236900672	-1.7555764802
O	4.1122454936	1.868265312	-0.8873253729
H	5.1780131467	3.3683477291	-1.8623375645
H	2.7543531223	3.9558037486	-3.0236964064
H	1.021413865	2.1124600985	-1.9466968508
H	-2.028021772	-3.0627179167	-0.8722284541
C	-1.2754789487	-1.2030357038	-0.3261764427
C	-1.833336637	-2.3980688441	-0.0386801408
C	-0.9731679877	-0.958577021	-1.7903196928
C	-2.1913652239	-3.0034184264	1.2596302296

O	-1.8870706324	-2.2622770412	2.3413790792
O	-2.6991027662	-4.1120612164	1.3313626594
C	-2.1869601251	-2.8704277573	3.6149805534
O	-1.1772749932	0.3257076499	-2.1281364015
O	-0.6899468056	-1.8332464786	-2.5852627303
C	-0.9854016212	0.6361425171	-3.5243246136
H	-1.8748817395	-2.1450766309	4.3657731658
H	-3.257213699	-3.0716329545	3.7035822519
H	-1.6310517403	-3.8032828716	3.7357218953
H	-1.2056756685	1.6988130586	-3.6216827186
H	0.0446996721	0.4315980981	-3.8246296135
H	-1.669070042	0.0500891026	-4.1432840372

TS4$_{5C}$(1b_t1)

C	-0.7848726758	1.2373986389	0.423895494
N	-0.3912331158	2.3689176981	1.0390995579
N	0.1458001859	0.290284674	-0.0171275185
C	-1.3791048009	3.4279839845	1.3052945173
H	0.5715932381	2.6642714769	0.9465600839
C	-2.7623045893	2.8143657699	1.5182014379
H	-1.0531280785	3.9749663598	2.1932969072
H	-1.3964572937	4.1300597804	0.4625343607
C	-3.1063254529	1.8433993215	0.3828775019
H	-2.7828089184	2.2778605223	2.473030477
H	-3.5067149759	3.6138984968	1.5692645905
N	-2.015819047	0.897017816	0.1897347087
H	-4.01223282	1.2803305942	0.6244965619
H	-3.2938418899	2.3842069578	-0.5537710759
H	-0.7049469727	-2.3871620253	-1.905491762
C	1.5930630946	0.4459014204	0.2448008286
H	2.0244036716	-0.5530692095	0.2672641324
H	1.7074099199	0.886121477	1.238810553
C	2.3168242693	1.2464998655	-0.7837760774
C	3.148195975	0.91274671	-1.8114997458
C	3.5319340514	2.1414317335	-2.4460904967
C	2.9059948392	3.1363303198	-1.7615125068
O	2.1615387763	2.61197083	-0.7415225839
H	2.8851213393	4.2114909112	-1.8587444301
H	4.1869280252	2.2635746484	-3.2973843984
H	3.4500829087	-0.0901841725	-2.0772115362
C	-0.445923043	-0.713300275	-0.7559597026
C	0.0401562475	-1.845530949	-1.3334290456
C	-1.950895114	-0.4021054363	-1.0369534854
C	1.3451347139	-2.4977775644	-1.3397252216
O	-2.2682008632	0.0498551852	-2.1535174943
O	-2.7075864641	-1.4329433877	-0.448363116
O	1.3304555872	-3.5155560769	-2.2397244064
O	2.3429004266	-2.2665597752	-0.6604308608
C	2.5378426089	-4.2894994168	-2.3318889595
H	2.3341586655	-5.0636489404	-3.0723921506

H	3.3760147256	-3.6687349929	-2.6600636433
H	2.7849612044	-4.7456576589	-1.3695602383
C	-4.0244049101	-1.5776800912	-0.9780424394
H	-4.4443320221	-2.4660628631	-0.4996275925
H	-4.6612894421	-0.715309702	-0.745378313
H	-4.0109749847	-1.7160726549	-2.0632939122

TS4$_{5C}$(1b_t2)

C	0.2375888805	0.6836892289	0.602319961
N	0.6402785687	1.6244387006	1.4767676606
N	0.8521370176	0.3024103329	-0.4741810795
C	-0.1221805755	2.0790114579	2.6456145691
H	1.391229827	2.2243693367	1.1575316045
C	-1.1182434295	1.007545836	3.068548603
H	0.5868323611	2.3014324854	3.4477626323
H	-0.64867162	3.0084846551	2.3945202156
C	-1.8913369937	0.4966904798	1.8572991118
H	-0.5984086588	0.1692886056	3.5452142306
H	-1.8158677353	1.429057487	3.7974093056
N	-0.9398438149	-0.0288764407	0.8593912184
H	-2.5621248084	-0.3092763578	2.1399424367
H	-2.4720311179	1.3048173834	1.3947360736
C	2.2497498373	0.5255055798	-0.7891318177
H	2.861167355	0.4566775249	0.1231511997
H	2.551390047	-0.2993425172	-1.4442983755
C	2.5462702862	1.8135174504	-1.4880461761
C	2.9977857259	2.1385257414	-2.7326720743
C	3.0962851619	3.5716230811	-2.7765723874
C	2.69745769	4.0192174363	-1.5568765684
O	2.3616734281	2.9616023927	-0.7540578124
H	2.5977803257	4.999351889	-1.1152006533
H	3.420454772	4.1815929645	-3.6082088534
H	3.2382198948	1.4403613784	-3.522308287
H	-1.9847861239	-2.7552630753	-0.8583111752
C	-1.1509965816	-1.0769866274	-0.0146063559
C	-2.018474881	-2.1229571135	0.0221203533
C	-0.2276303507	-1.0209923849	-1.2603513163
C	-2.9086785991	-2.6171280282	1.0680792956
O	-3.672634983	-3.6267866113	0.5754500995
O	-2.9843502653	-2.2686521382	2.2420951689
C	-4.5562376295	-4.2591135262	1.5162968132
O	-0.9313986285	-0.2640678679	-2.2076919697
O	0.4785953051	-1.9778496138	-1.6001128106
C	-0.3444108588	-0.2319914607	-3.507629532
H	-5.0789699913	-5.0333907396	0.9538399038
H	-3.9942498396	-4.7083836296	2.3396588356
H	-5.273555948	-3.5408179679	1.9221723264
H	-1.0879174571	0.2196609052	-4.1686665042
H	0.5633491138	0.3846454409	-3.5194717404
H	-0.0974397064	-1.2374846737	-3.8596831294

TS4$_{6C}$(1b_t1)

C	-1.3336596986	0.8039537652	0.4285765502
N	-0.8631186225	1.7834841357	1.2420840808
N	-0.4162632431	-0.1042726305	-0.0684887119
C	-1.793630094	2.743655358	1.8495761411
C	-3.1335948577	2.057761004	2.1109108668
H	-1.3319952896	3.1075869288	2.7691056024
H	-1.9292845384	3.6001663925	1.1785853469
C	-3.6416026337	1.4626696103	0.8024169077
H	-3.0011559136	1.274489439	2.8658020437
H	-3.8635253775	2.7760195818	2.495398359
N	-2.6015649236	0.7030616166	0.1033214672
H	-4.480218555	0.7776408081	0.9491251613
H	-3.995110495	2.2625602182	0.1408619722
C	0.984684196	0.3350056739	-0.31509294
H	1.3418870542	-0.1709842535	-1.2129085021
H	0.9456416895	1.4045338153	-0.5473485951
C	1.981876757	0.0951691958	0.7724612875
C	3.2163945614	-0.4792689175	0.7877672623
C	3.747419788	-0.2871819841	2.108009048
C	2.8011255101	0.3915000147	2.8080151705
O	1.7183841176	0.642263595	2.0047859305
H	2.7288408156	0.7500078652	3.8235897573
H	4.7049994519	-0.6197421765	2.4837863291
H	3.6881066349	-0.9895527018	-0.0399021818
C	-0.9067144803	-1.2732068633	-0.7076011785
C	-0.0335755551	-2.4827957994	-0.627165268
C	-3.1414688747	-0.2054217461	-1.2039806605
C	-2.1501353946	-1.3479424404	-1.2081917664
O	-4.3634931372	-0.4770857885	-1.0650772263
H	-2.5136588596	-2.2894172245	-1.602196451
O	-0.3390701003	-3.381871944	-1.5801106918
O	0.8369134757	-2.6526127329	0.2063160744
C	0.3812918194	-4.6298667651	-1.5151311663
H	0.0035770304	-5.2272830067	-2.344565285
H	1.4544100307	-4.4593322221	-1.6287978607
H	0.1912241987	-5.1367802637	-0.5658690286
O	-2.7379234379	0.6158684307	-2.3381545997
C	-3.7380412205	1.4995987332	-2.8213385926
H	-3.4695108838	1.7589892963	-3.8506835319
H	-4.7249272956	1.0260209314	-2.8067267265
H	-3.789148215	2.4293966724	-2.2344781965
H	-0.0217174345	1.5709673784	1.7655728022

TS4$_{6C}$(1b_t2)

C	-0.7801629908	0.9263013722	0.2111980959
N	-0.5258644377	1.9606411357	1.0467675229
N	0.2074765446	0.1934701273	-0.2977098809
C	-1.5696810737	2.7625666651	1.6890583435

C	-2.7825019579	1.8762047711	1.9258100676
H	-1.1560504162	3.1482937226	2.6223204878
H	-1.8343440428	3.6175363639	1.0549934821
C	-3.21905131	1.2813890954	0.5966624353
H	-2.5303177965	1.0885616719	2.6438538577
H	-3.6122669562	2.4585715504	2.3355905032
N	-2.0778766382	0.6633477788	-0.135543872
H	-3.9672826488	0.5013117302	0.75338576
H	-3.6615443724	2.0522444444	-0.0361880358
C	1.6020403931	0.5365347395	-0.0188871275
H	2.1822967459	-0.0217846095	-0.757087697
H	1.7769215873	1.6006959142	-0.2142596601
C	2.0742515276	0.2183728607	1.3741564614
C	1.9522396473	-0.8628995683	2.1965680236
C	2.6793653171	-0.5490796147	3.393532171
C	3.1918516029	0.6989675552	3.2153207321
O	2.830654445	1.187682405	1.9899399315
H	3.7976091182	1.3529202699	3.8249660826
H	2.8019785157	-1.171650921	4.2692388555
H	1.3959284858	-1.755254292	1.9466127527
C	-0.0729017109	-1.3515740064	-0.8509058179
O	0.0285421642	-2.2298845782	0.0525860377
C	-2.3480550148	-0.3147511201	-1.1252419135
C	-1.4266961759	-1.2159961388	-1.5022495424
C	-3.634350949	-0.1880086242	-1.8719761836
O	-4.0327058741	-1.3686747849	-2.3787243676
O	-4.2407384581	0.8555113279	-2.0436753096
C	-5.1972040533	-1.3220763588	-3.229296712
H	-5.3620624497	-2.3495941012	-3.5524455804
H	-6.0634156939	-0.9558867731	-2.6734038151
H	-5.0187375456	-0.6772453304	-4.0932650495
H	-1.6629117979	-1.9204148538	-2.2904092797
H	0.3910277322	1.9917835932	1.4668290874
O	0.9013273349	-1.3844393531	-1.939142335
C	1.6205553355	-2.6067662891	-2.0381723774
H	0.9710022121	-3.4516895879	-2.30740394
H	2.3540216097	-2.4630522182	-2.8374586606
H	2.1430050449	-2.862037971	-1.1110885336

13b_t1

C	-0.8442405877	1.067272046	0.9018406646
N	-0.5490521434	2.1796778543	1.5829139965
N	0.1084319045	0.1146287825	0.5562786547
C	-1.6265211298	3.1220669938	1.9201594589
H	0.409207771	2.4989131924	1.6381912886
C	-2.9190507317	2.3435768181	2.1724017171
H	-1.3166111207	3.6708100564	2.8117052757
H	-1.7605335495	3.8421559572	1.1035226609
C	-3.2463437771	1.4408361854	0.9779269698
H	-2.8037796876	1.7355010115	3.0760126322

H	-3.7392789171	3.0458556862	2.3421549097
N	-2.0532662856	0.7243090621	0.5469150011
H	-3.9881933213	0.6812421119	1.2426531504
H	-3.6418536048	2.02906979	0.1419569122
H	-0.8129146071	-2.7914975966	-0.9164299149
C	1.5233897583	0.2546190965	0.9697487365
H	1.9616034131	-0.7385384026	0.9179834829
H	1.5405521585	0.5918099847	2.0106471853
C	2.3274993703	1.167038075	0.1072697905
C	3.1621275887	0.9635611256	-0.9509439684
C	3.6345079829	2.2560788295	-1.3572388345
C	3.054256192	3.1569804072	-0.5193237217
O	2.2549262561	2.5131082029	0.3844359528
H	3.1034700531	4.2316120082	-0.4266838006
H	4.3179199462	2.4807659922	-2.1641803923
H	3.4061140215	0.0026793896	-1.3802869531
C	-0.4956321907	-0.9775792562	-0.0390897363
C	-0.0254699987	-2.1512590848	-0.534235422
C	-2.0561046182	-0.7643793763	-0.064694072
C	1.3111411924	-2.7095060127	-0.6940862514
O	-2.3568210741	-0.5673725294	-1.4708541317
O	-2.7753622189	-1.564919786	0.593025096
C	-3.7221211194	-0.7600817615	-1.8127251442
O	1.2243215901	-3.9013669736	-1.3410251754
O	2.3984602089	-2.256134388	-0.343263688
C	2.4689668145	-4.5808595404	-1.5733124478
H	-3.7612416882	-0.880986769	-2.899832119
H	-4.1326344149	-1.6522165827	-1.3299012799
H	-4.3483956381	0.1026339789	-1.539572569
H	2.2063144909	-5.5083158203	-2.083331275
H	3.1307196782	-3.980827757	-2.2038034478
H	2.9760230334	-4.8016770009	-0.6301661912

13b_t2

C	0.4920823315	0.7441398694	0.7633162401
N	0.8849801566	1.7118931578	1.5951419696
N	1.0875203182	0.3884718174	-0.3518609176
C	0.1841953594	2.0815587786	2.8326129614
H	1.6014524856	2.3410062394	1.2516869454
C	-0.6738749196	0.9165357813	3.3101582364
H	0.9379542752	2.3519339786	3.5762037485
H	-0.4371896301	2.96457041	2.6418518295
C	-1.5104388838	0.3609886221	2.1611171237
H	-0.0398069168	0.1219225371	3.7181467752
H	-1.3370210459	1.2604129934	4.1083189374
N	-0.6157957494	-0.0357205573	1.0562075954
H	-2.0726065786	-0.5199883312	2.4612152293
H	-2.2118190672	1.1170548764	1.7873089134
C	2.4909545715	0.6197782787	-0.6678777458
H	3.0917435614	0.5585911628	0.2499079821

H	2.779777124	-0.2206918201	-1.307110197
C	2.7751162528	1.9060358022	-1.3686129139
C	3.2512368156	2.2344705122	-2.6026436432
C	3.335807182	3.6683276111	-2.6458754657
C	2.9052031499	4.1133839231	-1.4361818824
O	2.563043173	3.0531385696	-0.6395392246
H	2.7844126163	5.0929030657	-0.9985515882
H	3.6718161862	4.2805571012	-3.4710528576
H	3.5164242634	1.5381803156	-3.3858356194
H	-1.4522145558	-2.7253230454	-0.821335766
C	-0.7635059645	-1.062088996	0.1434257316
C	-1.6010769558	-2.1265375383	0.0707237928
C	0.2817120611	-0.8942993822	-1.0101047109
C	-2.6221693619	-2.6505826885	0.9715642391
O	-3.2839588981	-3.6674692683	0.3582869847
O	-2.8913137742	-2.3171997751	2.1214175829
C	-4.2958918941	-4.3187414708	1.1433853964
O	-0.5093884758	-0.3047547096	-2.0688301896
O	1.0577305734	-1.841860163	-1.2962043727
C	0.130784573	-0.2776406943	-3.336527074
H	-4.7055070536	-5.1014689373	0.5040398867
H	-3.8667556255	-4.7594246738	2.0474027638
H	-5.0838349851	-3.615803933	1.4271069353
H	-0.6474098714	-0.0802049823	-4.0801032405
H	0.882964809	0.522258487	-3.4013826205
H	0.6151213681	-1.233640924	-3.5597067711

14b_t1

C	-1.2435133602	0.8550428915	0.516987055
N	-0.7341035826	1.8292671506	1.3047696922
N	-0.364642454	-0.0656409531	-0.0167605524
C	-1.5924513067	2.846848187	1.9227411596
C	-2.9576494023	2.2382896391	2.2239768469
H	-1.0871640429	3.1911164301	2.8268256523
H	-1.696304758	3.7035999205	1.2463387067
C	-3.516565544	1.6375945176	0.9426003741
H	-2.8539786826	1.4693625726	2.9976906316
H	-3.6436312486	3.0033119987	2.5985121569
N	-2.5378319812	0.7838768524	0.2580489679
H	-4.3914528903	1.0093210455	1.1203559198
H	-3.8187737575	2.4307242721	0.2485914778
C	1.0730338187	0.3090882483	-0.1839575147
H	1.4531031567	-0.2539156045	-1.033083584
H	1.1062006055	1.3680033995	-0.4572516573
C	1.9836690332	0.0757674555	0.9805803389
C	2.9214296527	-0.8643619333	1.2879810168
C	3.5291424643	-0.4632413044	2.5240231707
C	2.9223710929	0.6957681447	2.8923015045
O	1.9858384185	1.0486515424	1.9596258002
H	3.029083181	1.3654338018	3.7323537828

H 4.314131001 -0.9742481201 3.0637680542
H 3.1413555882 -1.7452461341 0.7051860526
C -0.8974118506 -1.1534083128 -0.7645864464
C -0.0310721833 -2.3578973746 -0.9211264225
C -3.1785074371 -0.0693885798 -0.9355928388
C -2.1711193429 -1.1655882859 -1.1904863502
O -4.3611258857 -0.4443383699 -0.6830584832
H -2.5491987814 -2.0421767684 -1.7011604159
O -0.4647136921 -3.1604442438 -1.9089813374
O 0.9452809721 -2.6091519854 -0.2365808905
C 0.2588493941 -4.3976176065 -2.0724222018
H -0.2247004311 -4.908213689 -2.9047844418
H 1.3076775927 -4.200505802 -2.3057726194
H 0.1942817114 -5.0050970478 -1.1664638775
O -3.0123120665 0.9311412887 -2.0317259677
C -3.959648096 0.7869260971 -3.0746036394
H -3.8962230387 1.6878357143 -3.6948653881
H -3.7531540663 -0.0837866302 -3.7187677553
H -4.9782195643 0.6883248082 -2.6842693512
H 0.1626887638 1.6667217674 1.7453033739

14b_t2

C -0.6788861167 0.9367305257 0.1698068309
N -0.3939898309 1.9970884213 0.9584466751
N 0.2929168124 0.1438213546 -0.2822332612
C -1.40063772 2.8792202839 1.5501499644
C -2.6596386177 2.0674758677 1.8028017133
H -0.9799358909 3.2788447053 2.4747849881
H -1.611570502 3.7213882244 0.8794744655
C -3.1001102885 1.4280622298 0.4962500173
H -2.4626451041 1.3045944881 2.563529585
H -3.4661681221 2.7106024083 2.164986009
N -1.9831234392 0.7024365473 -0.1720273772
H -3.8961446656 0.7041056107 0.6784596393
H -3.4841579428 2.1861830769 -0.1886293953
C 1.6962111668 0.3856839638 0.0831576216
H 2.2660459899 -0.2815290804 -0.563288063
H 1.9821935866 1.4094572593 -0.1820080001
C 2.0466490863 0.1353897423 1.5199087728
C 2.1065597287 -0.9781861527 2.3031331183
C 2.5085346998 -0.5423838105 3.609890248
C 2.6606137708 0.8076435953 3.5349482409
O 2.3831623365 1.2430129955 2.2676739189
H 2.9511714801 1.5687782153 4.2436140396
H 2.6645643453 -1.1553730862 4.4869045891
H 1.8632994992 -1.9757113461 1.9685464059
C 0.0348606572 -1.3240502276 -0.887515199
O 0.3128768462 -2.2608642282 -0.0803317612
C -2.288003135 -0.3582918519 -1.062537197
C -1.3758683746 -1.2787753197 -1.4124175131

C	-3.6090490238	-0.3084996483	-1.7556756106
O	-4.0304554735	-1.5361958877	-2.1082486475
O	-4.2203974724	0.7131268384	-2.0175668341
C	-5.2300581203	-1.5787181123	-2.9089744349
H	-5.4087074587	-2.6350558431	-3.1082309413
H	-6.0719412656	-1.1487131958	-2.3614242349
H	-5.088170382	-1.0346406505	-3.8459591764
H	-1.6516108028	-2.0567596942	-2.1133285374
H	0.5466552247	2.0818288509	1.3130192077
O	0.9001236827	-1.2070691003	-2.090836479
C	1.3936874587	-2.4540014345	-2.5509443046
H	2.152416039	-2.2349476368	-3.3094953213
H	1.8453910254	-3.0371339076	-1.7421795643
H	0.6087073128	-3.0684249904	-3.0207821976

15b_t1

C	-0.583517636	1.4158290082	0.0606155673
N	-0.1730851595	2.5843815011	0.3738494269
N	0.2779537939	0.3751584111	-0.3496501893
C	-1.2153855007	3.5602663851	0.7261441184
C	-2.5492641901	2.9430729623	1.1747344174
H	-0.8233437869	4.2054831336	1.5204717589
H	-1.3819585263	4.2125580066	-0.1430932663
C	-2.9893722371	1.8373522335	0.2109975371
H	-2.4394204588	2.5167959688	2.1784397219
H	-3.3217787496	3.7163456617	1.2260806183
N	-1.8647519899	0.9157693123	0.0690032915
H	-3.8461400292	1.2875153669	0.6116602133
H	-3.2761981189	2.253789196	-0.7624578011
H	-0.9099867061	-2.7425190837	-0.9717207528
C	1.7329353718	0.5340141885	-0.1938432054
H	2.2122967297	-0.1171105051	-0.9239160532
H	1.9591141364	1.5760681968	-0.4207626056
C	2.22202683	0.2332624093	1.1843923125
C	2.6738550629	1.0181084628	2.2054557615
C	3.0060844104	0.1359998679	3.2872845022
C	2.7260360228	-1.1214317285	2.8483235527
O	2.2502654241	-1.0839876882	1.5688542042
H	2.8062160105	-2.1017335243	3.2944783099
H	3.3987266887	0.4050332188	4.2580131043
H	2.7555788017	2.0959103013	2.1867583198
C	-0.4318434919	-0.7637489344	-0.63670417
C	-0.0837838035	-2.0434033718	-0.9378479567
C	-1.9245753788	-0.3664528638	-0.5808918668
C	1.2205253351	-2.6342921136	-1.2231502605
O	-2.4864677736	-0.1308416137	-1.8605177515
O	-2.6434431329	-1.3338938062	0.1198239464
C	-2.6920044793	-1.2530316523	-2.7270393219
O	1.1386027087	-3.9866773608	-1.1264099877
O	2.2576881948	-2.0745153408	-1.5595763355

C 2.3371326637 -4.7102864182 -1.4520768509
H -3.288185476 -0.8750261792 -3.5603439578
H -1.7448207613 -1.6441675676 -3.1126792593
H -3.2416057307 -2.0581183068 -2.2296295975
H 2.0854171646 -5.764633398 -1.3330779809
H 2.6459336637 -4.5119846989 -2.4820853723
H 3.1522954825 -4.4406513702 -0.7753635334
H -3.5889313786 -1.1270062664 0.019504392

E-**15b_t1**
C -0.8956222044 1.6364364144 0.2315103859
N -0.6285635901 2.7897441417 0.7138861842
N 0.0917778353 0.7445106719 -0.2408491551
C -1.7920476423 3.5834328009 1.1354778772
C -3.0234890743 2.7430306177 1.5042301872
H -1.4932109848 4.1971633981 1.9925413105
H -2.049303128 4.2856467818 0.3291675839
C -3.3403948467 1.7277977201 0.4017518486
H -2.8335488637 2.2071072102 2.4413100721
H -3.8897914509 3.3922834368 1.6633975741
N -2.106833023 1.0126464584 0.0846179417
H -4.0865004676 1.0022983434 0.7356549255
H -3.7310091084 2.2364413031 -0.4891846746
C 1.5208661873 1.0564937303 -0.1747279122
H 1.9696980621 0.8784467986 -1.1560041439
H 1.5748880277 2.1266766224 0.0407185537
C 2.2822774536 0.2713885614 0.8433847336
C 3.3488949061 -0.574665963 0.7529250083
C 3.6707927982 -0.9725466786 2.0938032018
C 2.7741601917 -0.3421184632 2.8997001608
O 1.9227089446 0.424997725 2.1562935961
H 2.6098769879 -0.3317813271 3.9669246529
H 4.460189402 -1.6403208327 2.4092807029
H 3.8456409758 -0.8763404438 -0.15888895
C -0.4653664856 -0.3989678569 -0.7333918327
C 0.2176809078 -1.4673256918 -1.2311576314
C -2.0014068305 -0.2198947302 -0.6660719019
C -0.4010174528 -2.6913187634 -1.701813673
O -2.4159129621 -0.077902934 -2.0181270132
O -2.6722963789 -1.2267727615 -0.001476657
C -3.7899529834 -0.3503788686 -2.3144297239
O 0.4981854746 -3.4799905326 -2.3241370686
O -1.5787251134 -3.0504110671 -1.5669267967
C 0.0133193094 -4.7505285249 -2.7981849086
H -3.9134889621 -0.095582056 -3.3696838877
H -4.0355858166 -1.407235067 -2.1733493279
H -4.4809136504 0.2588617717 -1.7227132432
H 0.8763082937 -5.2413865919 -3.2482848953
H -0.3740896034 -5.3513368249 -1.9716128822
H -0.7726378601 -4.6117877034 -3.5446665452

H 1.29848022 -1.4335662666 -1.2796060235
H -2.437631494 -2.0496955582 -0.5025056531

15b_t2
C 0.4156191243 0.6411218109 0.8212510094
N 0.8922562074 1.6171735584 1.4925749084
N 1.028618435 0.0629125827 -0.267819319
C 0.1190043956 2.0944574626 2.6434978784
C -0.8811166045 1.0738688749 3.1935849094
H 0.8250391258 2.3891184374 3.4284066816
H -0.4105348448 3.0121805309 2.3487352188
C -1.7111849404 0.4651633649 2.064330746
H -0.345845229 0.2723116035 3.7164787298
H -1.5513655576 1.549435974 3.9165130738
N -0.7924970089 -0.0541258825 1.0402470032
H -2.3376038789 -0.3539547097 2.4093048878
H -2.3535978984 1.225637493 1.6012706154
C 2.3029268767 0.5112213662 -0.7950659046
H 3.0099731374 0.6074085125 0.0348412085
H 2.6893930102 -0.2852696742 -1.4409690681
C 2.2712815629 1.8229788828 -1.5306284063
C 1.3878647267 2.4516620835 -2.3583811355
C 2.008834876 3.6835754572 -2.7576198202
C 3.2257240058 3.7117002762 -2.1506988734
O 3.4019813235 2.5854071925 -1.3958594354
H 4.0421186957 4.418441859 -2.1438922821
H 1.5983428055 4.4431821007 -3.4086680419
H 0.4152396283 2.0779354113 -2.6407030853
H -1.6811745965 -2.7348914672 -0.8444008273
C -0.9545502053 -1.0710188022 0.1392208879
C -1.8405641135 -2.0969190983 0.0148607722
C 0.1678283057 -0.8995466958 -0.910560347
C -2.9295764874 -2.5328588538 0.8822023347
O -3.6287945704 -3.5211368566 0.2636247886
O -3.2182748534 -2.1536328574 2.0118369685
C -4.7064406653 -4.0936379982 1.022790108
O -0.2643557173 -0.2980959183 -2.1201302758
O 0.7649379858 -2.1356650391 -1.1522326338
C -1.1398844064 -1.0391869367 -2.9809883069
H -5.1384332705 -4.8647365333 0.3841053593
H -4.3377700985 -4.5386680716 1.9510173742
H -5.4605146552 -3.3389960015 1.2620696667
H -1.1580550307 -0.4891731921 -3.9240593718
H -0.7708592708 -2.0526734773 -3.1653059697
H -2.1555805073 -1.0882292728 -2.5754601326
H 1.3334191826 -2.0420934961 -1.9360918939

E-**15b_t2**
C 0.4042799997 0.6701104158 0.7344845358
N 0.8624043366 1.6230679668 1.4511528051

N 1.0406242001 0.0977739665 -0.3412168638
C 0.0215220055 2.0577619279 2.5726904903
C -0.8608609382 0.9422842919 3.1412824478
H 0.6774366475 2.4473993678 3.3584457332
H -0.6051448439 2.9009092386 2.2462953825
C -1.6880972474 0.2783254057 2.0389919603
H -0.2263472392 0.1875267305 3.6200083155
H -1.5380539831 1.3376564699 3.9041946133
N -0.8158019988 -0.0174054993 0.8985503695
H -2.1261360227 -0.657814374 2.3939131699
H -2.5028870638 0.9314457201 1.7051305526
C 2.3270447602 0.5352484636 -0.8503465295
H 3.0407131169 0.5757360336 -0.0211386267
H 2.6730751933 -0.2479823926 -1.5319396848
C 2.3310303285 1.8776471649 -1.5295742168
C 1.4790812538 2.550242404 -2.3559395564
C 2.1255420734 3.7887386277 -2.6883877367
C 3.3256276969 3.7766556392 -2.0481434779
O 3.4668735951 2.6193202762 -1.3340008284
H 4.1503425247 4.4714279723 -1.9913384761
H 1.7429477184 4.5785581355 -3.3202250248
H 0.5104729929 2.1992749162 -2.678476287
C -0.9680483904 -1.0135115175 -0.0195468726
C -1.9911654608 -1.9194253508 -0.0186284839
C 0.1901248842 -0.8371991506 -1.0359341574
C -2.0604392083 -3.1114620338 -0.8368123691
O -3.2295930168 -3.7553554645 -0.6664245318
O -1.1862025505 -3.5652500188 -1.5919149705
C -3.3899969309 -4.9939135026 -1.3856193568
O -0.2839222167 -0.1375295574 -2.1901055884
O 0.8777305711 -1.9846994518 -1.3521779859
C -1.0068770075 -0.8615976103 -3.1862160784
H -4.3775687881 -5.3633118196 -1.1095959391
H -3.3382063582 -4.8250063034 -2.4638848529
H -2.6218926763 -5.7139288939 -1.0930354953
H -1.113284038 -0.1755091415 -4.0296276194
H -0.4651241553 -1.7518932182 -3.5174554883
H -2.0066792226 -1.1477065302 -2.8394579616
H -2.7929011988 -1.8016733522 0.7002852075
H 0.2011176577 -2.698089952 -1.5063795232

16b_t1

C -1.2435395881 1.0101853507 0.5047423524
N -0.6696315022 1.9795454766 1.1318394542
N -0.4108799049 0.0281965196 -0.070163033
C -1.5113593433 3.0184662637 1.7170274975
C -2.8529471095 2.4661263022 2.1916686137
H -0.9648098675 3.471305462 2.5522110927
H -1.6799584392 3.8241529286 0.9856154513
C -3.5333295643 1.7494866496 1.0337477053

H -2.6888051348 1.7671275711 3.0202412635
H -3.5091835339 3.2633646668 2.5552053647
N -2.6122360375 0.7885819899 0.3944282846
H -4.4023490615 1.1906666407 1.390330846
H -3.8788574097 2.4790000691 0.2906016446
C 1.0256696106 0.3394499873 -0.1838345767
H 1.4220494535 -0.1728971377 -1.0611563677
H 1.0970625038 1.4146382455 -0.3663718627
C 1.8729429281 -0.0342142128 0.9977554149
C 1.7650948686 0.084911321 2.3532963929
C 2.9780991447 -0.4458833738 2.9091022019
C 3.7411748765 -0.8366957848 1.8529214862
O 3.0826930664 -0.5933113536 0.6811011586
H 4.7217579071 -1.279777318 1.7621257917
H 3.2418886254 -0.5202966302 3.9552346067
H 0.9273157177 0.5062433142 2.8876256577
C -0.9673268138 -1.0789596048 -0.7071638544
C -0.1065925622 -2.2923558109 -0.905137349
C -3.1559517672 0.0413855945 -0.7360184277
C -2.2749038321 -1.1353076884 -1.0138434041
O -4.4370181893 -0.4531834147 -0.4473422973
H -2.7157373571 -2.0243450674 -1.4402862567
O -0.5251432045 -3.0244903322 -1.9488123445
O 0.8316065824 -2.5954894357 -0.1940088667
C 0.1717985629 -4.272825288 -2.155868967
H -0.2964315485 -4.7244810952 -3.0297637434
H 1.2321656702 -4.0902408955 -2.3437801826
H 0.060487448 -4.9234444175 -1.2853143414
O -3.2272049327 0.9710266524 -1.8176121975
C -3.6447594155 0.4354034437 -3.0777771642
H -3.7540809677 1.2897057876 -3.7493470273
H -2.8970223624 -0.2502483634 -3.4935741737
H -4.6059304456 -0.0848558393 -3.0017614259
H -5.0618220712 0.285903827 -0.5360884176

16b_t2
C -1.1971393144 0.9200951055 0.4952855566
N -0.6383057615 1.89844668 1.1182753203
N -0.3483683509 -0.0083016431 -0.1443302076
C -1.4950455705 2.8704522557 1.7898151944
C -2.7837817205 2.2369663473 2.3079385032
H -0.9265549838 3.3151033894 2.6149631475
H -1.7373454304 3.6958937615 1.102571571
C -3.4888625366 1.5330080142 1.1571193111
H -2.545395959 1.5157905226 3.0986144906
H -3.4560973488 2.9876492467 2.7355978
N -2.5599836572 0.6358892699 0.4405664502
H -4.3160711471 0.9244417917 1.531808978
H -3.8995931992 2.2731892001 0.4591419723
C 1.0768343333 0.345848585 -0.2387842115

H 1.5109870204 -0.2429850352 -1.0482683432
H 1.1520110328 1.3988810257 -0.5187960916
C 1.8539079716 0.1518996835 1.0300531954
C 1.8896939986 -0.8001437595 2.0062538766
C 2.9037087716 -0.3928951029 2.9377180166
C 3.4191670736 0.7699882486 2.4551111863
O 2.7887553687 1.1178528866 1.2931673473
H 4.1941273716 1.4419321125 2.792594984
H 3.2064822617 -0.9033202583 3.8417993268
H 1.2762742585 -1.687333081 2.0460182074
C -0.8866018401 -1.0889162053 -0.8389322899
C 0.0029662467 -2.2572487809 -1.1521434425
C -3.1208794604 -0.064686051 -0.7102147033
C -2.2023227178 -1.1777942137 -1.1021739101
O -4.3647622999 -0.635512449 -0.3982737977
H -2.6225788601 -2.0529020537 -1.5753983492
O -0.4202613906 -2.9169490997 -2.2394672866
O 0.9667217278 -2.5900308483 -0.4881446326
C 0.302703095 -4.126826493 -2.5592092201
H -0.1744696033 -4.5202428614 -3.4559688346
H 1.3534664279 -3.9016690582 -2.7543190531
H 0.2269728831 -4.8461943967 -1.7405096375
O -3.2843554332 0.9251706488 -1.7276062281
C -3.7346755839 0.4477536213 -2.9996220081
H -3.9180867862 1.3350747084 -3.6094249793
H -2.9746880303 -0.1708110255 -3.4916011393
H -4.6642913546 -0.1257324974 -2.9143830916
H -5.0251295024 0.0772088084 -0.4192649781

17b_t1
C -0.9021126239 1.5224096252 0.2436815279
N -0.5829690675 2.6237958181 0.7911948885
N -0.0016620582 0.5283096133 -0.1713734586
C -1.689760824 3.5251349599 1.1445078482
C -3.0265197464 2.8115504232 1.3897062673
H -1.3944388371 4.0809726251 2.0398026831
H -1.7972564556 4.2650431285 0.3391803144
C -3.3707869001 1.8747201003 0.2309708092
H -2.9649215596 2.2312933632 2.3171844636
H -3.8271852201 3.5466456809 1.5099782812
N -2.187411914 1.0595155369 -0.0549269364
H -4.1933048836 1.1996457174 0.4753101223
H -3.640110885 2.4374496321 -0.6695461703
H -1.1461785388 -2.3702015751 -1.5546129322
C 1.4251824584 0.6164908802 0.1883578939
H 2.012311774 0.2715806012 -0.6596553692
H 1.6184674533 1.6763986352 0.3645953463
C 1.7934197074 -0.1926801691 1.3873324817
C 2.5444912724 -1.3202733229 1.5526782757
C 2.5586950442 -1.6018934268 2.959353175

C	1.8111985807	-0.6280233669	3.5465252511
O	1.339213829	0.242160522	2.606761023
H	1.5322680501	-0.4171533744	4.5682503304
H	3.0571393921	-2.419728957	3.4608090779
H	3.0260829035	-1.8780030682	0.7628277047
C	-0.6850168159	-0.5351568776	-0.7230929918
C	-0.3226873534	-1.7154386351	-1.2881330706
C	-2.149840894	-0.1760892575	-0.6254102193
C	1.0060878407	-2.2193545985	-1.6386721946
O	-3.2394928328	-3.3489554771	-2.2714709262
O	-3.1236588142	-0.8275236605	-0.9926202193
C	-3.3612169028	-2.9963868932	-3.6424917575
O	0.9319702812	-3.5406163941	-1.9187375416
O	2.0512991573	-1.5869684833	-1.7340699602
C	2.1550570368	-4.1627966852	-2.3537917644
H	-3.2866990332	-3.9157840735	-4.2321336786
H	-4.3296368103	-2.5261759684	-3.8710213973
H	-2.5647882762	-2.3152028914	-3.9770143359
H	1.8957079769	-5.1999848898	-2.5667296108
H	2.5386389722	-3.6766030286	-3.2544049479
H	2.9141465705	-4.1191223842	-1.5685288222
H	-3.2709200538	-2.517082404	-1.7648154602

E-**17b_t1**

C	-0.9893296058	1.5113328647	0.2982494621
N	-0.7269325843	2.6000842177	0.9032401351
N	-0.058497692	0.5307041721	-0.0541280482
C	-1.8706031977	3.487914716	1.161131652
C	-3.2182905786	2.7589625836	1.2595287887
H	-1.6710729891	4.0288730185	2.0915046488
H	-1.9070916078	4.2430141488	0.3632775324
C	-3.4412863503	1.8481406856	0.0507695301
H	-3.2396625118	2.156397151	2.1746428573
H	-4.0340343829	3.4848073882	1.3208691589
N	-2.2274901459	1.053951206	-0.1449385274
H	-4.2752930791	1.1593932766	0.2008579609
H	-3.6367156246	2.4324129894	-0.8554807516
C	1.372325203	0.6827197276	0.2222938333
H	1.9363103002	0.5049749969	-0.6974688071
H	1.5057513061	1.7296851837	0.5061724273
C	1.8951174204	-0.2243571663	1.2881175652
C	2.8763838872	-1.1722330197	1.3014036675
C	2.9545365953	-1.6610323889	2.6485453208
C	2.012811554	-0.9772711385	3.3532337284
O	1.359905616	-0.0930184852	2.5421052728
H	1.6924152021	-0.9975870188	4.3844019518
H	3.6195195092	-2.4215020117	3.0333604151
H	3.4723937216	-1.4826231409	0.4543113933
C	-0.6638180784	-0.5203124168	-0.7200868534
C	0.0099173949	-1.5992998522	-1.1980632295

C -2.1373201064 -0.1595396758 -0.7832106262
C -0.5160640781 -2.7638069559 -1.9131105265
O -4.5392661553 -2.910363475 -2.6413217695
O -3.0782358035 -0.7563393098 -1.2782225062
C -4.4081398043 -2.6231231104 -4.019454958
O 0.5266174415 -3.5612589996 -2.2658838169
O -1.6711583167 -3.0543040154 -2.1818133339
C 0.1807926915 -4.7645522424 -2.9725544277
H -5.3132471327 -2.9767875054 -4.5262628956
H -4.3114632055 -1.5446845727 -4.2247449914
H -3.5455978419 -3.1288673384 -4.4805741676
H 1.1256055021 -5.2731559444 -3.1658834689
H -0.4709556827 -5.3988461514 -2.3658994329
H -0.3210390108 -4.5320451595 -3.9156225756
H -3.7275052355 -2.5864166691 -2.2156278624
H 1.080471457 -1.6307775627 -1.0383637252

17b_t2
C -0.5867575517 1.67633881 0.4579142643
N -0.21089744 2.724070143 1.0722079782
N 0.267975712 0.6224629175 0.1018363858
C -1.2608948494 3.7044251662 1.3745112696
C -2.6346690771 3.0559545562 1.5649330127
H -0.9657938304 4.2452810581 2.2789081815
H -1.2968418079 4.4444501683 0.5625992816
C -3.0110116587 2.1908984981 0.3614937686
H -2.61827361 2.4385695412 2.4704107971
H -3.4040741581 3.8215815385 1.7008151708
N -1.8675402099 1.3281557822 0.0140877982
H -3.8633489403 1.5519134576 0.5863793671
H -3.2701409384 2.8054022119 -0.5043521261
C 1.7104546193 0.5877187936 0.3752996033
H 2.1999563442 0.1689038369 -0.5056692126
H 2.0283310623 1.624888629 0.497377934
C 2.0805650936 -0.228135181 1.5680104359
C 2.6148575296 -1.4754395921 1.7102956285
C 2.7439041182 -1.7070516936 3.1199203189
C 2.2759799981 -0.5844472878 3.7310150366
O 1.8695539203 0.3296044395 2.802448578
H 2.1720003028 -0.2871163439 4.7639995335
H 3.1330744311 -2.5897926066 3.6077387382
H 2.8817879467 -2.1468319598 0.9061880022
C -0.4205407691 -0.3677195879 -0.5376822975
O 0.0631646337 -1.4137081187 -0.9618667099
C -1.8477555108 0.1123566253 -0.6315268859
O -1.1697788187 -3.500808888 -2.4210050718
C -2.7774123774 -0.6362849922 -1.276907342
C -0.8277842401 -3.1797776128 -3.7622837837
H -1.3718432465 -3.8655818704 -4.4196673901
H -1.1104744109 -2.152852713 -4.0374261544

H	0.2474962353	-3.3019784674	-3.9623002157
H	-0.7082253188	-2.8569121494	-1.8533670916
C	-4.1541460837	-0.2855759369	-1.6369375949
H	-2.4401136347	-1.6114008548	-1.6133192855
O	-4.8162279967	-1.3959666032	-2.0405526329
O	-4.666411627	0.8243585518	-1.639388272
C	-6.1710361308	-1.1915168334	-2.4808498119
H	-6.5393960403	-2.1777328142	-2.763789959
H	-6.7829380471	-0.7776988113	-1.6751650176
H	-6.2022606224	-0.5165658067	-3.3402422298

E-**17b_t2**

C	-0.8846711022	1.4990852511	0.3349967402
N	-0.6403559542	2.5826322419	0.9568515647
N	0.0777528055	0.5728981176	-0.0744679577
C	-1.80298959	3.4282638425	1.2585635214
C	-3.1133433625	2.6448969164	1.3950859499
H	-1.5937122491	3.9716036249	2.185375076
H	-1.893998417	4.1851094703	0.4665870051
C	-3.3484255952	1.7366221147	0.1858409633
H	-3.0754763857	2.0314529231	2.3023500513
H	-3.9582967348	3.3320008411	1.4953840334
N	-2.1139905736	0.9970319697	-0.0890226089
H	-4.1462001127	1.0184421152	0.3908288896
H	-3.6279238495	2.3152231826	-0.7026785977
C	1.5201433721	0.7100781735	0.152794142
H	2.0279853091	0.4457770345	-0.7766545182
H	1.7021535379	1.7651722102	0.3671886808
C	2.0445573584	-0.1544893468	1.2501576431
C	2.7387340106	-1.328903745	1.2569569228
C	2.958893928	-1.6666886275	2.6335596043
C	2.3792165103	-0.6744946181	3.3629577613
O	1.818889752	0.25790882	2.5383536789
H	2.284962178	-0.4869089457	4.4221794733
H	3.478165929	-2.5311347688	3.0230748518
H	3.0498167404	-1.8869944433	0.3850661001
C	-0.4860673126	-0.4681561355	-0.781495797
O	0.140001102	-1.3849625056	-1.2846691166
C	-1.9740194473	-0.1766400933	-0.7976525323
O	0.4833609638	-4.2323223037	-1.951034289
C	-3.0080091384	-0.8300419279	-1.3903749264
C	1.0545398436	-4.0144826289	-3.2261221281
H	1.7856848072	-4.8100383962	-3.4097039901
H	0.3118051821	-4.0501539912	-4.0392147879
H	1.5823532176	-3.0507644718	-3.2982059195
H	-0.1558386885	-3.5128794291	-1.8124440394
C	-2.9835816546	-2.0926384348	-2.1292931524
H	-3.9886981046	-0.3733051289	-1.3238478082
O	-4.2079597347	-2.298772305	-2.6811070938
O	-2.0709604423	-2.8933444683	-2.2654328836

C	-4.3583539071	-3.5175088539	-3.4292581258
H	-5.3896824796	-3.5192737615	-3.7828964545
H	-3.669828573	-3.5407772373	-4.2781542197
H	-4.1760211385	-4.3889992811	-2.7948417066

18b_t1

C	-0.9805291263	1.0993428396	0.3264164719
N	-0.4071339336	2.0401139189	0.9758359514
N	-0.1712614855	0.0612678326	-0.19171752
C	-1.2104657143	3.1144426665	1.549809238
C	-2.6437048792	2.6817276643	1.8469033097
H	-0.7127191187	3.4566098232	2.4638921448
H	-1.2121966484	3.9702098945	0.8593244346
C	-3.2486819377	2.0509074374	0.6023945806
H	-2.650403851	1.9582486021	2.6702916029
H	-3.2571765026	3.5355449016	2.149852672
N	-2.3582619424	0.9929809916	0.0719775674
H	-4.2112331279	1.5826515852	0.8063156335
H	-3.4013514539	2.8014868411	-0.1814972222
C	1.2894183063	0.2323025685	-0.0316811129
H	1.7763918198	-0.387097403	-0.7837797798
H	1.5251405345	1.2747765854	-0.2473584991
C	1.7902498052	-0.0900977606	1.3427338096
C	1.628745611	-1.1401654379	2.1993342927
C	2.4170327477	-0.8436751516	3.3609384642
C	3.0086028844	0.3584992108	3.1217933898
O	2.6352231292	0.8335961556	1.8975582367
H	3.6873006514	0.9821451892	3.6844366086
H	2.5284688057	-1.4491263991	4.2498889806
H	1.0300024058	-2.0197922851	2.0164798922
C	-0.720678283	-0.9524338766	-0.9369510162
C	0.1418742183	-2.1227078558	-1.3404489177
C	-2.9397923139	0.0121731165	-0.7204729227
C	-2.0472263483	-1.0028081557	-1.2236000872
O	-4.1602685254	0.0388319725	-0.9529137582
H	-2.4727368814	-1.807239784	-1.8062947361
O	-0.160123678	-2.5371953946	-2.5713338456
O	0.959587026	-2.647979266	-0.6105785025
C	0.5237810787	-3.7329892722	-3.0213992285
H	0.1580904606	-3.9091137561	-4.0318240811
H	1.6033800985	-3.5705178864	-3.0268615306
H	0.2781287101	-4.5759151424	-2.3721175787
H	-4.7096961773	-1.3358934174	-2.0200065573
O	-4.83560768	-2.0545879602	-2.6731932779
C	-4.8634563199	-1.4417185642	-3.9521794387
H	-4.962474586	-2.2322547188	-4.7037322035
H	-5.7156447192	-0.7549584707	-4.0741717312
H	-3.9437240594	-0.8797908389	-4.1761057337

18b_t2

C -0.6204650259 1.0724900419 0.2266228497
N -0.3294763278 2.0841060348 0.9537793918
N 0.4213817109 0.1825744844 -0.1205110043
C -1.3837587443 3.0034456402 1.3656418726
C -2.7401545924 2.3141753846 1.4542953939
H -1.1013863813 3.430709179 2.3338958928
H -1.4337207074 3.8428744911 0.6571081942
C -3.0373090227 1.6170475768 0.1358905399
H -2.7338934054 1.584077206 2.2716336991
H -3.538861641 3.0342821143 1.6557694091
N -1.8988023046 0.7429326777 -0.2623151357
H -3.9251568069 0.9920675642 0.22404969
H -3.2103768921 2.3471284667 -0.659065452
C 1.7744438153 0.5036705792 0.3534106398
H 2.4636245992 0.0175364881 -0.3397232487
H 1.9120658992 1.5830169001 0.2975521324
C 2.0254664906 0.0465448542 1.7591202926
C 1.6440317496 -1.0389760797 2.4917796644
C 2.2863684662 -0.9123515399 3.7703056337
C 3.0165723781 0.2337300078 3.7176865756
O 2.8671556267 0.8330654199 2.4970976636
H 3.6599072658 0.7438486364 4.4188921379
H 2.2101829057 -1.5877109728 4.611236729
H 0.9868404594 -1.8327843956 2.1658956638
C 0.255365853 -0.9726166152 -0.873722098
O 1.2189788765 -1.7235487281 -1.1019386804
C -2.0864724865 -0.3593831532 -1.0599179965
C -1.0797353266 -1.2172843828 -1.3628157582
C -3.422554665 -0.5476761998 -1.7358110766
O -3.7749247147 -1.8344139606 -1.7535262948
O -4.0549735583 0.3561225039 -2.2442527683
C -4.9903208539 -2.1456408189 -2.4781632558
H -5.1163063209 -3.2226662565 -2.3784454892
H -5.838176128 -1.6180402389 -2.0365174285
H -4.8863934784 -1.8672915743 -3.5290294764
H -1.2370685492 -2.083765933 -1.9891512158
H 0.7857252944 -3.1306045628 -2.1871909666
O 0.4335050859 -3.7562990694 -2.8527139702
C 0.8860191396 -3.301571277 -4.1181470095
H 0.4672741056 -3.9628724859 -4.8841661429
H 0.5580591999 -2.2755407387 -4.3448849547
H 1.9827680115 -3.3330572686 -4.211518643

S7.4. B3LYP Optimized geometries (from 1c tautomers)

1c_t1

C 1.3094437249 0.2518891738 0.9728995236
N 2.5836556956 0.6275707707 0.5844909687

N	1.0798136485	-0.9840465936	1.3058930774
N	0.3618558878	1.2589974127	0.9778224592
C	0.6112821587	2.7072531712	0.8906344968
H	-0.4861618179	1.0292788301	1.4784364074
H	2.6434228565	1.4645877777	0.0193539722
C	3.6340839968	-0.3568872495	0.3005641838
C	-0.2536923306	-1.3178161041	1.7926913481
C	1.2764850024	3.274394612	2.1523160012
C	-0.7236152065	3.3966217365	0.5979960559
H	1.2685385667	2.8884410115	0.0318989428
H	3.5514611827	-1.1227028842	1.0765033816
C	3.4614525428	-1.0297551215	-1.068368491
C	4.99318228	0.3366990707	0.4213648483
H	-1.0362831674	-1.0645061792	1.0547928117
H	-0.5216399157	-0.7744025208	2.7146924898
C	-0.3681083785	-2.7738307372	2.1110724455
H	4.2516954958	-1.7697167943	-1.2383801298
H	2.497948913	-1.5432467844	-1.13008884
H	3.5127581755	-0.2886753864	-1.8752750468
H	5.8052941682	-0.377023174	0.2501907754
H	5.0917265295	1.1384857805	-0.3212836765
H	5.1235820453	0.7763686545	1.4154280238
H	-0.5793659555	4.4753091824	0.486675254
H	-1.1725658641	3.0098122447	-0.3221395511
H	-1.4315438484	3.2351066731	1.4202495338
H	1.4595141204	4.3487676007	2.0404904512
H	0.6302196497	3.1265132399	3.0252869306
H	2.2351999458	2.7849650592	2.3458326892
C	-0.5269326825	-3.4705638632	3.2736713173
C	-0.5752241241	-4.8624810765	2.9205658515
C	-0.442097554	-4.9112066536	1.567653521
O	-0.3165888221	-3.6495615075	1.0570561351
H	-0.4177896338	-5.7173633468	0.8493231229
H	-0.6926101387	-5.7068842883	3.5857434258
H	-0.5993731467	-3.0425667369	4.2641732896

1c_t2

C	-0.8467828495	0.042184089	-0.4712704657
N	-1.4490860278	0.1820459512	0.6660835912
C	-1.0662536524	1.2517535864	1.5858097982
N	0.0823623398	0.9122601244	-1.0207394099
C	1.1244360211	0.5493765099	-1.9994735405
N	-1.1352085951	-1.0675914232	-1.2603861438
C	-2.2375025248	-1.9493798334	-0.8768139957
C	-2.2603531062	-3.1816345979	-1.7156311061
H	-2.1060525942	-2.2045606506	0.1784904978
H	-3.2176168028	-1.4599800186	-0.9631608909
O	-1.2209364084	-4.0655662177	-1.5700723192
C	-1.4574590662	-5.1113237144	-2.4159840521
C	-2.6204600992	-4.9190116295	-3.0953891967

C	-3.1422596584	-3.6613541823	-2.6400537996
H	-4.0548117254	-3.1757306544	-2.9574562495
H	-3.0545468743	-5.5848449146	-3.8282825818
H	-0.7161992496	-5.8967492329	-2.416140124
C	-1.328482809	0.7731238493	3.0190090131
H	0.0112610259	1.4880526629	1.5169637285
C	-1.8493115015	2.5424686025	1.2907008936
H	0.3833882358	1.6345398898	-0.378724054
C	1.737001054	1.8463874007	-2.5331979043
C	2.1930076706	-0.3836719172	-1.4146647261
H	0.6391199046	0.0409072923	-2.839336932
H	2.9423588864	-0.6336798516	-2.1739485185
H	2.706241596	0.0973529125	-0.5737960002
H	1.7472381022	-1.3174938289	-1.0598659209
H	-1.0468669289	1.5382416129	3.7511017194
H	-2.391272275	0.5408543883	3.1560234859
H	-0.7560531009	-0.1352891699	3.2356674206
H	-1.5983495817	3.3305731154	2.0099258646
H	-1.6313101552	2.920467261	0.2861970718
H	-2.9273589757	2.3529017762	1.3528702453
H	-1.080127825	-0.9060850076	-2.2589588209
H	2.4910022926	1.6275605333	-3.2951851006
H	0.9705800156	2.4888076059	-2.9780749447
H	2.2269722429	2.4054116808	-1.7264055323

NCov(1a_t1)

C	-0.8965676164	2.4295161805	-0.3954149235
N	-1.8338104795	1.6033609524	0.1970250936
N	0.3238440802	2.0089014927	-0.5644911021
N	-1.3554659401	3.6768667839	-0.7753070835
C	-2.7607433857	4.1000727217	-0.8904076626
H	-0.7335471989	4.1788023238	-1.3945490909
H	-2.6344826185	2.0704450495	0.6027309478
C	-1.4664447031	0.3548813632	0.8740969946
C	1.2450560682	2.8879771247	-1.2776189359
C	-3.4870525592	3.4345701462	-2.0673641321
C	-2.7854743346	5.6263012671	-1.0006013529
H	-3.2736046566	3.8294390544	0.040182734
H	-0.7118839275	-0.1251713162	0.2466516259
C	-0.8664574242	0.5918438751	2.2670925915
C	-2.7023580959	-0.5458105376	0.9392147186
H	1.3600148379	3.8611989551	-0.7675833433
H	0.9058657534	3.1219788055	-2.3007677115
C	2.5992522692	2.2706750472	-1.4122609104
H	-0.5713450508	-0.3572916679	2.7272146122
H	0.0195840971	1.230054831	2.2080392769
H	-1.5971894223	1.0757745713	2.9266195184
H	-2.4510956369	-1.5061984122	1.4005920811
H	-3.4955799086	-0.0833674087	1.5399913239
H	-3.1019282039	-0.738316869	-0.0616890166

H -3.8167049626 5.9881077796 -1.0503230831
H -2.2960442788 6.0906862048 -0.1388568141
H -2.2687436856 5.958110413 -1.9095590471
H -4.5322814374 3.7602381647 -2.1079239146
H -3.0075050491 3.7043316499 -3.0154393404
H -3.4742784858 2.3451045609 -1.9719787427
C 3.3115698074 1.7911267621 -2.4727202316
C 4.5640217068 1.3174624654 -1.952622516
C 4.5234844352 1.5423793922 -0.6113726988
O 3.336226643 2.1252761546 -0.2637480637
H 5.2188403989 1.3654783182 0.1957842683
H 5.3801567278 0.8723165029 -2.504895695
H 2.9818916749 1.7755974172 -3.5025415632
C 0.5799850265 -4.0458816395 1.5922217549
C -0.3212549111 -4.8426583331 1.7332135154
C 1.7112333994 -3.1427436977 1.4754010194
C -1.3905317642 -5.8213322604 1.8122652068
O 1.3424107439 -1.9832344028 0.9274093426
O 2.8318178666 -3.4368151464 1.8417283668
C 2.3893495661 -0.9893098441 0.7718310218
H 1.8929582853 -0.1192283 0.3476214294
H 2.8271507894 -0.7512089652 1.7433785776
H 3.1623330945 -1.3640219307 0.0978653472
O -2.0563406443 -5.71254515 2.9644455187
O -1.6224768179 -6.6192970212 0.9258082504
C -3.1517745316 -6.6445676996 3.1470662899
H -3.5679613647 -6.4078304355 4.1250598818
H -3.9021158015 -6.5034556463 2.3664352512
H -2.7815713741 -7.671652647 3.1261834148

NCov(1a_t2)
C -1.8882660798 0.604871229 -0.0864246531
N -1.1938686497 1.5484846114 -0.6415719291
C -1.3902003863 2.9402031722 -0.2332572537
N -2.7404136884 0.747959059 0.9963993097
C -3.8593053619 -0.149106776 1.3384361341
N -1.8017584155 -0.6912652781 -0.582520188
C -0.8044371003 -1.0079207109 -1.6041143053
C -0.9764912407 -2.3993134079 -2.1109102208
H -0.9215660934 -0.2882893102 -2.419443587
H 0.2242695797 -0.9039948876 -1.2345172498
O -2.1084081288 -2.673283986 -2.8366066241
C -2.0456513573 -3.9872356931 -3.2035295197
C -0.9036610591 -4.5578405573 -2.7328618593
C -0.208362217 -3.5236856483 -2.0200007201
H 0.7400698133 -3.6036853918 -1.5067466675
H -0.5926420475 -5.5837857538 -2.8726833743
H -2.8769119008 -4.3562670441 -3.7859967708
C -1.0315965175 3.8506230749 -1.4138936394
H -2.4474695684 3.1418966283 0.0184127623

C	-0.5377330038	3.2927700497	0.9987282335
H	-2.9145661352	1.7114294556	1.2514549082
C	-4.3501896322	0.2283608223	2.7378828841
C	-4.9957692478	-0.1066772752	0.3082563617
H	-3.4708324571	-1.1722239542	1.3888105529
H	-5.8011683939	-0.7913075038	0.5965706116
H	-5.4149350561	0.9038692285	0.2385957574
H	-4.6385350448	-0.4028040506	-0.6822205554
H	-1.2090724379	4.9038019675	-1.1683843843
H	0.0256858437	3.7352947936	-1.6788783885
H	-1.6318917332	3.5989328568	-2.2948670696
H	-0.6677796658	4.3446446187	1.2785619099
H	-0.8088762619	2.6786706723	1.8639229788
H	0.5232562312	3.1250146175	0.783167789
H	-1.9135409969	-1.4221520774	0.1096426349
H	-5.1562382532	-0.4408944271	3.0530529453
H	-3.5389702017	0.1628187479	3.4697530301
H	-4.7406734478	1.2534482369	2.7493214058
C	3.1915474714	0.7551580997	0.5036645353
C	3.5245668107	0.3167197046	1.5823563474
C	2.7909662666	1.367970774	-0.7493299414
C	4.013037368	-0.220989865	2.8393734469
O	2.5689984325	0.4458085674	-1.6882288502
O	2.6905473963	2.5712129794	-0.891881962
C	2.1342925921	0.9513004486	-2.9771035474
H	2.0307696654	0.0698772929	-3.6079151635
H	1.177121268	1.4644852446	-2.8704588898
H	2.8831188979	1.6316442894	-3.3873883419
O	3.0133200622	-0.4041817032	3.7047607188
O	5.1867764583	-0.4609005352	3.0421247469
C	3.3918526629	-0.9446000306	4.995891887
H	2.4620574303	-1.0186680511	5.5577026089
H	3.8462662459	-1.9301543758	4.8743782274
H	4.0906322855	-0.2717289487	5.4972399284

TS1(1c_t1)

C	-0.9423933577	1.0133482227	0.1039607118
N	-1.4171359916	0.8871559119	1.3723792418
N	0.2944230661	0.6315925074	-0.1819437011
N	-1.7938210734	1.5209909522	-0.8345413812
C	-3.0999746773	2.1637782888	-0.6271398422
H	-1.5536573116	1.3381359609	-1.7992916234
H	-2.4069526995	1.023798028	1.52073458
C	-0.6100892968	0.7238567424	2.5874561476
C	0.817540426	0.9614712953	-1.5085710933
C	-4.2500722126	1.1502464817	-0.7167812238
C	-3.2597014775	3.2923523971	-1.6491901575
H	-3.0859689983	2.6069424292	0.3738683779
H	0.4167317577	0.5732262849	2.2543036686
C	-0.6860297672	1.9917679262	3.4481494263

C	-1.0756979011	-0.5129321335	3.3630306731
H	0.4965560069	1.9711838501	-1.8058510146
H	0.4477753151	0.2729229629	-2.2838205104
C	2.3097046341	0.9128270708	-1.5553224129
H	-0.0799555284	1.8777385402	4.3534762335
H	-0.3177236477	2.8637959815	2.8981617073
H	-1.7187014236	2.1932181904	3.757792098
H	-0.4579640228	-0.660598867	4.2550880416
H	-2.1161632692	-0.3990720829	3.6914560277
H	-1.0102310048	-1.4122527856	2.7441573637
H	-4.2182138629	3.8004728636	-1.506677729
H	-2.4576889526	4.0299300091	-1.5485276229
H	-3.2378801249	2.8957117913	-2.6713085222
H	-5.2098798957	1.6422566597	-0.5270537216
H	-4.2868657592	0.7027602864	-1.7165351492
H	-4.129859829	0.3436488032	0.0138621056
C	3.1987298896	0.1292574997	-2.232576738
C	4.5110795767	0.6006996015	-1.8877267076
C	4.3225711884	1.637121014	-1.0258621884
O	2.9892424003	1.8458973909	-0.8169186109
H	4.9941438109	2.3023658261	-0.5034368205
H	5.4605575376	0.2177628771	-2.2354810907
H	2.9479913074	-0.6877786796	-2.894933783
C	0.6094318007	-1.4281441054	0.0810220842
C	-0.3005131773	-2.18741434	-0.3204712812
C	1.9676838961	-1.5399187077	0.6423901527
C	-1.5437362128	-2.5128007364	-0.9305397923
O	2.3497935854	-0.512588065	1.4100096998
O	2.6561443462	-2.5273332463	0.4479542559
C	3.663750636	-0.6304993265	1.9933446765
H	3.8217263864	0.2984369038	2.540516964
H	3.7063674552	-1.4835093426	2.6754289703
H	4.4211105069	-0.7470741504	1.215836537
O	-2.5118493429	-2.7587799448	-0.0094167309
O	-1.7229722926	-2.6089256082	-2.13999519
C	-3.7899417717	-3.1460857365	-0.5476565245
H	-4.4347092613	-3.3039491743	0.3173567464
H	-4.2030105535	-2.3596686687	-1.1845573777
H	-3.7060278321	-4.0701608497	-1.1259949503

TS1(1c_t2)

C	-0.5019641214	0.7295759258	-0.1495849302
N	-0.8137856799	0.152243205	0.9976846996
C	-1.9183853266	0.6986037352	1.809576201
N	-1.2824533624	1.6645995421	-0.7722377732
C	-0.8500828547	2.6740648325	-1.7574534807
N	0.6394460553	0.3861952783	-0.8069928077
C	1.7574785831	-0.366491553	-0.2507124215
C	3.0689170331	0.255698076	-0.599238231
H	1.6220403362	-0.409295396	0.8320377627

H 1.7561822698 -1.3952926188 -0.6262789975
O 3.338367757 1.496676982 -0.0779013903
C 4.5834989703 1.8556396252 -0.5116734221
C 5.1150526956 0.8756175914 -1.2911763299
C 4.1272960536 -0.1648270525 -1.3503539768
H 4.1942866275 -1.1045469731 -1.8811011793
H 6.0856995697 0.8887762051 -1.7671962885
H 4.9386223976 2.8213817449 -0.1836174988
C -1.5974818964 0.6040521741 3.30770645
H -2.0084009069 1.7712900718 1.5878918113
C -3.2701739854 0.0356015572 1.4975585542
H -2.184636887 1.8370292782 -0.3535173674
C -2.1078204068 3.3191986654 -2.3413271021
C 0.0969495475 3.714062103 -1.1467769931
H -0.3315828653 2.1629194472 -2.5770762663
H 0.4055909315 4.4367811038 -1.9096922158
H -0.4013524256 4.2590762381 -0.3373010248
H 0.9980418956 3.2422617302 -0.7446925595
H -2.336698416 1.1780971357 3.8768387768
H -1.6375667174 -0.4263736818 3.6715807958
H -0.6065261498 1.013439797 3.5239647546
H -4.0693332032 0.514863377 2.0737274278
H -3.52578295 0.1124377248 0.4355112811
H -3.2527393809 -1.0264507265 1.7621942263
H 0.7019488713 0.6149733024 -1.7884977899
H -1.8343533041 4.0492544004 -3.1081449358
H -2.760937811 2.5682187216 -2.7968935294
H -2.6736040222 3.8430832301 -1.5615965117
C -0.7050363391 -1.8785243466 0.9428388946
C -0.8844170892 -2.4989741513 -0.131939429
C -0.3807427799 -2.2364171447 2.3353702694
C -1.0011994249 -2.6217599328 -1.5423989002
O 0.7421831297 -1.6474053192 2.7756534403
O -1.0306367339 -3.0297986235 2.9927915947
C 1.1505497561 -1.9960980778 4.1151865203
H 2.0724921483 -1.4423169654 4.2907949465
H 0.388029817 -1.7007224502 4.8397403257
H 1.3323845845 -3.0704368846 4.1944070585
O -2.2999614214 -2.6646465583 -1.9456195604
O -0.0552731592 -2.7339546299 -2.3180010969
C -2.5002826155 -2.8439482462 -3.359097346
H -3.5813453308 -2.8693574301 -3.5005446955
H -2.0690958181 -2.0142166277 -3.9259131143
H -2.0556786454 -3.7819574113 -3.7024516254

10c_t1

C 1.1357824444 -0.7699574542 0.2217579543
N 1.4968028488 -0.8380484075 1.5132304214
N -0.0465948505 -0.2279701074 -0.1703393839
N 1.9833065116 -1.2547227997 -0.7081770816

C	3.1532931377	-2.1279983744	-0.492367256
H	1.8952953889	-0.8836807361	-1.6459954854
H	2.4825929382	-0.989360049	1.6853830374
C	0.6595589685	-0.8509998344	2.727174727
C	-0.4897978127	-0.4282903764	-1.5802150473
C	4.4481462842	-1.3082032366	-0.447350188
C	3.1818682597	-3.187089449	-1.596816714
H	3.003498091	-2.6291962816	0.4681942849
H	-0.3767799008	-0.7840673336	2.4013850255
C	0.8581005707	-2.185468958	3.4548682864
C	1.009494378	0.3440441691	3.6182568699
H	-0.1227647612	-1.3992760786	-1.9199933674
H	-0.0450753574	0.3520620394	-2.2084599921
C	-1.9661412677	-0.3935416408	-1.7680650196
H	0.2425087728	-2.2140560854	4.3596656223
H	0.5729258515	-3.0280960166	2.8173086006
H	1.9046897805	-2.3162675963	3.7542570271
H	0.3665097079	0.3551966475	4.5040575999
H	2.0500796068	0.2802727833	3.9577983131
H	0.8792014541	1.2862071047	3.0801691905
H	4.0358864702	-3.8553941583	-1.4519646597
H	2.26696447	-3.7870379043	-1.5902020698
H	3.2830519303	-2.7185665199	-2.5826947959
H	5.3021760934	-1.9621960881	-0.2451162234
H	4.6192749402	-0.8086324317	-1.4075376382
H	4.4092373687	-0.5413510161	0.3328034418
C	-2.8314931799	0.5749827593	-2.1883864524
C	-4.1382279839	-0.0158297274	-2.1912168306
C	-3.9695244607	-1.3025590827	-1.7776015322
O	-2.6540346576	-1.5531535784	-1.5235368604
H	-4.6465326432	-2.1315075243	-1.6331796028
H	-5.0718432715	0.4536916885	-2.4680322522
H	-2.5668509927	1.5877653095	-2.4587185248
C	-0.552162985	1.0138455429	0.4732431072
C	0.0829967766	2.191274454	0.4106274361
C	-1.8809181218	0.8990282959	1.1399940058
C	1.3580536367	2.4147879364	-0.1958545855
O	-2.3113592165	-0.3857496485	1.2519889625
O	-2.5388661065	1.8339967012	1.5643908968
C	-3.5993345	-0.5588396833	1.8658765678
H	-3.8029175588	-1.6293773223	1.8271757748
H	-3.584859782	-0.2169211935	2.9045564761
H	-4.3684041237	-0.0090033826	1.3186657276
O	1.2474067881	2.7728493014	-1.5244014986
O	2.4492937979	2.4292220663	0.3800822607
C	2.4653888423	3.2002100091	-2.1489576194
H	2.1985167162	3.459207847	-3.175357793
H	2.8864486289	4.0758266981	-1.6452995696
H	3.2157400794	2.4036937232	-2.1533045738

10c_t2

C	0.0316275224	-0.8558980099	-0.0685966063
N	1.2804770928	-0.5220895864	-0.4677038478
C	1.9803907276	-1.2662114357	-1.5593334803
N	-0.4029636695	-2.1228082139	-0.210676633
C	-1.7758168238	-2.5921007046	-0.5000654968
N	-0.7542885446	0.0984786334	0.4697295499
C	-1.6655467519	-0.0659075109	1.6103361512
C	-2.9811542041	0.6071114634	1.405947915
H	-1.7934265534	-1.1307495811	1.8130813542
H	-1.2053830605	0.3787530129	2.4999719179
O	-3.8074259696	0.1124928378	0.4292595779
C	-4.9433137816	0.8726789766	0.4360230979
C	-4.8617195928	1.8347806893	1.3939971138
C	-3.5830039227	1.6636921789	2.0234462577
H	-3.1639393877	2.2498635765	2.8296039649
H	-5.6134146965	2.5767705266	1.6243190719
H	-5.6960121262	0.604127985	-0.2902759858
C	3.1157198657	-2.1561694528	-1.0419882736
H	1.2199225175	-1.9061504913	-2.0124621697
C	2.4701161335	-0.3021511644	-2.6438581612
H	0.317963963	-2.817838471	-0.3638575798
C	-1.8689341539	-3.0502479919	-1.9600603627
C	-2.1534100682	-3.7142403519	0.4697408724
H	-2.4436636971	-1.7422397505	-0.3567218521
H	-3.1729104451	-4.0569847945	0.2684189077
H	-1.4795361605	-4.5707601394	0.3513190292
H	-2.1011397435	-3.3810621844	1.5106159121
H	3.5339732221	-2.7246832974	-1.8789286954
H	3.9254902089	-1.5617053759	-0.6069692861
H	2.7636272135	-2.8633779287	-0.2865426596
H	2.8995690225	-0.883155557	-3.4665998491
H	1.6481882417	0.2994655969	-3.0407192881
H	3.2402876472	0.3756401131	-2.2666545343
H	-0.5015798304	1.0669632092	0.235798223
H	-2.8827087243	-3.4022013791	-2.1771214996
H	-1.6368460841	-2.2319828234	-2.6484142598
H	-1.1754949383	-3.8764279413	-2.1551621133
C	2.0211173482	0.5322285876	0.2582296555
C	2.0009556249	1.8390445685	-0.0281166488
C	2.7921357255	0.0406912674	1.4415612648
C	1.1177222966	2.4598717672	-0.9519018124
O	2.3806114615	-1.1850581854	1.8588198554
O	3.6676812194	0.658840942	2.0224393303
C	3.058466483	-1.7079761552	3.0137368571
H	2.6089814682	-2.6831028583	3.2043442308
H	4.1287866795	-1.8186934465	2.8204674407
H	2.9173027828	-1.0535959947	3.8780412635
O	1.7225125323	2.9867165679	-2.0554367664
O	-0.0954616218	2.6502072864	-0.7322622768

C 0.8673428896 3.7139290761 -2.9502119931
H 1.5043174086 4.0221620118 -3.7809457538
H 0.0514109461 3.0876223405 -3.3222316001
H 0.4426513075 4.5966915624 -2.4632803291

TS2(1c_t1)
C 0.9788304484 -0.7834837963 0.3562806014
N 1.4116149582 -0.6701910588 1.6212647075
N -0.197209762 -0.2372612902 -0.0536473577
N 1.7407744476 -1.4613493775 -0.5237733921
C 2.8768716597 -2.3563177596 -0.2285777847
H 1.604625892 -1.2539596935 -1.5048140489
H 2.382574207 -0.9116710915 1.7752639397
C 0.6528227994 -0.4034411166 2.8584464243
C -0.7081547761 -0.6062780542 -1.4025598626
C 4.2120484433 -1.6113320771 -0.3464215895
C 2.8056384588 -3.5582519492 -1.1729773486
H 2.7448361603 -2.7115200046 0.7976982892
H -0.3671576475 -0.1621747371 2.5670449483
C 0.6282181082 -1.6691636621 3.7228310818
C 1.2823290181 0.7830972 3.5936073935
H -0.4299828066 -1.643919553 -1.6001892637
H -0.2320626184 0.0302258728 -2.158910489
C -2.1837900986 -0.485071374 -1.5595779238
H 0.0721334311 -1.4801085419 4.6468414236
H 0.1463920941 -2.4984428126 3.1955563087
H 1.6444438949 -1.9760036905 3.9963782577
H 0.6892868026 1.0322439323 4.4793907688
H 2.2953587556 0.5302865087 3.9300769092
H 1.3465638334 1.6648133395 2.9488572994
H 3.6291091945 -4.247083535 -0.9630055185
H 1.8619844891 -4.0985161286 -1.0522080493
H 2.891775058 -3.2376527101 -2.2177680202
H 5.0405511961 -2.283909308 -0.1029800992
H 4.357390978 -1.2442053986 -1.3684006433
H 4.2558404061 -0.7544733899 0.3333796375
C -2.9869304247 0.4565291438 -2.1356772869
C -4.3330287605 -0.0234527842 -2.0157146782
C -4.2479510295 -1.2250615512 -1.3802071252
O -2.9479095359 -1.5266124866 -1.1020710694
H -4.9798710404 -1.959961567 -1.0791806208
H -5.2366966811 0.4597090964 -2.3600929843
H -2.6564531812 1.3814449409 -2.587650763
C -0.5730733439 1.1367447744 0.4087524329
C 0.2259353716 2.1645281793 0.2675724797
C -1.9342309117 1.24609454 1.0286651969
C 1.1302909073 3.1892098711 0.2506648654
O -2.4299761466 0.050959696 1.4346393905
O -2.5346376508 2.291774455 1.2032412271
C -3.7257869416 0.0927937042 2.0589909296

H	-3.9830403209	-0.944660047	2.274175187
H	-3.6902439716	0.6710267387	2.9863406961
H	-4.4661432922	0.5340176749	1.3882698884
O	1.0884721809	3.927932148	-0.9343292057
O	1.9316572866	3.4902364918	1.163778528
C	1.9959331143	5.0278544897	-0.9853356281
H	1.8430759218	5.4932602544	-1.9622594157
H	1.7951345549	5.7635478863	-0.1986467703
H	3.0390768699	4.7051576084	-0.8969198734

TS2(1c_t2)

C	-0.1756082635	-1.1026548683	0.1198078412
N	1.0657848002	-0.7830344228	-0.2940175929
C	1.7929362574	-1.5979986296	-1.3154029117
N	-0.6207231425	-2.3730629518	0.0339251001
C	-1.9954897017	-2.8362948769	-0.2657527691
N	-0.9719397145	-0.1309799625	0.6223343927
C	-1.8766157412	-0.2641136086	1.7765482486
C	-3.1708730155	0.4465309712	1.5721447049
H	-2.0338779929	-1.3242250867	1.9805624365
H	-1.3949965272	0.1687749291	2.6599723715
O	-4.0149947652	-0.0348037098	0.6044055751
C	-5.1265148792	0.7603619857	0.6055121347
C	-5.0116203773	1.7317530343	1.5507762097
C	-3.7366439771	1.5291781206	2.178312949
H	-3.2965370113	2.1124215723	2.9752759219
H	-5.7389798347	2.4997496144	1.7739200451
H	-5.8898214224	0.5069753687	-0.1152382683
C	2.9371945495	-2.4114404267	-0.7024163542
H	1.0537833327	-2.2919287792	-1.7216271185
C	2.2761015767	-0.7267069103	-2.479505157
H	0.093316898	-3.0761212056	-0.1125590877
C	-2.0799822743	-3.3063540267	-1.7225040952
C	-2.3848359408	-3.9476705997	0.7111086946
H	-2.6609683524	-1.9824232199	-0.1355319191
H	-3.4034279782	-4.2896008948	0.5041252708
H	-1.7123052865	-4.8070134189	0.6063768974
H	-2.3404957816	-3.6045442586	1.7491434752
H	3.3808411473	-3.0473442828	-1.4755335648
H	3.7248683731	-1.7612819249	-0.3099414524
H	2.5839666737	-3.0526149846	0.1099381717
H	2.6705434265	-1.3810064958	-3.2637467085
H	1.4573951327	-0.1380093614	-2.9028551945
H	3.0729837971	-0.0425726455	-2.1767591741
H	-0.6218992849	0.8096532625	0.4713120182
H	-3.0943748089	-3.6537211226	-1.944167699
H	-1.8376231137	-2.4960036809	-2.4165598382
H	-1.390507127	-4.1387416088	-1.9045066242
C	1.6886186674	0.4788027839	0.1841724618
C	1.4292164206	1.6400175377	-0.36851297

C 2.584874765 0.3246907847 1.3789634794
C 1.0362016592 2.877598424 -0.7922706844
O 2.4071732373 -0.8557098922 2.0198996959
O 3.3662396675 1.1717542425 1.77447667
C 3.2166154198 -1.0551448729 3.1929997529
H 2.9478020106 -2.0409729595 3.5735212134
H 4.2797790369 -1.026070081 2.9406798586
H 3.005271392 -0.2906095572 3.9451563379
O 1.8273440304 3.3901878662 -1.8190050991
O 0.0605881844 3.5201790697 -0.3429116078
C 1.4032976284 4.6569029775 -2.3228334242
H 2.1076603895 4.9101573985 -3.1189406938
H 0.3890872865 4.6160350733 -2.735038344
H 1.4317855539 5.4349373109 -1.5524435763

11c_t1
C 1.3463368044 -1.275982676 0.0436109793
N 1.7657832628 -1.149476014 1.3124052685
N 0.2487820302 -0.615313759 -0.4158503534
N 2.0270048617 -2.0913106778 -0.7822174402
C 3.0016312092 -3.1378838325 -0.4166916209
H 1.9345738314 -1.9192556117 -1.7754471679
H 2.7103195043 -1.4616574435 1.500172215
C 0.9986820483 -0.7698785866 2.5146488178
C -0.3056488417 -1.0093607281 -1.7397170107
C 4.4398793263 -2.6231655847 -0.5445240926
C 2.7507144013 -4.3634007714 -1.2983942584
H 2.8006327556 -3.4075069174 0.624084929
H -0.0164114874 -0.5490091206 2.1886171243
C 0.9559982659 -1.9648568835 3.4741697315
C 1.6204917031 0.4664500202 3.1702000095
H -0.157293875 -2.0837455504 -1.8682650285
H 0.24412292 -0.484589184 -2.531136205
C -1.7551018804 -0.7130165905 -1.9070115192
H 0.3829888659 -1.7054707916 4.3701716779
H 0.4837176766 -2.8325161833 3.0029504983
H 1.9663925756 -2.2497125575 3.7903819006
H 1.0481423561 0.7422846129 4.0619411788
H 2.6497562134 0.2568685957 3.4856263796
H 1.6336169453 1.3210486803 2.4882855731
H 3.4503006131 -5.1620967819 -1.0348472556
H 1.7312719411 -4.7391716603 -1.1697466939
H 2.9005534761 -4.1187555198 -2.3564272563
H 5.1464093836 -3.4066833402 -0.2530183219
H 4.6546855667 -2.3358166457 -1.579765006
H 4.6129678073 -1.7515872005 0.0947250344
C -2.4371113576 0.2929248356 -2.5285665965
C -3.8322966796 -0.0016748965 -2.3747647268
C -3.8948226075 -1.1685804254 -1.6751464879
O -2.6410146113 -1.6205693434 -1.3887403997

H -4.7116816426 -1.7865128051 -1.3323704545
H -4.6706030724 0.574956226 -2.7400681005
H -1.9955875102 1.1424612888 -3.0308187993
C 0.0722097995 0.8094746951 -0.066437229
C 1.0334410376 1.6820873275 -0.3965388642
C -1.2086123468 1.1600465782 0.6091149084
C 1.0087435671 3.0727982394 -0.0482918671
O -1.9182451781 0.0789412483 1.0248970832
O -1.5970684418 2.3003385799 0.8054999666
C -3.1713502941 0.3658877254 1.670032978
H -3.6159813415 -0.6040914346 1.8942179517
H -3.0133714631 0.9294281073 2.5936321264
H -3.8268201799 0.9370209644 1.0086327594
O 0.5709040994 3.8668154413 -1.0800141986
O 1.4865928388 3.5615854347 0.9795700813
C 0.655364745 5.276826078 -0.8477658403
H 0.2993080917 5.7523734031 -1.7640100161
H 0.0245898171 5.5833404145 -0.007139531
H 1.6842404697 5.590750021 -0.6457738302

11c_t2
C -0.6699145895 -0.9122634552 -0.0618201722
N 0.624080166 -1.16187564 -0.3714721516
C 0.9923927054 -2.3418534599 -1.2211063113
N -1.5347320171 -1.9363302668 0.0788690035
C -2.9763583247 -1.9640092712 -0.2547452801
N -1.0642096116 0.3672280962 0.0840802458
C -2.0027927942 0.890145854 1.0876507757
C -2.9440483638 1.8998335651 0.5231318089
H -2.5422807988 0.0560737262 1.5397301375
H -1.4325476291 1.3779919452 1.8856320678
O -3.8744639312 1.4641724196 -0.385525984
C -4.6201374842 2.5489642506 -0.7531119563
C -4.1903525993 3.6611985677 -0.098557152
C -3.0971337377 3.238977813 0.7304106501
H -2.5011721562 3.8505611324 1.393634748
H -4.5952880615 4.6589725222 -0.193882576
H -5.4003081017 2.3632677368 -1.4762513269
C 1.7261617623 -3.4430276409 -0.4497474815
H 0.0434585817 -2.7513203762 -1.573736647
C 1.7810509333 -1.8956816298 -2.4566809407
H -1.113381585 -2.8506735393 0.1919490002
C -3.194214529 -2.7715461377 -1.5395341877
C -3.7679395327 -2.544200077 0.9195379213
H -3.2858783964 -0.9337459612 -0.4332707978
H -4.8363279104 -2.5553662187 0.6832295506
H -3.4561996291 -3.5750724795 1.1240139629
H -3.6225176045 -1.9569323767 1.8312004413
H 1.8576935338 -4.3083202626 -1.1080357062
H 2.7178980825 -3.1186206443 -0.1224407389

H	1.1620306222	-3.7639837684	0.4301997035
H	1.8920908967	-2.7511160546	-3.1309002852
H	1.2591683967	-1.0987785468	-2.9942738887
H	2.780537474	-1.5368822813	-2.1964096933
H	-0.3493751721	1.0229807466	-0.2497591083
H	-4.2604892644	-2.8003171702	-1.786423258
H	-2.6600207278	-2.3256960218	-2.3839342108
H	-2.848337396	-3.8041664677	-1.4146014454
C	1.6622831868	-0.1709098599	-0.0662419757
C	1.6842893257	1.0153403176	-0.6925861792
C	2.5837701452	-0.5447394924	1.0500694246
C	2.6202616169	2.0632868837	-0.3674301619
O	2.04539365	-1.4557556592	1.8971252914
O	3.6829603258	-0.0498443667	1.2383542923
C	2.8697245974	-1.8308757485	3.0148879253
H	2.2889128707	-2.5620424264	3.578057593
H	3.8077456617	-2.2775379989	2.6747178409
H	3.0924382923	-0.9636231607	3.6419793099
O	3.6837254365	2.089189922	-1.2331558897
O	2.4428170743	2.9516480801	0.4650908835
C	4.592626649	3.1820115031	-1.0573873906
H	5.3481462811	3.0704671958	-1.837631535
H	4.0871662799	4.1462328961	-1.1681218951
H	5.0729994003	3.1478892864	-0.0744052505

TS3(1c_t1)

C	0.0019139417	-1.66046243	-0.2782634614
N	-1.0255299899	-2.5429227578	-0.1916520959
N	-0.3124162391	-0.3034362131	-0.3530671063
N	1.263319272	-2.0244892677	-0.3311522926
C	1.7805275989	-3.3512528431	0.0237628266
H	1.9323177382	-1.0263590812	-0.482567973
H	-0.7691915271	-3.5148896118	-0.3177834429
C	-2.3087585274	-2.3400048887	0.5154885245
C	-1.2487896776	0.0858111484	-1.4528121853
C	3.118909973	-3.1764398283	0.7490932919
C	1.9266216895	-4.2298282036	-1.2261172733
H	1.0746923788	-3.8336987475	0.7132490494
H	-2.4191471541	-1.2655422999	0.6663955979
C	-3.4628667463	-2.8527060063	-0.3489796126
C	-2.2737103789	-3.0357238186	1.8815159935
H	-1.7975394292	-0.8109587464	-1.7453620537
H	-0.6520442608	0.4029565628	-2.3162794222
C	-2.2041068948	1.1845536166	-1.1437102577
H	-4.4165049071	-2.7008233607	0.1662615484
H	-3.5062284933	-2.3317960961	-1.3101744703
H	-3.3538463775	-3.925777121	-0.5460567314
H	-3.2228477771	-2.8851597398	2.4065791448
H	-2.1198406187	-4.1149771662	1.7650671775
H	-1.4700647714	-2.6387472305	2.5093250151

H 2.319552642 -5.2151795694 -0.9543429885
H 0.9646260299 -4.3745812369 -1.7290588503
H 2.6180378315 -3.7688672608 -1.9400500251
H 3.507103968 -4.1497965276 1.0645025919
H 3.8614691036 -2.7121873384 0.0904616119
H 3.0060308719 -2.5458028122 1.6364649131
C -2.3129134459 2.464374445 -1.6057484268
C -3.4805649213 3.0264708222 -0.9894381199
C -3.99680698 2.0466496508 -0.1981198503
O -3.2393280873 0.9142855132 -0.2879776687
H -4.858460084 1.9904561504 0.4504058013
H -3.8783634681 4.0232420449 -1.1197063863
H -1.6400390336 2.9465813145 -2.3012924417
C 0.7159595296 0.6756248569 -0.0617350177
C 2.0088431366 0.4202933705 -0.3252349794
C 0.2740000555 1.8982487573 0.6811766864
C 3.1233669031 1.2591510751 0.0873064476
O -0.8014022613 1.6764581737 1.4653194019
O 0.8669831281 2.9636832682 0.6540781709
C -1.240734542 2.8001096758 2.2508451842
H -2.1308619444 2.4590926388 2.7796382216
H -0.4682147672 3.0959336495 2.965952267
H -1.4841817683 3.6512337977 1.6111470119
O 3.5986633618 2.0154784399 -0.9402728558
O 3.6854266608 1.2046656737 1.1753773821
C 4.7743564698 2.7863953397 -0.6478490934
H 5.0214312204 3.3122874752 -1.5714256778
H 4.5837750078 3.5094345281 0.1505409409
H 5.6073445614 2.1421202151 -0.3520960421

TS3(1c_t2)
C -0.3539258542 -1.0405115577 0.1978062057
N 0.9648660634 -0.8792444652 -0.1912200056
C 1.5563556916 -1.8804906615 -1.149908236
N -0.7642777141 -2.3101932909 0.4553394975
C -2.0870480785 -2.9108791707 0.1829689173
N -1.1102327611 0.038750707 0.3022732132
C -2.260989235 0.1953970512 1.192143793
C -3.4788055278 0.7004969189 0.4885542416
H -2.4755745623 -0.73391648 1.7279467518
H -2.0031523296 0.944232972 1.9509374344
O -4.0577860905 -0.113672846 -0.4514877065
C -5.1482906978 0.5466341099 -0.946207758
C -5.2787603553 1.7583694895 -0.3428281447
C -4.1906731113 1.8595853868 0.5887431844
H -3.9655702277 2.6869166971 1.2475371361
H -6.048412661 2.4924395532 -0.5369334915
H -5.7101063554 0.0270760538 -1.7080287364
C 2.6146823468 -2.802052644 -0.531964583
H 0.7148221042 -2.5084821733 -1.4469648045

C 2.0714987885 -1.1913667535 -2.4182981495
H -0.0202377247 -2.9869171731 0.5735251344
C -1.9940859705 -3.8569697061 -1.0205335246
C -2.597275444 -3.6370219486 1.4306033669
H -2.7672611666 -2.0972713237 -0.0699795468
H -3.5816116666 -4.0753441725 1.2382691002
H -1.9154375804 -4.4483568003 1.7113327845
H -2.6837674769 -2.9561124743 2.2829014065
H 2.8124377749 -3.6244587171 -1.227905456
H 3.5583188044 -2.2849785208 -0.3471009201
H 2.2732405966 -3.2333767136 0.4141357022
H 2.3655660206 -1.958235688 -3.1421259816
H 1.2958906254 -0.5699374554 -2.8757314445
H 2.9469062471 -0.5645283077 -2.2241120893
H -0.427221404 0.9995316349 -0.0106496224
H -2.970051995 -4.312480747 -1.2182707596
H -1.6786541244 -3.3221419002 -1.9216468531
H -1.2781563427 -4.6646388672 -0.8277872516
C 1.5990210164 0.4127930914 -0.0606641787
C 0.8896159839 1.5499416275 -0.1599429005
C 3.033793046 0.42929872 0.374448834
C 1.4138053611 2.8732232867 0.1478344375
O 3.2871469975 -0.4782933288 1.3404218451
O 3.865157075 1.2268189512 -0.0231380844
C 4.6317537245 -0.4807128934 1.8605961473
H 4.6605989998 -1.2860703663 2.5946313979
H 5.3566603153 -0.6678900267 1.0646263073
H 4.8600384579 0.4747568265 2.3395122143
O 1.6281600351 3.6056298389 -0.9798705432
O 1.5326712497 3.3524811887 1.2698932581
C 2.0459146508 4.9631237032 -0.766768227
H 2.1607360772 5.3974273242 -1.7611129698
H 1.2966909591 5.5233070934 -0.2000430399
H 2.9992544442 5.0038179477 -0.2320123023

12c_t1
C -1.1022208954 0.2975663319 0.7228680268
N -1.5442746226 -0.6027126762 1.5257557622
N 0.1572831491 0.2815209811 0.0373613512
N -1.8237289715 1.4572259216 0.5059643474
C -3.0907035863 1.7243987142 1.1982380696
H -1.8167095737 1.7594444117 -0.4625397539
C -0.7580059836 -1.72255194 2.0295514231
C 0.7393098631 1.5697722032 -0.4405653477
C -4.2744154987 0.9731123281 0.5734836548
C -3.3281552048 3.236988913 1.2137319281
H -2.9568267619 1.3692845376 2.2238977742
H 0.2663834964 -1.7276494879 1.6388379513
C -0.6646509683 -1.6065229397 3.5588182035
C -1.4391334077 -3.0376367174 1.6256451722

H 0.3214511305 2.3523575634 0.1933045818\
H 0.4170332107 1.7617393933 -1.4707769551\
C 2.2259658422 1.6519535595 -0.4041673113\
H -0.1119549549 -2.4530036969 3.9822491718\
H -0.1502174186 -0.684354331 3.8500697106\
H -1.6657064329 -1.5909582886 4.0053220914\
H -0.8813397399 -3.9007387304 2.0059138619\
H -2.4560941219 -3.0807722248 2.0328041753\
H -1.5094380578 -3.1293295008 0.5368185226\
H -4.2521918402 3.4754086935 1.7504305695\
H -2.5002964352 3.7604122962 1.7027746169\
H -3.4240918737 3.6290728368 0.1936478144\
H -5.1957946494 1.1756384292 1.1310182408\
H -4.4307775359 1.2888810146 -0.4645561977\
H -4.0975050523 -0.1052461891 0.5853302946\
C 3.184103455 1.594665032 -1.375849436\
C 4.4451319192 1.7942144417 -0.7217420906\
C 4.1597924418 1.9609204589 0.5994407102\
O 2.8147698693 1.8858575382 0.8105296503\
H 4.7671021696 2.1466404054 1.473065035\
H 5.426194475 1.8143230046 -1.1754507472\
H 3.0089886667 1.4325167451 -2.4305266434\
C 0.5568693809 -0.8673679447 -0.6802203231\
C -0.2034381411 -1.5404239777 -1.5689618545\
C 1.9373625387 -1.4479586719 -0.4405782921\
C -1.5231010114 -1.1115343358 -2.0813100581\
O 2.4469317031 -1.1037235748 0.7481976452\
O 2.4944357278 -2.1889795501 -1.2295043214\
C 3.7440014138 -1.6572561899 1.0580686513\
H 4.0184655069 -1.2316659028 2.0224424915\
H 3.684216872 -2.7463804284 1.1260188692\
H 4.4728441261 -1.3774894122 0.2954703452\
O -2.1956090124 -2.1691200174 -2.5717842859\
O -1.9472111572 0.0322614296 -2.1210466301\
C -3.4810239748 -1.8835248652 -3.1623797986\
H -3.8668171777 -2.844939854 -3.5001018475\
H -4.1540149603 -1.4433980979 -2.422955012\
H -3.3706471359 -1.2003954241 -4.0080272902\
H 0.1825372 -2.4705952147 -1.9693375174

E-**12c_t1**

C 0.2479265528 -1.657630835 -0.6646462071\
N 0.1142239251 -2.1429554644 0.6108109568\
N -0.0096420399 -0.2463834861 -0.8083697372\
N 0.5899416591 -2.3042027214 -1.719880139\
C 0.7093367193 -3.7636046584 -1.698891078\
H 2.1512708063 -0.5824719144 0.8082245303\
H 0.3806683535 -3.1144486367 0.7144672817\
C -0.8116351594 -1.6698202382 1.6578607334\
C -0.7233224919 0.176507147 -2.0420802363

C	2.1567102783	-4.1771428612	-1.3882584609
C	0.2623406577	-4.3048203805	-3.0610972345
H	0.0535403599	-4.2067603226	-0.9306965808
H	-0.968368736	-0.6021869086	1.4956216123
C	-2.1648719042	-2.386877195	1.5671358277
C	-0.1547872889	-1.8718696692	3.0259390979
H	-0.7329938062	-0.7059445892	-2.6840813431
H	-0.1516666305	0.9481798118	-2.5593148431
C	-2.1080389118	0.6842523181	-1.8151079364
H	-2.8394694841	-2.029205062	2.3529266661
H	-2.6421243618	-2.203863174	0.6003533134
H	-2.0404269935	-3.4686771367	1.6948789778
H	-0.8177359839	-1.5185586547	3.8219521053
H	0.0489441021	-2.9343013247	3.2066737929
H	0.7896721087	-1.3241570957	3.0956499444
H	0.3275324247	-5.3979505525	-3.0871428088
H	-0.7735921602	-4.0196457048	-3.2731268159
H	0.8950289649	-3.9024700833	-3.8605588269
H	2.2623919382	-5.2670746173	-1.4174123635
H	2.8423646187	-3.7456993228	-2.1262874732
H	2.4677332354	-3.8334721952	-0.3958843633
C	-2.7383071165	1.8442643432	-2.1620111687
C	-4.1085183613	1.7056165439	-1.7569258581
C	-4.2119347135	0.4729739812	-1.1912138263
O	-3.0050440791	-0.1680410212	-1.2242553825
H	-5.0249699246	-0.073430903	-0.7367471263
H	-4.9030675774	2.430174327	-1.8685744783
H	-2.2801089281	2.6932163641	-2.6493155807
C	0.6877803909	0.6874516821	-0.0648827292
C	1.757070174	0.4211843039	0.7327959302
C	0.1768487275	2.1170761008	-0.183442112
C	2.4061694404	1.4461867081	1.5418953118
O	-0.7160084735	2.3738707407	0.7750515252
O	0.5071910473	2.882812384	-1.064302297
C	-1.2381034635	3.7210518485	0.8063356837
H	-1.9253675403	3.7463488799	1.6513465935
H	-0.4239887083	4.4334647619	0.9552164653
H	-1.7676210669	3.9491487057	-0.1207763503
O	3.4236001139	0.9078032393	2.2610650558
O	2.1204415763	2.6364866867	1.5960596123
C	4.1570281656	1.8270309947	3.0882577681
H	4.9264216435	1.2313611006	3.5803955632
H	4.6196799413	2.6121800024	2.4842101858
H	3.5041339801	2.2869387534	3.8349468227

12c_t2

C	-0.0769897144	-0.7800839145	-0.6500513549
N	1.1960486156	-0.1802056975	-0.6824465356
C	2.204389509	-0.652161361	-1.6755935176
N	-0.2789362244	-1.879924007	-1.2851142293

C	-1.6271250286	-2.3763615702	-1.5568885512
N	-1.0231953851	-0.0109053376	0.037861034
C	-1.7872925326	-0.5320282774	1.1788819245
C	-3.1123362969	0.1387006628	1.3242238349
H	-1.9219293118	-1.6069974442	1.0438853706
H	-1.2439594464	-0.393440536	2.1231139953
O	-4.0488401613	-0.0845844459	0.3471239543
C	-5.1687454375	0.6185445229	0.6904130799
C	-4.9714974829	1.2814488851	1.8619025218
C	-3.6316191035	0.9701019121	2.2733953429
H	-3.1194795651	1.3186702888	3.1597460086
H	-5.685951396	1.9139629243	2.3702305147
H	-6.0025430828	0.5435720057	0.0081462925
C	3.1991139537	-1.6618645129	-1.0897858004
H	1.5982478494	-1.1736455334	-2.4179281931
C	2.9189469621	0.5138207308	-2.3630927119
C	-1.8006607034	-2.5015409906	-3.0782707559
C	-1.8210986948	-3.7419877306	-0.8818438632
H	-2.4009154603	-1.6887818299	-1.188710174
H	-2.8169151648	-4.1463131738	-1.0953874072
H	-1.0755161008	-4.4571571258	-1.2488258832
H	-1.7080165145	-3.6733926399	0.2054607541
H	3.8460154267	-2.043327658	-1.8872135702
H	3.8472138647	-1.2048632064	-0.3329403827
H	2.6785147687	-2.5087979835	-0.6364949588
H	3.5691083514	0.1227475963	-3.15295181
H	2.202117913	1.1994023304	-2.8239465999
H	3.5508047517	1.0797958271	-1.6704799659
H	-0.7427451689	0.956665706	0.1528037788
H	-2.7917279153	-2.8974019428	-3.3283431469
H	-1.6914187972	-1.5265551946	-3.5654525584
H	-1.0450803008	-3.1759813285	-3.4980461981
C	1.6658846202	0.5203907278	0.4646340291
C	1.8706850293	1.8487194868	0.5056817744
C	2.0266963105	-0.2395920259	1.7249749727
C	1.4235385379	2.8131076981	-0.5322489316
O	1.566924206	-1.4971911692	1.705212707
O	2.6370226852	0.2449213748	2.6604327422
C	1.8428524654	-2.288014406	2.8822043439
H	1.3935662996	-3.2615055139	2.6898741975
H	2.9206769832	-2.3874774396	3.0286526087
H	1.3938486743	-1.8278711943	3.7652259257
O	2.3134503705	3.8069040546	-0.6727567273
O	0.3644186626	2.7535468297	-1.131710235
C	1.9634490597	4.8427387212	-1.6180934446
H	2.7909206634	5.5509148125	-1.5931653917
H	1.8517832156	4.4240805899	-2.6208561806
H	1.0335968668	5.3323312021	-1.3195880042
H	2.3534383738	2.269341301	1.3821473752

E-**12c_t2**

C	-0.6020822314	-0.6739952843	-0.056456987
N	0.7952607986	-0.6917573935	-0.4822581715
C	1.0406753601	-1.5984613757	-1.6527582515
N	-0.8256141643	-1.7132183545	0.8009067946
C	-2.0761536264	-2.3167315404	1.2889928269
N	-1.3299449766	0.2516541	-0.5620720196
C	-2.7216496707	0.5073403747	-0.1902945123
C	-3.6845272813	0.1136575381	-1.2658928903
H	-3.0219763599	0.0579153625	0.7633716693
H	-2.8293558329	1.5913842673	-0.0680969735
O	-3.8190171304	-1.2258455756	-1.5343318005
C	-4.7439952785	-1.3511189046	-2.5341215794
C	-5.2013690384	-0.126304727	-2.9082065219
C	-4.5101036912	0.8264889871	-2.0852318907
H	-4.6136042053	1.9027260397	-2.0995962242
H	-5.9359076675	0.0776511514	-3.6750254776
H	-4.9567004407	-2.358958028	-2.8584163978
C	2.3040202729	-2.4595173705	-1.5383825776
H	0.1974959029	-2.2913787704	-1.5994004135
C	0.9478777551	-0.846643524	-2.986221408
H	0.0184702612	-2.1855336561	1.1005556216
C	-1.9386925632	-3.8411107339	1.2097596434
C	-2.3998315371	-1.8565233743	2.7155929778
H	-2.8765242909	-2.0152291274	0.6122156397
H	-3.3392122438	-2.3036689204	3.0577732688
H	-1.6053117379	-2.1619610909	3.4061195393
H	-2.4978982448	-0.7680702576	2.7735261585
H	2.2232294157	-3.275039499	-2.2641322965
H	3.2190706619	-1.9130593734	-1.7746664272
H	2.3981154188	-2.9005624201	-0.5422556909
H	0.9922595155	-1.5622402237	-3.8142244626
H	0.0028220576	-0.3011134534	-3.053654678
H	1.7708014989	-0.136456339	-3.0968742693
H	0.2609832046	1.2959528625	1.2652959146
H	-2.8536201489	-4.3230149941	1.567855405
H	-1.75700533	-4.1666700917	0.1808184162
H	-1.1075999368	-4.190024986	1.834616297
C	1.6349274782	0.2773943603	-0.0052457739
C	1.2802248422	1.2305377062	0.9116441395
C	3.0724248552	0.2668461539	-0.5073275955
C	2.2160296449	2.2098136678	1.4326446191
O	3.8619801494	-0.4016450664	0.3370013224
O	3.4302845055	0.7719447804	-1.5506491885
C	5.2697368909	-0.3974565048	0.0145753166
H	5.7514207732	-0.9708278867	0.8060383728
H	5.4468088631	-0.8676201463	-0.9555763067
H	5.6455580417	0.6276594415	0.0029125705
O	1.6079833519	3.0073712814	2.3539502344
O	3.3943520479	2.3584039963	1.1220336847

C	2.4332393171	4.0313593035	2.9300568166
H	1.7921596974	4.5684689312	3.6302116937
H	3.2850143844	3.5974278936	3.4613124304
H	2.803751662	4.7162207946	2.1621254127

TS4$_{5C}$(1c_t1)

C	-1.3018402781	0.6308783501	-0.2796831367
N	-1.6365530543	1.8960511081	-0.6307259737
N	-0.0015996548	0.1314085556	-0.4261322678
N	-2.1424780234	-0.281195645	0.1376380674
C	-3.5627696478	-0.041100521	0.4414093772
H	-2.6390537681	2.0160730524	-0.7270621171
C	-0.9743329201	3.1815435365	-0.2713458349
C	1.0913509118	0.890293354	-1.035855714
C	-3.982674513	-0.7098233023	1.7555324452
C	-4.4612137596	-0.4588126605	-0.7308403465
H	-3.6768958971	1.0397380182	0.586941191
H	0.0480279316	2.95954232	0.0307296239
C	-0.9647931078	4.0974173389	-1.4953235703
C	-1.6982958289	3.8226637011	0.9172157523
H	1.6574735208	0.2049718467	-1.6678200013
H	0.6627575545	1.6596181892	-1.6818878863
C	2.0174981616	1.4915178343	-0.0198130379
H	-0.4837205999	5.049066495	-1.2494145127
H	-0.4232842025	3.6419058741	-2.3299271616
H	-1.9877625252	4.310714269	-1.8267111374
H	-1.2163184265	4.770047714	1.1796392912
H	-2.7451110025	4.033216068	0.6690100409
H	-1.6740121561	3.1726995634	1.7970828092
H	-5.5007327785	-0.196342916	-0.5082948927
H	-4.1717184872	0.0535639092	-1.6538501022
H	-4.4069221098	-1.5360253863	-0.9056224124
H	-5.0188229782	-0.4301525405	1.9726228286
H	-3.9076203722	-1.7958204816	1.6965894067
H	-3.3535251567	-0.3741172732	2.5844698877
C	2.1180009798	1.4396944968	1.3394949588
C	3.2747693263	2.2191548261	1.6879514843
C	3.7876745903	2.684592998	0.5187110499
O	3.0297881242	2.2499740243	-0.5360261257
H	4.6344333194	3.305364962	0.2670301546
H	3.6643202242	2.4030013567	2.6795788551
H	1.4603212588	0.9084803719	2.0124057745
C	0.0601042217	-1.2065268998	-0.0823194434
C	1.0799220664	-2.0940943726	0.0158681992
C	-1.3672078366	-1.7473046578	0.2594696386
C	2.5087859494	-2.0316748197	-0.2877488953
O	-1.8082073898	-2.4040673696	-0.962121702
O	-1.5270336429	-2.3486121979	1.3480824024
C	-2.4469220251	-3.657671873	-0.7649595899
H	-2.5175770839	-4.1199316831	-1.7545014319

H -1.8760399972 -4.3128502802 -0.0990528781
H -3.461660665 -3.5603390155 -0.3553481385
O 3.1373003214 -3.0786812489 0.3113267782
O 3.1212908643 -1.2366212895 -0.9900472848
C 4.5474311674 -3.1882310604 0.0578466581
H 4.8742598778 -4.0799522676 0.5938288397
H 4.7454845233 -3.2996086857 -1.0117227462
H 5.0815967512 -2.3101664179 0.4308438067
H 0.7607662424 -3.0492172676 0.42058602

TS4$_{5C}$(1c_t2)
C -0.5528061129 0.7604966712 0.1765829238
N -0.8408097569 -0.3691016437 0.7862738013
N -1.3733972738 1.7283196314 -0.242594234
N 0.8261803348 0.8821493008 -0.0539805734
C 1.5445082713 2.1805912458 -0.2636458555
C 0.501907908 -1.1616410139 1.1586078358
C 1.4692324864 -0.2936213268 0.3125822249
C -2.8072896951 1.9423501168 0.0602921835
C 2.7101372689 -0.773416673 0.0598506587
C -2.0943026351 -1.0762959675 1.0110075487
C -2.6584481214 -1.7202256185 -0.2211636554
H -1.8304700958 -1.8585683947 1.7289151692
H -2.8464432759 -0.4384995134 1.4785852918
H 2.9028089879 -1.7376465864 0.518499577
C 3.7679243043 -0.2899040213 -0.8210499597
O 0.4358116121 -2.4055274076 0.958314037
O 4.8580420386 -1.0953577523 -0.7132135305
O 3.7421948559 0.6594072378 -1.5964532761
C 5.9638569453 -0.7663636168 -1.5698247491
H 6.7360606942 -1.5045431169 -1.3507111705
H 5.6751204422 -0.8275064183 -2.6226961783
H 6.3360918371 0.2398560097 -1.3588893592
O -3.9520605706 -1.4065581455 -0.5526027387
C -4.2617675564 -2.1128124945 -1.6821725274
C -3.1960588145 -2.8630889575 -2.0747754544
C -2.1531292554 -2.6103620267 -1.1225214841
H -1.1533725908 -3.0185892981 -1.0902654716
H -3.1570775887 -3.5192419464 -2.933406465
H -5.2611116422 -1.9742654009 -2.0670536036
C -3.3644165554 2.8872494981 -1.0047521725
H -3.3219404535 0.9854720742 -0.0374453368
C -3.0037955523 2.4905114363 1.4767184299
H 2.5894800817 1.8921351728 -0.2082142425
C 1.3441429694 2.7753690969 -1.6619203529
C 1.2744482 3.170585116 0.8753875225
H -4.0728562913 2.6041853209 1.6837090752
H -2.581749962 1.817199318 2.2288435203
H -2.5266312722 3.4705432786 1.583361951
H -4.4305682939 3.0530215664 -0.828379574

H	-2.8598395605	3.8598672027	-0.9677330626
H	-3.2419400855	2.4679328269	-2.0080208332
H	1.9534666825	4.0221980012	0.7666670692
H	0.2527541623	3.5592179255	0.8731172381
H	1.46698416	2.703980169	1.8461813038
H	2.094997311	3.5565670356	-1.8167157165
H	1.4874906228	2.0111530164	-2.4283284591
H	0.3674294384	3.2486915809	-1.811353571
O	0.7343937853	-0.6992978121	2.5435124411
C	0.8988854139	-1.7158656364	3.5186272738
H	1.8125275842	-1.5197980647	4.0952850146
H	0.0489413957	-1.7269491104	4.2169378283
H	0.9720505089	-2.69843383	3.0432661088
H	-0.9387012909	2.4794419441	-0.7602244208

13c_t1

C	-0.8662092743	-1.4382509723	-0.2922970674
N	-0.320061543	-0.1604876602	-0.41572658
N	-2.1114654366	-1.7616756511	-0.6814974818
N	0.0083248182	-2.2970742179	0.1954181245
C	-0.2619387082	-3.6624702587	0.6858914888
C	0.9799233974	-0.15108058	0.0784268802
C	1.4149166616	-1.626234473	0.333590325
C	-3.3934578882	-1.0171544671	-0.5563816559
O	2.0955763308	-1.9085431953	1.3575457985
C	-0.9516990433	0.9358626685	-1.1533316366
C	-1.6446134402	1.9205778948	-0.2581841077
H	-0.1672480895	1.4528801222	-1.7077787263
H	-1.6458645412	0.5110822121	-1.8826298692
C	1.8708948985	0.832750309	0.341976108
O	-2.2620190607	2.9529637284	-0.9051254109
C	-2.808348621	3.762728642	0.0548099738
C	-2.5513015331	3.2625052242	1.2919549019
C	-1.7917143513	2.0584723867	1.0907895854
H	-1.402634463	1.3958377208	1.8504155823
H	-2.8605234713	3.6901404183	2.2355544002
H	-3.3383556943	4.6312765099	-0.3063308779
C	-4.2235144206	-1.6287666593	0.5766493585
H	-3.1586998059	0.0120941092	-0.2913880223
C	-4.1340272417	-1.0557469786	-1.8935380129
H	-1.2387240544	-3.9649393346	0.2970375756
C	-0.3376743849	-3.6755245264	2.2196108834
C	0.7771123704	-4.651559688	0.1491008386
H	-5.0827046986	-0.5165864919	-1.8112861188
H	-3.5431448475	-0.5954480619	-2.6910550697
H	-4.3553333227	-2.0893808243	-2.1836362011
H	-5.1747738728	-1.0952066197	0.6693450243
H	-4.4448026552	-2.6830000812	0.373491774
H	-3.6988579868	-1.5620063272	1.5344025103
H	0.5159590067	-5.6601012717	0.4854489518

H 0.8027109155 -4.6442066403 -0.9433984828
H 1.7720155064 -4.4002412598 0.5211248261
H -0.5500641527 -4.6912700398 2.5696705817
H 0.6100767853 -3.3359493991 2.6404450879
H -1.1366432719 -3.0192813859 2.579441946
C 1.8749987113 2.2714696789 0.0759003836
H 2.7466425011 0.4662222588 0.868751821
O 2.8054750087 2.8730267734 0.8626029662
O 1.213365956 2.9055052416 -0.7359760699
C 2.9597372671 4.2897089583 0.6746869186
H 3.7471114176 4.5942223632 1.3649679087
H 3.2525320043 4.5180280635 -0.3537060824
H 2.031517516 4.818207524 0.9082314619
O 1.9858214973 -2.0321236394 -0.9930583192
C 3.3865755923 -1.8563232535 -1.0859899149
H 3.729470936 -2.4411662188 -1.9465295938
H 3.6697175679 -0.8047588224 -1.2542422448
H 3.8957089698 -2.2125632623 -0.1834810452
H -2.2621787613 -2.7635645461 -0.7310573954

13c_t2
C -0.2444553759 -1.0107898668 -0.2968741365
N -0.8331792204 0.1357727852 -0.5771196539
N -0.7894387634 -2.2208948628 -0.1542867258
N 1.1339581743 -0.8475203214 -0.1135396026
C 2.1521129732 -1.9342367948 -0.2861038552
C 0.2392827868 1.2825292089 -0.7280863929
C 1.4458677964 0.5090881531 -0.1396983399
C -2.144912442 -2.6903256645 -0.5257180601
C 2.5426800285 1.2034699541 0.2468425466
C -2.2290516777 0.5586148717 -0.5804852531
C -2.8313755203 0.6822901682 0.7871541921
H -2.1928980066 1.5500843817 -1.0406917428
H -2.8481338559 -0.0810765485 -1.2115498609
H 2.4480001735 2.2742994054 0.1034233559
C 3.7548830599 0.7803183455 0.9423587004
O -0.0930436052 2.4001779148 -0.2433700077
O 4.5902288709 1.8420539436 1.0919954851
O 4.0336232124 -0.3272722121 1.3872945132
C 5.8079467479 1.5818910288 1.8094455428
H 6.3457598283 2.5302975218 1.8305195054
H 5.5974922909 1.24776475 2.8290868996
H 6.4068537089 0.8219980604 1.3001953894
O -3.9586209894 -0.0552046315 1.0471869023
C -4.3358391346 0.2234749995 2.3317599567
C -3.4741916118 1.1159878978 2.8921462906
C -2.4968929907 1.4175424604 1.8862107643
H -1.6475825356 2.0826844694 1.9467499111
H -3.5294111209 1.5152957798 3.8955448555
H -5.2224550323 -0.2822555502 2.6840671083

C -2.3979463032 -3.9989641964 0.2229781474
H -2.8682476339 -1.9549161671 -0.1707520689
C -2.2817872289 -2.8558234889 -2.0419593677
H 3.0916045361 -1.3903604409 -0.2702775602
C 2.2086349549 -2.9135513078 0.8911023241
C 2.0317678314 -2.6188475019 -1.6526305922
H -3.3026187692 -3.1654715648 -2.2881286909
H -2.0752029387 -1.9184106453 -2.5671664137
H -1.5910760394 -3.6201535551 -2.4135919216
H -3.401240223 -4.3665932269 -0.0081074051
H -1.6772724544 -4.7681593684 -0.0783476001
H -2.3240881676 -3.8556300054 1.3053068862
H 2.9067178273 -3.2598613816 -1.7998892959
H 1.1421621422 -3.2479924314 -1.7406211163
H 2.0146674341 -1.8767609081 -2.4564811814
H 3.1316351374 -3.4967320375 0.813137818
H 2.227612909 -2.3741573307 1.8401999541
H 1.3851450179 -3.6361709181 0.9093835087
O 0.424538498 1.2232448576 -2.2041735181
C 1.0809844408 2.3601035912 -2.7374523688
H 0.9357066883 2.3357050823 -3.8226969634
H 0.6594269223 3.2890834804 -2.3382782112
H 2.1642478177 2.352822992 -2.5374045397
H -0.163365168 -2.9480281751 0.162739889

15c_t1

C -1.2759897955 0.700695065 -0.1468875232
N 0.0170172809 0.1447097166 -0.4156629393
N -1.7282609268 1.888202442 -0.2003186492
N -2.0780028816 -0.3651463839 0.246468569
C -3.4599441711 -0.1695255048 0.7250273483
C 0.0481743547 -1.2010048708 -0.1377549358
C -1.4131625516 -1.6253000197 0.0804345614
C -1.0624550825 3.1548336606 -0.4833154819
O -1.4973157463 -2.4881688292 1.1753732136
C 1.1461398655 0.9179548016 -0.9222663112
C 1.9369635997 1.586724855 0.1632438869
H 1.796019938 0.2298368701 -1.4643367726
H 0.7778723421 1.6546638024 -1.6380344928
C 1.0311347279 -2.1364012512 -0.0390087758
O 2.8303193836 2.529006751 -0.2672500752
C 3.4892359481 2.9974219743 0.8383923468
C 3.0313435338 2.3738705003 1.95574538
C 2.0187315404 1.4521213636 1.5178863295
H 1.4384160803 0.7789102573 2.1324480366
H 3.3652524396 2.544833565 2.9697762462
H 4.2329027171 3.7587948009 0.6567867659
C -1.6017692571 4.1969080907 0.5064184349
H 0.0220907951 3.1108438677 -0.3408478788
C -1.349503234 3.5809526347 -1.9304616567

H -3.68346909 0.8593361539 0.4358019203
C -3.5520661983 -0.2577759381 2.2563191024
C -4.4810746518 -1.0819078504 0.0358931069
H -0.9142651114 4.5649838567 -2.1360085041
H -0.9327280185 2.8667695338 -2.6486214528
H -2.4303375786 3.6395969817 -2.1025281758
H -1.1396866072 5.1743210847 0.329406395
H -2.6876663119 4.3025453932 0.4020457512
H -1.3870058559 3.8992147817 1.5381203772
H -5.4864858139 -0.7779974539 0.3457414257
H -4.4148676863 -1.0047225753 -1.0517763717
H -4.359641115 -2.1349153256 0.310012778
H -4.559736803 0.0214529252 2.582773729
H -3.3466144258 -1.2683356477 2.6206771949
H -2.8416212197 0.426124025 2.7308080947
C 2.4743993593 -2.0907618867 -0.2717404522
H 0.6846163171 -3.0977174563 0.3187340472
O 3.0439970016 -3.1972720889 0.2743377673
O 3.1417597673 -1.2583662171 -0.8737549255
C 4.4645728282 -3.3226709321 0.0937766841
H 4.7396569051 -4.2624923518 0.5734303596
H 4.7222863556 -3.3532977643 -0.9682735431
H 4.9933584277 -2.4912276018 0.5673931208
O -1.9621193271 -2.2269032423 -1.0891500207
C -1.4867656211 -3.5190479196 -1.4814224697
H -2.1655691596 -3.8542597427 -2.2688797108
H -0.4690498063 -3.4742652058 -1.8837270219
H -1.5168860556 -4.2380175754 -0.6563456247
H -2.3793184052 -2.8965051194 1.1626697923

E-**15c_t1**
C -1.2447742231 -1.143954601 -0.1026871609
N -0.5313270979 0.0462643294 -0.4570991525
N -2.4874296361 -1.4179289063 -0.0751980999
N -0.2788357953 -2.0562352979 0.2932632834
C -0.6221986928 -3.378605979 0.8505680512
C 0.813201721 -0.0838892711 -0.2038899831
C 1.0537882312 -1.5883285244 0.0339002771
C -3.6288415665 -0.5342801728 -0.2924996536
O 1.9115649337 -1.9073668636 1.0607521298
C -1.0927182916 1.209052652 -1.1345389117
C -1.4797056027 2.3217979918 -0.2038846709
H -0.3462258121 1.5668702611 -1.8532920417
H -1.9500939682 0.8884459256 -1.7287368211
C 1.7051977025 0.9462942365 -0.2497475293
O -2.0324363785 3.4158549551 -0.8105031601
C -2.3079579368 4.3269496119 0.1739465821
C -1.9416657178 3.8334753235 1.3859965809
C -1.4002090869 2.5242344528 1.142281186
H -1.003023333 1.8347373196 1.8732220495

H	-2.041138205	4.331986453	2.3401151349
H	-2.7549519181	5.2537807225	-0.153137791
C	-4.6481023428	-0.8046048999	0.8234766113
H	-3.3621056705	0.5269782864	-0.2302575595
C	-4.2485769602	-0.8096601081	-1.6698744941
H	-1.6663060144	-3.5192473923	0.5616470857
C	-0.5495796237	-3.3796596329	2.3845879558
C	0.2002982233	-4.5161335784	0.2369692483
H	-5.1450242998	-0.1982188767	-1.8196274654
H	-3.5450915725	-0.5877381427	-2.4794361159
H	-4.5337079705	-1.8646509013	-1.7533288937
H	-5.5334149342	-0.1709412564	0.7010629821
H	-4.9671646223	-1.8529755778	0.8081716459
H	-4.2121990539	-0.5967016209	1.8062653206
H	-0.1676351312	-5.4691737292	0.6316450512
H	0.1062628529	-4.5331237676	-0.8515356724
H	1.2604029923	-4.4302286212	0.4883465161
H	-0.9119496455	-4.3374082069	2.7745398639
H	0.475906001	-3.2290293048	2.7309884746
H	-1.1766507936	-2.5871510737	2.8055469819
C	3.1034776541	0.8859321924	0.132847311
H	1.3495350999	1.9350360491	-0.5133726762
O	3.7223758953	2.0542843047	-0.1110629822
O	3.710045863	-0.0630035391	0.6508849595
C	5.1084404211	2.139980153	0.2760674197
H	5.4212232175	3.1494392634	0.0096199084
H	5.2201124582	1.9807202024	1.3512353188
H	5.7071216235	1.4036024364	-0.2652531037
O	1.4405150192	-2.1738289675	-1.2213419599
C	2.7876278779	-2.0166148583	-1.6608665352
H	2.8908950781	-2.6615822345	-2.5370402365
H	3.0066983505	-0.9846905459	-1.9622042409
H	3.5081754843	-2.333592857	-0.9008663424
H	2.6864311966	-1.2871858132	1.0045493244

15c_t2

C	-0.5409527215	0.8739615281	0.1957026329
N	-0.8340560801	-0.4443759047	0.5505701288
N	-1.2568848326	1.8896447939	-0.0787359678
N	0.8813859548	0.9621888196	0.1187308665
C	1.6109313662	2.2427314876	-0.0824622266
C	0.3777314188	-1.1727993624	0.8712230006
C	1.4665690639	-0.2608193747	0.2957931489
C	-2.7158424507	1.9314217541	-0.1702385209
C	2.7138874379	-0.7718581835	0.0845638554
C	-2.0999295486	-0.9303078305	1.0892391943
C	-2.9349127249	-1.6346106508	0.0594329648
H	-1.8643099774	-1.6133659755	1.9134579621
H	-2.6644648367	-0.113546551	1.5438905982
H	2.8236614733	-1.8105469876	0.367477334

C	3.8947276559	-0.2208544298	-0.5700319142
O	0.4540758845	-2.4324694671	0.2810283316
O	4.9142297574	-1.1193462186	-0.4802113238
O	4.0306583629	0.843761739	-1.1624718463
C	6.1385758369	-0.7399006338	-1.1283821134
H	6.8325403083	-1.5623577979	-0.9510903698
H	5.9874171944	-0.6027783447	-2.2026946862
H	6.5371582685	0.1850389966	-0.7028490788
O	-4.2175494306	-1.9245531364	0.4374222229
C	-4.8027540222	-2.5970842906	-0.6023243808
C	-3.9200886	-2.7378201687	-1.6263337901
C	-2.6996423159	-2.1114913323	-1.1964285873
H	-1.7716687127	-2.0325280293	-1.7433079178
H	-4.1077992126	-3.2230245881	-2.574146401
H	-5.8307131225	-2.8925099408	-0.4542921596
C	-3.0828583529	2.6877019074	-1.4547209737
H	-3.161467934	0.9330068254	-0.2483122158
C	-3.3007260921	2.6387138003	1.0602857608
H	2.6565786492	1.9605074575	0.0034334673
C	1.418032394	2.8160675659	-1.4890761178
C	1.3069413357	3.2514372433	1.0313282255
H	-4.3899660613	2.719055446	0.9759046916
H	-3.0690079672	2.0975780753	1.983669256
H	-2.8887113796	3.6503018995	1.1508101495
H	-4.1708092281	2.7470370207	-1.5693338959
H	-2.6821596467	3.7074936607	-1.430986757
H	-2.6743546845	2.1810636915	-2.3352765253
H	1.998484735	4.0955345936	0.9375039773
H	0.2861671111	3.6313955464	0.9739727703
H	1.4599423435	2.8007591615	2.0176404327
H	2.0782877883	3.6808033499	-1.6160668012
H	1.6899783827	2.0736258259	-2.2444810631
H	0.3888923997	3.1372740279	-1.6575355602
O	0.4187468415	-1.2561955348	2.2868531068
C	1.4857880127	-1.9989252438	2.8888615265
H	1.2274068523	-2.078396617	3.9468428943
H	1.5736006615	-3.003413066	2.4642067809
H	2.4440231558	-1.477526927	2.7929053452
H	-0.2100937114	-3.00423463	0.7040465692

E-**15c_t2**

C	0.7322756369	1.052762455	-0.2196215598
N	0.5983493388	-0.3232860048	-0.3736484397
N	1.7299945623	1.8412484074	-0.157265599
N	-0.5900031351	1.5691435997	-0.063207234
C	-0.8900011476	2.9983829632	0.2123187722
C	-0.7984109066	-0.7221966586	-0.4536155813
C	-1.521249022	0.5749458047	-0.0368257008
C	3.1414459756	1.4577467811	-0.1590313002
C	-2.8497288225	0.713706135	0.2626727357

C 1.5945439427 -1.2113529875 -0.9590401778
C 2.3635355362 -1.9930286849 0.0660419912
H 1.0688534766 -1.8949506576 -1.633668599
H 2.2825690006 -0.6466942736 -1.5931330112
H -3.2509237753 1.6959167449 0.4742980929
C -3.814039824 -0.3573674587 0.3588843023
O -1.0182206293 -1.8049262729 0.3644250146
O -5.060977482 0.1271873184 0.5264479344
O -3.6119706081 -1.5829882043 0.3293617785
C -6.1107058012 -0.8459991851 0.6843538027
H -7.0248550612 -0.267430014 0.8179662628
H -6.1913783367 -1.4778283134 -0.2036090306
H -5.9302134963 -1.4734819527 1.560664336
O 3.4510841 -2.6732888404 -0.4122810405
C 4.0049129545 -3.3450275394 0.6448652396
C 3.2948494145 -3.1024108675 1.7783613288
C 2.2226181746 -2.2218130744 1.4025573457
H 1.4438941916 -1.8229615445 2.0352927611
H 3.5028701525 -3.4971982944 2.7632736512
H 4.8835016013 -3.9322291681 0.4229208134
C 3.8331975014 2.203999247 0.9905042379
H 3.2872410726 0.3864017975 0.0192516676
C 3.7793891311 1.8250401181 -1.5059136855
H -1.9782954649 3.0511411909 0.2077033989
C -0.4133209301 3.4310973545 1.6027121054
C -0.4101864521 3.9214352765 -0.91314369
H 4.8497566262 1.5920976346 -1.5007964671
H 3.3148382674 1.277202081 -2.3324460884
H 3.6622481555 2.8965663992 -1.7045374507
H 4.9002685534 1.9577595634 1.0220470465
H 3.7325851156 3.2879751524 0.8639939815
H 3.3921755541 1.9301756041 1.9546254248
H -0.8220038825 4.921913784 -0.7445699207
H 0.6770990652 3.9889608761 -0.9452942158
H -0.7708910024 3.5680744855 -1.8845789127
H -0.7842345035 4.4398424645 1.8116500174
H -0.8041019877 2.7604984835 2.3747097525
H 0.6762565733 3.4424297789 1.6655306728
O -1.1337110353 -0.9621159637 -1.8225205138
C -1.6370964943 -2.2500369891 -2.2005495213
H -1.696103168 -2.2212430133 -3.2913152741
H -0.9705074256 -3.0643684552 -1.9016997857
H -2.6357947173 -2.4346738717 -1.7944148726
H -2.0073285628 -1.9038012112 0.4238402034

16c_t1

C -1.3107913802 0.468825768 0.1081258913
N 0.0611024817 0.7880197801 0.2943179345
N -2.2352417928 1.3314964508 -0.1329181739
N -1.6373936874 -0.8842202346 0.2545858987

C	-3.0722774355	-1.2748963875	0.1906866167
C	0.8699403918	-0.0839507531	1.0393654631
C	-0.5664638659	-1.8837464403	0.2417833483
C	-2.1058465573	2.7671666647	0.1045110687
O	-0.9720356575	-3.1054336767	0.793347646
C	0.7372683206	1.7056418761	-0.6640473774
H	-0.0292213083	2.257430859	-1.1981646358
H	1.3349430375	2.41771213	-0.0911992903
C	1.5871725408	1.0189922483	-1.6818062015
C	0.567667663	-1.3875688614	1.0985467823
C	-3.0917222695	3.160192279	1.2175561919
H	-1.1018990382	3.0505152361	0.4505850679
C	-2.425572736	3.5421288076	-1.1817856336
H	-3.5687237574	-0.3170262249	0.0429128793
C	-3.5986458659	-1.8562488677	1.5107075116
C	-3.4237136873	-2.1364796121	-1.0313021049
H	-2.3758696007	4.6226781318	-1.0087582813
H	-1.7303159376	3.296457751	-1.9917849799
H	-3.4362155455	3.2965826112	-1.5279374517
H	-3.046617842	4.2376761855	1.4137521748
H	-4.1184257399	2.9049576498	0.9310515646
H	-2.8594416468	2.6349295074	2.1499934226
H	-4.5139553688	-2.1866247624	-1.1258027463
H	-3.0216764668	-1.6989735688	-1.949029944
H	-3.0688263846	-3.1712412128	-0.9663036955
H	-4.6909382101	-1.9291664327	1.460925688
H	-3.1990889009	-2.8487772557	1.7263530378
H	-3.3443510749	-1.1972296609	2.3472994409
H	1.0793301546	-2.0803610197	1.7524706763
C	1.9767707007	0.5283750241	1.8404387527
O	2.8386320823	-0.3980354311	2.2876875541
O	2.0797605271	1.7196102267	2.0710847499
C	3.9063981363	0.0904114792	3.1267808938
H	4.4919433403	-0.7889967653	3.3932500234
H	4.5241018977	0.8067566942	2.5802053786
H	3.503732076	0.5648159534	4.0248867232
O	-0.2022390427	-2.056290194	-1.1243149437
C	0.9282279246	-2.9017829645	-1.3659046716
H	1.0185025366	-2.9836715718	-2.4507808888
H	1.8516469604	-2.4707921981	-0.9634597035
H	0.784988547	-3.9020603141	-0.941899635
C	1.4193572445	0.7412641737	-3.0073968637
C	2.6119577626	0.0737500821	-3.4440116659
C	3.4209663513	-0.008031067	-2.352455968
O	2.8168016215	0.5672674263	-1.2703599383
H	4.4074264285	-0.4140439773	-2.1841785499
H	2.8354265748	-0.2903666553	-4.4371904912
H	0.5490252605	0.9848416423	-3.6009971615
H	-1.5177277625	-3.5608725284	0.132536616

16c_t2

C	-0.0251115719	0.9723359626	0.1099441485
N	0.6936743776	-0.2538740929	0.1044819022
N	0.5026615191	2.1411497019	0.0850261643
N	-1.4330550019	0.8716202289	0.1332241208
C	-2.1977108485	2.0868111965	0.5578592145
C	0.0240980313	-1.3577725802	0.8144443513
C	-2.0366164461	-0.3832316499	-0.0270653098
C	1.9368376011	2.3548327501	0.2637153031
C	-3.3493803599	-0.4812929485	-0.7469955879
C	1.4402146783	-0.578993	-1.1319044782
C	2.9147999828	-0.7932953315	-0.9419254412
H	0.9901418621	-1.463831975	-1.5958284942
H	1.3131365175	0.2440205688	-1.8373969964
C	-1.3762545927	-1.5127653493	0.285288919
O	3.6966174315	-0.338943092	-1.9741526323
C	4.9947810421	-0.6402824981	-1.6682514835
C	5.0579635235	-1.2696232554	-0.4642665543
C	3.7052874572	-1.374798136	0.0066560241
H	3.3605627751	-1.8298617963	0.9225350256
H	5.953182961	-1.6194463727	0.0313057086
H	5.7340999339	-0.3413353115	-2.396230659
C	2.1361938049	3.2758672679	1.4767608274
H	2.4653444249	1.4125462242	0.4609750541
C	2.5284124826	2.9923948914	-1.0009888157
H	-1.4864272981	2.6222775798	1.1905490935
C	-3.41142252	1.7378063444	1.4240762755
C	-2.5503582097	3.0163985463	-0.608188781
H	3.5937103445	3.2076335259	-0.8623669442
H	2.4271975316	2.3332244123	-1.8683133991
H	2.0158571603	3.9341756046	-1.2292574921
H	3.2005307119	3.4815859032	1.6368909766
H	1.6197909601	4.2309014413	1.3258152931
H	1.7376296873	2.815489758	2.387268075
H	-2.9898989835	3.9403759167	-0.2153786257
H	-1.6493217848	3.2829520555	-1.166438339
H	-3.2638927826	2.5495668393	-1.2889823236
H	-3.7829444831	2.6586281265	1.8848072382
H	-4.2399758212	1.3108484254	0.8521882735
H	-3.1434730349	1.0454371323	2.2282079023
H	-1.7862523433	-2.4871607849	0.0598748654
O	-0.0672946871	-1.028339847	2.1779734134
O	-4.0545026769	-1.5468665863	-0.3330048092
O	-3.7173524146	0.271362936	-1.629268571
C	-5.2797054476	-1.8059173216	-1.0522297689
H	-5.7038919166	-2.6973247907	-0.5911151749
H	-5.9669417156	-0.9627270141	-0.9513694933
H	-5.0740772323	-1.9859999383	-2.1100399159
O	0.8309587646	-2.4853230405	0.5959300027
C	0.4376742169	-3.6742670659	1.2881930859

H	1.2265257025	-4.4041877524	1.0979558556
H	0.3545152221	-3.4987471853	2.3645951394
H	-0.511543413	-4.0730533761	0.9120238796
H	0.8324308778	-0.7849942473	2.4605109572

17c_t1

C	-0.4776797355	-0.8282776364	0.5950110681
N	0.9208528486	-0.6399376003	0.4454805033
N	-1.2160889074	-1.8348231748	0.7977239322
N	-1.0542544083	0.46289647	0.4659019065
C	-2.5125821195	0.7103854994	0.5515941523
C	1.2133362465	0.6880021843	0.1992551523
C	-0.1120523703	1.404132805	0.2063853113
C	-0.8769066575	-3.254117479	0.8756172008
O	-0.3108200517	2.6047709675	0.018745873
C	1.9090559174	-1.693746691	0.6702958215
C	2.2224914131	-2.4706134453	-0.5735981098
H	2.8182376612	-1.2142455976	1.0342902553
H	1.5476582855	-2.3542393603	1.4604057489
C	2.3387232535	1.4130849964	-0.0316536113
O	2.9682567108	-3.5998454699	-0.3769943675
C	3.1973598302	-4.1524098887	-1.6090432732
C	2.6115157285	-3.3993582866	-2.5767989581
C	1.9751161625	-2.2999913167	-1.9037124884
H	1.4151090321	-1.493726619	-2.3553433306
H	2.6241480503	-3.5954433051	-3.6399367361
H	3.7778081982	-5.0627274427	-1.616958482
C	-1.9278695343	-4.0235966783	0.0653728098
H	0.1015206781	-3.4773697009	0.4395408699
C	-0.8808311868	-3.6970485256	2.3448792799
H	-2.9292589371	-0.2745913348	0.7669112532
C	-3.0629699039	1.2004447648	-0.7906933751
C	-2.834633151	1.6519966967	1.7151991298
H	-0.6967974995	-4.7741164263	2.414966503
H	-0.1074130367	-3.1793253666	2.9214659593
H	-1.8508187061	-3.4812834229	2.8062766141
H	-1.7207547317	-5.0984385254	0.0982607487
H	-2.9311630603	-3.8497497206	0.4696745165
H	-1.9209258565	-3.7058918042	-0.9822552041
H	-3.920711979	1.7429018068	1.8152736409
H	-2.4375121885	1.2591615671	2.6564337402
H	-2.419792439	2.6492815939	1.5489287566
H	-4.1515649976	1.2912787875	-0.7229807727
H	-2.6521994011	2.1773163202	-1.0579764818
H	-2.8297678323	0.4906922187	-1.5905761081
C	3.7546738209	1.0400629935	-0.1145880199
H	2.15022941	2.4648347575	-0.2202849835
O	4.4572516241	2.0890084185	-0.6033187125
O	4.2953053256	-0.0118796579	0.1997626295
C	5.8769727657	1.8890495388	-0.7291602137

H	6.2716670044	2.8295106397	-1.1139005007
H	6.3239612363	1.6606791526	0.2417693459
H	6.0936653306	1.0758458968	-1.4266960979
H	0.7469525035	4.067223851	-0.3893890174
O	1.3776323127	4.8053212447	-0.4741997097
C	1.4890475983	5.3954653689	0.8128690331
H	2.2333495701	6.1961085789	0.7511936296
H	0.5425907085	5.8410873865	1.1553229012
H	1.8249074642	4.6813959715	1.5794552673

E-**17c_t1**

C	-0.6369392915	-0.5960663838	0.3770257556
N	0.7410374534	-0.3670794237	0.1562753677
N	-1.3086132475	-1.5983052824	0.7643444887
N	-1.2777085722	0.6188308867	0.0390875878
C	-2.7428639394	0.8139043389	0.1169060505
C	0.9644608498	0.9220853819	-0.3082820058
C	-0.3964448754	1.5521076776	-0.4376447427
C	-0.8378798392	-2.9599095288	1.0156739444
O	-0.6854423861	2.6450692383	-0.906125531
C	1.8263110114	-1.1988380201	0.6654458668
C	2.444397867	-2.0668783336	-0.3906727502
H	2.5843856143	-0.5316719197	1.0925543565
H	1.4536721023	-1.8016750405	1.4950029238
C	2.1831366381	1.4315096079	-0.6133413873
O	3.5222206189	-2.8011064997	0.0192103916
C	3.9532904301	-3.5220891775	-1.0624642155
C	3.1733401316	-3.2582439592	-2.1435544756
C	2.1866435085	-2.3077901454	-1.7077230874
H	1.3983005131	-1.8651532881	-2.2993744062
H	3.2799472559	-3.6823325451	-3.1322083576
H	4.8081075115	-4.1597705741	-0.8936085827
C	-1.8232049123	-3.9205060228	0.3367813447
H	0.1514816316	-3.1471298234	0.5828336834
C	-0.7812426619	-3.2129920229	2.5284494461
H	-3.1113468059	-0.1301023276	0.5216773759
C	-3.3531762666	1.0243046287	-1.272103223
C	-3.0720473259	1.9414508583	1.1014133424
H	-0.4899795283	-4.2491328221	2.7297037898
H	-0.0595356188	-2.5527420656	3.0207170493
H	-1.7635249983	-3.0362067918	2.9803825554
H	-1.5173021998	-4.9591552349	0.501221986
H	-2.8326867565	-3.7869928751	0.7410889718
H	-1.8610012969	-3.7418197676	-0.742649572
H	-4.1579727444	2.0616822074	1.1687440134
H	-2.6944774336	1.7025850028	2.1009583844
H	-2.6350922283	2.8924299295	0.7850405386
H	-4.4437900035	1.0589524695	-1.1850940585
H	-3.0120005652	1.9598618465	-1.7209494642
H	-3.0922413036	0.1981771779	-1.9412849387

C	2.5032986221	2.8241679284	-0.9580179643
H	3.0386881284	0.7660126062	-0.6053914009
O	3.6973882882	2.8578694011	-1.5981118678
O	1.8750181779	3.8376598493	-0.6960197228
C	4.1824274023	4.1664106676	-1.9507842369
H	5.1435392207	4.0013913391	-2.4381204161
H	3.4926922997	4.6639708044	-2.6373709515
H	4.312394599	4.7851233566	-1.0590493928
H	-0.0672296708	4.3548321871	0.1842494921
O	-0.4513343168	4.9431894436	0.8567077163
C	0.3625794704	4.8416016281	2.0106675578
H	-0.0729715198	5.4835727492	2.7843366733
H	0.4077126591	3.8173594371	2.4137580203
H	1.3952803034	5.1775792258	1.834583077

17c_t2

C	-0.012660545	-0.4996300236	0.4519429254
N	1.3619307906	-0.1780426252	0.2916875567
N	-0.6369031749	-1.5882826961	0.618043724
N	-0.7051093781	0.7391331932	0.3940346286
C	-2.1898110839	0.8328635628	0.3175996129
C	1.5306155445	1.1725250875	0.1505156163
C	0.1655797308	1.7895308549	0.2436572968
C	-0.1017656012	-2.9491384492	0.6508966358
C	0.0720507901	3.1484179353	0.1838141576
C	2.5478293302	-1.0522912466	0.3606519056
C	3.0743646897	-1.4630542011	-0.9735412682
H	3.3259693458	-0.4730764501	0.8610940166
H	2.3152299162	-1.9072736435	0.9928878573
H	1.0120538891	3.6475752444	-0.025815125
C	-1.0440181073	4.0615342052	0.4277319433
O	2.6145124169	1.7334039743	0.0170256466
O	-0.6714683629	5.3219056879	0.0944855339
O	-2.1488154559	3.8147957115	0.8963126979
C	-1.6534404162	6.3472205708	0.3259726803
H	-1.1846613704	7.278812655	0.0084176049
H	-2.5563035407	6.1606783638	-0.2615545219
H	-1.9182322255	6.4007506292	1.3852336939
O	2.3442562358	-2.369757988	-1.6983523339
C	3.0224030787	-2.5953407064	-2.8628860144
C	4.1655856862	-1.8584425195	-2.8944683018
C	4.1978446255	-1.1184915636	-1.6658385687
H	4.9553175634	-0.4202244063	-1.3385450807
H	4.8950490147	-1.8389656514	-3.6920123931
H	2.5652729067	-3.2936482899	-3.5480529213
C	-1.0969886304	-3.8614516643	-0.07683539
H	0.8561571932	-3.0335939698	0.1298016958
C	0.0828899417	-3.3908649138	2.1098023561
H	-2.3730714073	1.8962367001	0.1934131062
C	-2.7522743672	0.1123117813	-0.9124382835

C	-2.8603946517	0.415467053	1.629502641
H	0.4167944576	-4.4329807673	2.1493301227
H	0.8263145148	-2.7739624693	2.6251320918
H	-0.8630875962	-3.3110457309	2.6566019469
H	-0.7406888483	-4.8970140534	-0.067026724
H	-2.0792305319	-3.8280624021	0.4074325809
H	-1.2176983311	-3.5533387658	-1.1204194053
H	-3.9315845595	0.6343141783	1.5665463541
H	-2.7355295824	-0.6499577299	1.8300436939
H	-2.4499716286	0.9843176223	2.4697032428
H	-3.8115883492	0.3709612809	-1.0137306406
H	-2.2396639503	0.4377284074	-1.823671981
H	-2.6691249569	-0.9725126943	-0.8308557844
H	3.1484560924	3.5075737203	-0.208385865
O	3.3505024321	4.4602422828	-0.2064100245
C	3.6954534267	4.8016912231	1.12872352
H	3.8892339615	5.8787999114	1.1589243465
H	2.8883175565	4.5840896874	1.8438662748
H	4.6042845217	4.2857050971	1.4739429195

E-**17c_t2**

C	0.856592133	-1.1624657596	0.3016186279
N	0.5317985256	0.2091056559	0.4250874776
N	1.9374126069	-1.8189662503	0.3815468688
N	-0.3733621518	-1.8166180969	0.0439322066
C	-0.4197394081	-3.2884901003	-0.1257246962
C	-0.8170323599	0.4224144466	0.2442782346
C	-1.4287882974	-0.9301817457	0.0163601188
C	3.2976475226	-1.3399885664	0.6178358097
C	-2.7309900842	-1.2257741652	-0.2420942839
C	1.4266958533	1.3460290764	0.703827303
C	1.9102333167	2.0566977143	-0.5170513273
H	0.8531600674	2.0634091279	1.293593081
H	2.2462918554	0.9927053997	1.3279203102
H	-2.9796732156	-2.2363427776	-0.5341397503
C	-3.8962914483	-0.3336583406	-0.1546160108
O	-1.3634943373	1.5167056523	0.2438462514
O	-4.8954947966	-0.8478894547	-0.9247631382
O	-4.0415394399	0.672176913	0.5161473877
C	-6.1379478583	-0.1263595316	-0.8896138036
H	-6.818302432	-0.6781042691	-1.5391345627
H	-6.538133972	-0.0870261466	0.1272735369
H	-6.0081636936	0.8933574327	-1.2623610418
O	2.7584762284	1.3839097806	-1.3592587766
C	3.0733961125	2.2374010328	-2.379273181
C	2.4513567616	3.4343776288	-2.2031331147
C	1.6910035061	3.3167026709	-0.9918247496
H	1.0598301661	4.0615728889	-0.5272979273
H	2.5198406228	4.2945722807	-2.8543569432
H	3.7371738936	1.8450812859	-3.1352695654

C	4.2455116221	-2.204193766	-0.2226193044
H	3.4348028949	-0.3025305916	0.2990493661
C	3.6302998982	-1.4501289792	2.1127427435
H	0.6171305954	-3.5826193535	0.0386250516
C	-0.7923386889	-3.6943136036	-1.5568755165
C	-1.2703764725	-3.9708408946	0.9517831178
H	4.6727447506	-1.1659417743	2.2909483369
H	2.9904809891	-0.7965968389	2.7147584007
H	3.488803382	-2.4793121089	2.4607659352
H	5.2831546071	-1.8847389839	-0.0790752999
H	4.1636462077	-3.2587833962	0.0634194749
H	4.0061029395	-2.1197990111	-1.287679076
H	-1.131406791	-5.0538585374	0.8750831219
H	-0.9522068978	-3.6587032476	1.951342924
H	-2.3389695515	-3.7664798768	0.850292767
H	-0.6585278704	-4.7754879894	-1.6642335822
H	-1.8294092024	-3.4626782248	-1.8132678809
H	-0.1392429715	-3.1986122246	-2.2816351311
H	-0.9403219744	3.2314530188	0.83684576
O	-0.629379861	4.053297987	1.2571076961
C	-1.5127873125	4.3501128274	2.3301518226
H	-1.1302446196	5.2389634507	2.8417019676
H	-2.5334038404	4.5710216259	1.9838054099
H	-1.5711555096	3.53413371	3.0650955534

18c_t1

C	-0.2805396825	-0.8482218178	0.4628062791
N	1.0882546033	-0.581453823	0.1514944127
N	-0.8181464932	-1.9295343683	0.8469576902
N	-1.1548928708	0.2856376172	0.3204039468
C	-2.6300222258	0.0420454023	0.4495143525
C	1.5210753999	0.7046477502	-0.1216176423
C	-0.7193926779	1.5871679483	0.1586001534
C	-0.4216539843	-3.3197089381	1.011408129
O	-1.5090934977	2.5488849746	0.1972520118
C	2.0960847162	-1.6187038025	0.4324516691
C	2.2961623758	-2.5846190972	-0.6964147258
H	3.0383932988	-1.1235117324	0.6748031357
H	1.8064074842	-2.146286568	1.3424830387
C	0.6907349045	1.7686355869	-0.0961809342
O	3.0428959519	-3.6867647703	-0.377873284
C	3.1380277184	-4.4504820352	-1.510732979
C	2.4655129753	-3.8581294214	-2.5326571529
C	1.9169336168	-2.6398206538	-2.0040131454
H	1.3326326303	-1.9016036401	-2.5333764944
H	2.3686214581	-4.2342439961	-3.5416114034
H	3.7062571839	-5.3638024007	-1.4175466252
C	-1.4602799402	-4.1932897043	0.2927266046
H	0.5502834569	-3.5595806365	0.5740012519
C	-0.3728265545	-3.649665457	2.5100950472

H -2.7043182756 -1.0416683662 0.4645366686
C -3.4043300095 0.5470290491 -0.7725156351
C -3.1764388916 0.5624327048 1.7823333578
H -0.1438363699 -4.7102014317 2.6587140605
H 0.3936185568 -3.0589017499 3.022918233
H -1.3384521504 -3.4340432073 2.9808352638
H -1.2038789761 -5.2530488344 0.3989394354
H -2.4600736342 -4.0347600893 0.7118372726
H -1.4938848982 -3.956058942 -0.7756305108
H -4.2261653616 0.2652939204 1.880600343
H -2.6258224121 0.1280904458 2.6230443409
H -3.1178999433 1.6505614617 1.8470113251
H -4.4282902952 0.1621502008 -0.715773561
H -3.4454477754 1.6354876835 -0.822166903
H -2.9577121856 0.1691173931 -1.6983743665
H 1.0392285957 2.7697669963 -0.3071277224
C 2.9411812829 0.9270289569 -0.5816033748
O 3.4542947496 2.0278069123 -0.0249792282
O 3.5123623564 0.2285634244 -1.3940245397
C 4.7721052165 2.4114759108 -0.4877656346
H 5.0222515442 3.3114711215 0.0720665888
H 5.4923183969 1.6178517698 -0.278711851
H 4.7513958306 2.6205470352 -1.5594986967
H -0.7351075865 4.1588199402 -0.1127002513
O -0.1793178256 4.9620793933 -0.1931844116
C -0.0056882642 5.4693121562 1.1197942695
H 0.6456973811 6.3478605225 1.0600103249
H -0.9544703493 5.786961506 1.5799742845
H 0.4707294461 4.7445666994 1.7978455822

18c_t2

C -0.0574174078 -1.0076193018 0.5413978383
N 1.342277713 -1.07902995 0.3011153057
N -0.8786290988 -1.9446914121 0.7916538911
N -0.6021702498 0.3224675801 0.5084474655
C -2.1032353241 0.4227491949 0.5927710746
C 2.1421344045 0.0056509987 -0.0835875969
C 0.1958533343 1.4013399238 0.2477082779
C -0.79007339 -3.3980208663 0.7232502096
C -0.2943457848 2.8079607044 0.5044026862
C 2.0968828221 -2.3052301938 0.5966059585
C 2.3756875447 -3.1414170166 -0.6167195329
H 3.0483907014 -1.9944867491 1.0352432141
H 1.5642737045 -2.8727626769 1.3582972522
C 1.5038972483 1.2883580452 -0.1040730386
O 3.1779538495 -4.2262584124 -0.3927393306
C 3.3425127632 -4.8630523487 -1.5941770514
C 2.664193133 -4.2043282688 -2.5701675548
C 2.0326532252 -3.0798460713 -1.9348388235
H 1.4147954524 -2.3274017092 -2.4034272003

H	2.6146730259	-4.4769188006	-3.6152079096
H	3.9570360612	-5.7506800886	-1.5774136183
C	-1.9840618414	-3.8980988638	-0.1039034804
H	0.11402951	-3.7543432904	0.220224619
C	-0.8354820082	-3.9817990575	2.142473898
H	-2.4217154719	-0.5623087707	0.2589260216
C	-2.7081843644	1.4430920494	-0.3768892028
C	-2.5797695898	0.5940609316	2.0369836383
H	-0.8294984636	-5.0762112133	2.1052786621
H	0.0207125463	-3.6560890226	2.7425724599
H	-1.7476230657	-3.6586310702	2.6566413088
H	-1.9639920196	-4.9908754416	-0.1790281634
H	-2.930663739	-3.5999822127	0.3603730569
H	-1.954822153	-3.4861133799	-1.1179582514
H	-3.67442975	0.5629488561	2.0607071519
H	-2.2065512953	-0.2207937977	2.6634448717
H	-2.2489582823	1.5457036986	2.4575013856
H	-3.7692932483	1.199062924	-0.4883246068
H	-2.6606203689	2.474927323	-0.022898406
H	-2.2492268992	1.3805959288	-1.3683101641
H	2.117168409	2.1540161966	-0.3115357086
O	3.3481040431	-0.171844425	-0.3211278706
O	-0.072651054	3.5937584543	-0.5506987738
O	-0.7399865186	3.1803662776	1.5708218421
C	-0.3713493875	4.9998342509	-0.3671126843
H	-0.1268523741	5.471392525	-1.3177660606
H	-1.4294776989	5.1356086615	-0.1340984171
H	0.2415687667	5.4153827982	0.4354476288
H	4.2499273382	1.3849295162	-0.6497345999
O	4.6172051736	2.2913620801	-0.6933807027
C	5.2236032969	2.5456901298	0.5635905851
H	5.6075353416	3.5713667195	0.5520708528
H	4.5165540844	2.4618188147	1.4031459882
H	6.0711373557	1.873151829	0.768639605

S8. References

1. Ribeiro, R.F.; Marenich, A.V.; Cramer, C.J. Truhlar, D.G., Use of Solution-Phase Vibrational Frequencies in Continuum Models for the Free Energy of Solvation, *J. Phys. Chem. B*, **2011**, *115*, 14556–14562. DOI: 10.1021/jp205508z
2. Molteni, G.; Ponti, A. The Azide-Allene Dipolar Cycloaddition: Is DFT Able to Predict Site- and Regio-Selectivity? Molecules 2021, 26, 928. DOI: 10.3390/molecules26040928
3. Yepes, D.; Valenzuela, J.; Martínez-Araya, J.I.; Pérez, P.; Jaque, P. Effect of Exchange-Correlation Functional on theSynchronicity/Nonsynchronicity in Bond Formation in Diels-Alder Reactions: A Reaction Force Constant Analysis, *Phys. Chem. Chem. Phys*. **2019**, *21*, 7412-7428, DOI: 10.1039/C8CP02284D.
